# Mechanistic insights into Jianpi Qinghua Sanyu Yin treatment of raised erosive gastritis: ceRNA-mediated PI3K/AKT signaling pathways

**DOI:** 10.3389/fphar.2025.1495020

**Published:** 2025-03-03

**Authors:** Liya Liu, Ying Cheng, Guosheng Lin, Huifang Zheng, Xinran Zhang, Peilin Zhao, Meixuan Guo, Qihong Liu, Wenyi Fang, Wenrong Wang, Anjum Jafri, Aling Shen, Xiao Ke, Youqin Chen

**Affiliations:** ^1^ Academy of Integrative Medicine, Fujian University of Traditional Chinese Medicine, Fuzhou, China; ^2^ Fujian Key Laboratory of Integrative Medicine in Geriatrics, Fujian University of Traditional Chinese Medicine, Fuzhou, Fujian, China; ^3^ Affiliated Sanming Integrated Medicine Hospital of Fujian University of Traditional Chinese Medicine, Sanming, Fujian, China; ^4^ Department of Gastroenterology, The Second People’s Hospital Affiliated to Fujian University of Traditional Chinese Medicine, Fuzhou, Fujian, China; ^5^ Fujian Clinical Medical Research Centre of Chinese Medicine for Spleen and Stomach, The Second People’s Hospital Affiliated to Fujian University of Traditional Chinese Medicine, Fuzhou, Fujian, China; ^6^ Histology Core, Department of Genetics and Genome Sciences, Case Western Reserve University, Cleveland, OH, United States; ^7^ Department of Pediatrics, Case Western Reserve University School of Medicine, Rainbow Babies and Children’s Hospital, Cleveland, OH, United States

**Keywords:** Jianpi Qinghua Sanyu Yin, raised erosive gastritis, ceRNA, miR-122-5p, PI3K/Akt signaling pathways

## Abstract

**Background:**

Raised erosive gastritis (REG) is a chronic gastritis with a high risk of malignant transformation. Current treatments often result in high recurrence rates and complications. Jianpi Qinghua Sanyu Yin (JPQHSYY), a traditional Chinese medicine, shows promise in treating REG. However, the underlying molecular mechanisms remain unclear. This study aimed to investigate the potential mechanism of JPQHSYY’s therapeutic effects on REG.

**Methods:**

RNA-seq was employed to systematically analyze mRNA, lncRNA, and miRNA profiles in gastric mucosal tissues from REG patients before and after JPQHSYY treatment. The pivotal lncRNA-miRNA and miRNA-mRNA networks were predicted from sequencing data and bioinformatic analysis, and the results were exported using Cytoscape software. Gene Ontology (GO) analysis and Kyoto Encyclopedia of Genes and Genomes (KEGG) analyses were used for functional exploration. Real-time quantitative reverse transcription-polymerase chain reaction (qRT-PCR) was performed to validate RNA-seq analysis results. CCK8, cell cycle, apoptosis and western blot were performed to detect the effects of miR-122-5p in GES-1 cells *in vitro*.

**Results:**

RNA-seq analysis revealed 576 differentially expressed lncRNAs (269 upregulated, 307 downregulated), 33 differentially expressed miRNAs (13 upregulated, 20 downregulated), and 1717 differentially expressed mRNAs (777 upregulated, 940 downregulated) in JPQHSYY-treated REG patients. GO and KEGG analyses highlighted key pathways, including the PI3K/AKT signaling pathway, involved in cell cycle and apoptosis regulation. The ceRNA network analysis suggested that JPQHSYY impacts the miRNA–lncRNA interactions. Validation experiments confirmed that JPQHSYY inhibits the PI3K/AKT pathway, reducing cell viability, colony formation, and promoting apoptosis in miR-122-5p transfected GES-1 cells.

**Conclusion:**

The therapeutic efficacy of JPQHSYY in treating REG might be mediated by the ceRNA-driven PI3K/AKT pathway signaling pathways, which is implicated in the proliferation of gastric mucosal epithelial cells. Furthermore, the investigation of miRNA–lncRNA networks could reveal more information on potential new mechanisms and targets for JPQHSYY in the management of REG.

## 1 Introduction

Raised erosive gastritis (REG) is a special type of chronic gastritis with a high incidence rate (Xiao et al., 2022; Hirano et al., 2022). It has been recognized as one of the diseases with a high potential for malignant transformation (Zhang et al., 2005). Current clinical treatments for REG include endoscopic therapy, anti-Helicobacter pylori therapy, acid suppression, and gastric mucosal protection. However, these treatments often result in high recurrence rates and are associated with numerous complications (Desmond and Reynolds, 1972; Yang et al., 2024). These challenges underscore a urgent need for more effective therapeutic approaches with lower recurrence rates and fewer adverse effects.

Jianpi Qinghua Sanyu Yin (JPQHSYY), an empirical formula developed by the national medical master Chunbo Yang, composed of various traditional Chinese medicines, including *Codonopsis*, *Artemisia capillaris*, *Carapax Trionycis*, *Atractylodes macrocephala*, *Hyacinth Bean*, *Poria*, *Dried Orange Peel*, *Pinellia*, *Magnolia officinalis*, *Coptis chinensis*, *Curcuma zedoaria*, *Salvia miltiorrhiza*, *Amomum villosum*, and *Glycyrrhiza uralensis* (Lin et al., 2016). It is primarily prescribed to treat REG patients exhibiting symptoms of spleen deficiency, damp-heat, and blood stasis. Clinical studies have reported that JPQHSYY has a cure rate of 67.5% and a total effective rate of 82.5%, significantly superior to the western medicine control group (Lin et al., 2013). Previous studies have found that JPQHSYY reduces the expression of bFGF, FGFR2, PCNA, Bcl-2, and EGF while increasing Bax expression, contributing to its therapeutic effects on REG (Fang et al., 2017; Mao et al., 2019; Lin et al., 2016). However, the detailed molecular mechanisms underlying JPQHSYY’s efficacy remain inadequately understood, particularly its regulatory networks.

Noncoding RNAs, such as long noncoding RNAs (lncRNAs), microRNAs (miRNAs), and circular RNAs (circRNAs), have recently been discovered their important roles in a variety of biological processes such as apoptosis and cell proliferation (Nemeth et al., 2024; Zhao et al., 2020). lncRNAs, initially considered as “transcription noise”, have been found to regulate gene expression at multiple levels, influencing cells processes and organismal function (Quinn and Chang, 2016). Intriguingly, emerging evidence indicates that lncRNAs play key role in gastritis pathogenesis (Guo et al., 2023; Qin et al., 2021). Similarly, miRNAs, which are small, noncoding single-stranded RNAs, regulate the expression of target genes at the post-transcriptional level (Li et al., 2018), and have been implicated in tumor progression as both oncogenes and tumor suppressors (Pradhan et al., 2017; Biswas, 2018). MiRNAs act by binding to complementary sequences in the 3′ untranslated region (UTR) of their target mRNAs, leading to transcript degradation or translation repression (Pang et al., 2010). This mechanism, where lncRNAs and circRNAs regulate miRNA levels and influence gene expression, has been implicated in the pathogenesis of gastric diseases (Mao et al., 2017). Several dysregulated miRNAs, such as miR-122-5p, miR-21, miR-135b, miR-196a-5p, have been associated with gastritis through regulating cell proliferation, migration, and invasion (Săsăran et al., 2021; Zingone et al., 2022), and miR-122-5p is considered as a potential biomarker for early detection of gastritis and gastric cancer (You et al., 2021; Liu et al., 2021; Zhu et al., 2019). By competitively binding to miRNAs along with their miRNA response regions, lncRNAs and circRNAs can function as competing endogenous RNAs (ceRNAs), controlling the miRNA expression levels targeting mRNAs (Panda, 2018). This mechanism has been implicated in gastric diseases, but the interplay between miRNAs and lncRNAs in REG, partcularly in the context of JPQHSYY treatment, remain poorly understood. Exploring these interactions could provide novel insights into the molecular mechanisms underlying the therapeutic effects of JPQHSYY.

This study addresses three key knowledge gaps in understanding JPQHSYY’s role in REG treatment: (1) elucidating the comprehensive molecular profile associated with its therapeutic effects, (2) investing the ceRNA networks mediating these effects, and (3) identifying specific pathways, such as PI3K/AKT pathway, modulating by JPQHSYY. We hypothesize that JPQHSYY exerts its therapeutic effect by influencing the miRNA–lncRNA interactions. Using transcriptome sequencing and bioinformatics analysis, this study aims to elucidate the expression profiles of lncRNAs, miRNAs and mRNAs, and the miRNA–lncRNA ceRNA-mediated regulatory pathways in JPQHSYY-treated REG patients. This exploratory approach will lay the foundation for future studies and provide theoretical and experimental evidence for the clinical application of JPQHSYY in treating REG.

## 2 Materials and methods

### 2.1 Reagents

The normal human gastric mucosal epithelial cell line GES-1 was obtained from the Cell Bank of the Chinese Academy of Sciences (Shanghai, China). Cell lysis buffer (Cat No. P0013), ECL luminescent reagent kit (Cat No. P0018) and PMSF (Cat No. P0100) were purchased from Beyotime Biotechnology (Shanghai, China). PhosSTOP™ (Cat No. 4906837001) was acquired from Roche (Auckland, New Zealand). Protein marker (Cat No. 26616) and BCA protein quantification reagent kit (Cat No. 23225) were sourced from Thermo Fisher Scientific (Waltham, MA, United States). A polyvinylidene fluoride (PVDF) membrane (Cat No. ISEQ00010) was procured from Merck Millipore (Burlington, MA, United States). Hsa-miR-negative control (NC; Cat No. miR1N0000001-1-5) and hsa-miR-122-5p mimic (Cat No. miR10000421-1-5) were supplied by Ribo Biotechnology (Guangzhou, China). Bax (Cat. no. 3722), p-PI3K (Cat No. 4228), PI3K (Cat No. 4257), p-Akt (Cat No. 4060) and Akt (Cat No. 4691) were purchased from Cell Signaling Technology (Beverly, MA, United States). Bcl2 (Cat. no. ABM0010), PTEN (Cat. no. ABP0181) and GAPDH antibody (Cat. no. ABL1021) was sourced from Abbkine Scientific (Wuhan, Hubei, China).

### 2.2 Subjects and samples

The RNA-Seq cohort included five REG patients before and after treatment. Gastric mucosal tissues were biopsied from the same elevated site before and after treatment. Biopsy samples were obtained using standard endoscopic biopsy forceps during upper gastrointestinal endoscopy, with endoscopic photographs taken for precise documentation of the biopsy sites. The procedures for pre- and post-treatment biopsies were as follows:

Pre-treatment biopsies: These were taken after a through endoscopic examination of the lesion to ensure accurate sampling. For post-treatment sampling, we followed these specific steps: (1) The follow-up endoscopy was conducted 8–12 weeks after the initial treatment to ensure adequate healing of the gastric mucosa. (2) The biopsy site was carefully located using a combination of previously captured endoscopic images and recorded measurements for accurate repositioning. (3) Once identified, biopsy samples were obtained following the same protocols as pre-treatment procedure.

Immediately following excision, the tissues were placed in cryotubes and stored in a −80°C freezer for RNA sequencing, resulting in a total of 10 tissue samples. The inclusion criteria for REG patients were as follows:

(1) Age between 18 and 65 years, with female patients required to have a negative pregnancy test result; (2) Presence of single or multiple verrucous, enlarged folds or papules in the gastric antrum and/or gastric mucosa, approximately 5–10 mm in diameter, with mucosal defects or umbilical depressions at the top and erosions in the center; (3) Voluntary participation, signing of the informed consent form, and agreement to prescribed continuous medication and regular follow-up and reexamination. The exclusion criteria for REG patients were as follows: (1) Age below 18 or above 65 years, and pregnant or lactating women; (2) Patients with severe primary diseases affecting the cardiovascular, hepatic, renal, hematopoietic systems, or mental illness; (3) Patients with gastric ulcer, duodenal ulcer, or reflux esophagitis.

All samples were collected following written informed consent from each participant. The study was approved by the Ethics Committee of the Second People’s Hospital affiliated with Fujian University of Traditional Chinese Medicine (SPHFJP-Y2021026-02), adhering to the Declaration of Helsinki.

### 2.3 RNA sequencing

The gastric mucosal tissues were dissected and initially stored in RNAlater (Takara Biotechnology; Dalian, Liaoning, China) at room temperature for 1 h, and then transferred to −20°C for long-time storage. RNA isolation and RNA sequencing (RNA-seq) library preparation were performed by CapitalBio Technology (Beijing, China).

#### 2.3.1 RNA extraction and quality examination

RNA extraction was performed using the traditional TRIzol procedure with TRIzol reagent (Thermo Fisher Scientific). RNA degradation and contamination were detected by 1% agarose gels. RNA purity was checked using the kaiaoK5500^®^ Spectrophotometer (Kaiao, Beijing, China). RNA concentration was measured using Qubit^®^3.0 Flurometer (Life Technologies, CA, United States). RNA integrity was assessed using the RNA Nano 6000 Assay Kit on the Bioanalyzer 2100 system (Agilent Technologies, United States).

#### 2.3.2 Preparation and examination of the cDNA library for high-throughput RNA sequencing

A total of 3 µg RNA per sample was used as initial material to construct lncRNA, miRNA and mRNA libraries. Ribosomal RNA was removed using Epicentre Ribo-Zero™ Gold Kits. The sequencing libraries were generated following manufacturer recommendations with varied index label by NEBNext^®^ Ultra™ Directional RNA Library Prep Kit for Illumina (NEB, Ispawich, United States). RNA concentration of the library was preliminarily measured using Qubit^®^ 3.0, and then dilute to 1 ng/μL. Insert size was assessed using the Agilent Bioanalyzer 2100 system, and qualified insert size was accurately quantified using Taqman fluorescence probes on AB Step One Plus Real-Time PCR system (Library valid concentration>10 nM). After cluster generation, the sequencing of the cDNA library was carried out by OE Biotech (Shanghai, China) on an Illumina Hiseq 4000 platform, generating 150bp paired-end reads.

#### 2.3.3 Quality control of transcriptome sequencing

Low-quality tags in the raw data were removed, and ribosomal RNA data were also removed from the remaining data by alignment. The 150 bp paired-end reads were checked with FastQC. For transcriptome project, an overall assessment was carried out using RSeqQC from three aspects: sequencing saturation, randomness of sequencing library construction, and enrichment of reads in different elements of the genome.

#### 2.3.4 Identification of lncRNAs

StringTie software was used to reconstruct transcripts in each sample based on probability model. Cuffcompare software was used to compare merged transcripts with reference transcripts. Known lncRNAs and similar transcripts to other lncRNAs and mRNAs were screened out, retaining transcripts based on lncRNAs characteristics. The length>200bp and exon number≥2 were adopted to obtain candidate lncRNAs. Additionally, CPC (Coding Potential Calculator) analysis, CNCI (Coding-Non-Coding Index) analysis and PFAM protein domain analysis were applied to comprehensively predict the coding ability of candidate lncRNAs. Corresponding results were displayed through Venn diagrams.

#### 2.3.5 Screening on differentially expressed lncRNAs, miRNAs, and mRNAs

Gene expression was calculated using FPKM method, which consider the impact of sequencing depth and gene length on fragment count, and can be directly used for comparing gene expression between different samples. The differential expression of genes was calculated using the negative binomial distribution test in DESeq software. The significance of the difference was tested, and gene expression was displayed by base mean value. The osteogenic-specific induction groups and control groups were compared to search for differentially expressed genes, including lncRNAs, miRNAs and mRNAs, using |Foldchange|≥2 and P ≤ 0.05 as the limitations.

#### 2.3.6 GO enrichment and Pathway enrichment

GO enrichment analysis, which provides three categories on biological process (BP), cellular component (CC), and molecular function (MF), was performed to describe meaningful annotation of genes and gene products attributes, with P ≤ 0.05 considered statistically significant. The top 30 GO terms for each category were ranked by − log10 (P value), reflecting gene count and enrichment score.

Pathway enrichment analysis was performed using the latest Kyoto Encyclopedia of Genes and Genomes (KEGG) database, searching for probable cellular pathways associated with differentially expressed genes, with P ≤ 0.05 considered statistically significant. The top 30 KEGG enrichments ranked by − log10 (P value) showed the significance order of the correlated biological pathway.

#### 2.3.7 ce-RNA (miRNA-lncRNA) regulatory network

According to the expression levels of miRNA and lncRNA, the Pearson correlation coefficient and p-value of miRNA-target were calculated. Negatively correlated pairs with p-value <0.05 and Pearson correlation coefficient >0.85 were selected for further analysis. The miRNA-target genes were predicted by miRanda software, and the intersection with the results of the previous step was taken to construct the miRNA-gene expression regulatory network. Based on the co-expression data, the regulatory interaction relationship between lncRNA and miRNA was screened out, imported into Cytoscape software, and the relevant ceRNA regulatory network was constructed.

### 2.4 Quantitative real-time reverse transcription polymerase chain reaction (qRT-PCR)

Total RNA was extracted from gastric mucosal tissues using RNAiso Plus reagent (Takara Biotechnology) and quantified with a NanoDrop 2000 spectrophotometer (Thermo Fisher Scientific). Reverse transcription of the RNA into cDNA was carried out using the PrimeScript RT reagent kit, following the manufacturer’s instructions. The cDNA was then amplified using a quantitative PCR instrument with TB Green^®^ Premix Ex Taq. The PCR reaction protocol included an initial denaturation at 95°C for 30 s, followed by 40 cycles of 95°C for 5 s and 60°C for 30 s. Relative mRNA levels were calculated using the comparative 2^^ΔΔCt^ method, with GAPDH serving as the internal reference gene. The primer sequences used were as follows: BAX (F: CCC​GAG​AGG​TCT​TTT​TCC​GAG, R: CCA​GCC​CAT​GAT​GGT​TCT​GAT), BCL2 (F: GGT​GGG​GTC​ATG​TGT​GTG​G, R: CGG​TTC​AGG​TAC​TCA​GTC​ATC​C), PTEN (F: TGG​ATT​CGA​CTT​AGA​CTT​GAC​CT, R: GGT​GGG​TTA​TGG​TCT​TCA​AAA​GG), PI3K (F: ACC​ACT​ACC​GGA​ATG​AAT​CTC​T, R: GGG​ATG​TGC​GGG​TAT​ATT​CTT​C), and AKT (F: AGC​GAC​GTG​GCT​ATT​GTG​AAG, R: GCC​ATC​ATT​CTT​GAG​GAG​GAA​GT).

### 2.5 Preparation of JPQHSYY

JPQHSYY was provided by the Second People’s Hospital affiliated with Fujian University of Traditional Chinese Medicine. The basic prescription includes: *Codonopsis* 15g, *Artemisia capillaris* 9g, *Carapax Trionycis* 18g, *Atractylodes macrocephala* 9g, *Hyacinth Bean* 15g, *Poria* 15g, *Dried Orange Peel* 9g, *Pinellia* 9g, *Magnolia officinalis* 9g, *Coptis chinensis* 3g, *Curcuma zedoaria* 9g, *Salvia miltiorrhiza* 15g, *Amomum villosum* 6g, and *Glycyrrhiza uralensis* 3g. These ingredients were boiled three times in 2L distilled water, then concentrated and mixed into granules. The granules were ground into powder and dissolved in high-pressure water to prepare a stock solution with a concentration of 10 mg/mL, sonicated for 30 min, sterilized under high pressure, aliquoted into 1.5 mL EP tubes, and stored at 4°C.

### 2.6 Cell culture and transfection

GES-1 cells were cultured in DMEM high-glucose medium containing 10% fetal bovine serum, 100U/mL penicillin, and 100 μg/mL streptomycin, in a 37°C incubator with 5% CO_2_. When cells reached confluence, they were passaged using 0.25% trypsin. Cells were seeded into appropriate culture plates at specific densities. After overnight culture, cells were transfected with miR-NC and miR-122-5p mimic (50 nM) following the manufacturer’s instructions, and treated with different concentrations (25, 50, 100 μg/mL) of JPQHSYY.

### 2.7 Observation of cell growth and cell counting

After transfection with miR-NC and miR-122-5p mimic and treatment with different concentrations (25, 50, 100 μg/mL) of JPQHSYY, cell morphology and growth were observed under an inverted microscope. Cells were digested with 0.25% trypsin, centrifuged, and resuspended in 1 mL complete medium. A small volume of cell suspension was mixed with an equal volume of trypan blue and counted using a Constar cell counter.

### 2.8 GES-1 cell viability assay

Following transfection and treatment as described, 10 μL of CCK-8 reagent was added to each well, and cells were incubated at 37°C for 2 h. Absorbance values were measured with a microplate reader, and proliferation curves were plotted based on these values.

### 2.9 Colony formation assay

Cells were collected and counted after transfection and treatment. Approximately 0.5 × 10^3^ cells were seeded per well in 12-well plates and cultured for 8–10 days (with medium changes every 2–3 days). After culture, cells were washed three times with PBS, fixed with 4% paraformaldehyde for 15 min, washed again with PBS, and stained with crystal violet for 15 min. Following three washes with PBS, plates were dried and photographed. Colony numbers were counted, and cell survival rates were calculated relative to the control group.

Cell Survival Rate Calculation Formula: Cell survival rate (%) = (number of colonies in experimental group/number of colonies in control group) × 100%

### 2.10 PI staining and flow cytometry for cell cycle analysis

Following transfection and treatment, cells were collected and fixed overnight in 70% ethanol at 4°C. RNase staining solution (500 μL) was added, and cells were incubated for 30 min. DNA content and cell cycle distribution were analyzed using flow cytometry and ModfitLT Version 3.0 software.

### 2.11 Annexin V/PI staining and flow cytometry for apoptosis detection

Cells were collected, washed once with cold PBS, resuspended in 100 μL 1× Assay Buffer, and stained with 5 μL Annexin V-647 and 2 μL PI. After 15 min incubation in the dark at room temperature, 400 μL 1x Assay Buffer was added, and apoptosis was analyzed using flow cytometry.

### 2.12 Western blot

After transfection and treatment, cells were lysed with buffer containing protease and phosphatase inhibitors. Total protein content in the supernatant was quantified using a BCA protein assay kit. Protein samples were denatured with 5× SDS and separated by SDS-PAGE, then transferred to PVDF membranes. Membranes were blocked with 5% non-fat milk in TBST for 1 h, and incubated overnight at 4°C with primary antibodies (PCNA: 1:1000; Bax: 1:1000; Bcl2: 1:1000; p-PI3K: 1:1000; PI3K: 1:1000; p-Akt: 1:1000; Akt: 1:1000; PTEN: 1:1000). After three washes, membranes were incubated with HRP-conjugated secondary antibodies at room temperature for 1 h. Protein expression was detected using a chemiluminescence imaging system, and optical densities were measured with ImageJ software.

### 2.13 Statistical analysis

All data are presented as the mean ± standard deviation with 95% confidence intervals where applicable. The Shapiro-Wilk test was used to assess the normality of data for experiments involving three or more groups. For normally distribution data, comparisons were conducted using one-way ANOVA, with the LSD test applied for homogeneous variance and Games Howell test for heterogeneous variance. Non-normally distributed data were analyzed using the Kruskal–Wallis test, followed by Dunnett’s *t*-test for pairwise comparisons. For RNA-seq data analysis, differential gene expression was evaluated using DESeq2, with statistical significance determined by an adjusted P-value (FDR) < 0.05 and |log2FoldChange| > 1 as cutoff criterion. Differences were considered statistically significant at P < 0.05. All statistical analyses were performed using SPSS version 26.0 (IBM Corporation, Armonk, NY, United States) and GraphPad Prism version 5.0 (GraphPad Software, San Diego, CA, United States).

## 3 Results

### 3.1 lncRNAs sequencing data analysis

**FIGURE 1:**
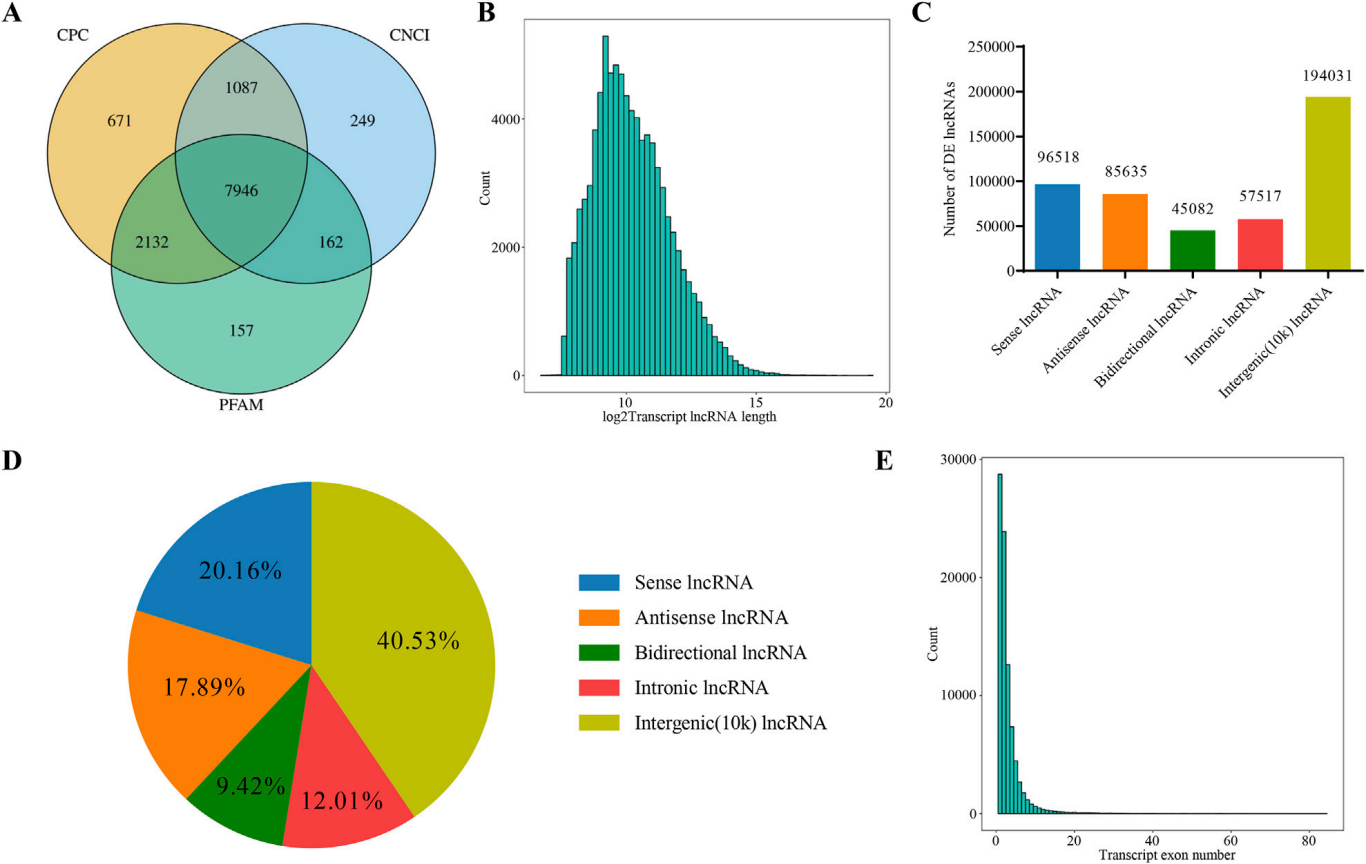
Analysis of lncRNAs Sequencing Data. **(A)** Venn diagram showing the number of novel lncRNAs predicted by three different methods. **(B)** Distribution of lncRNA transcript lengths. The horizontal axis represents the logarithm of transcript length (base 2), and the vertical axis represents the number of transcripts. **(C)** Classification of lncRNAs based on their genomic location, showing the number of lncRNAs for each type. **(D)** Classification of lncRNAs based on their genomic location, showing the proportion of lncRNAs for each type. **(E)** Distribution of the number of exons in lncRNA transcripts. The horizontal axis represents the number of exons per transcript, and the vertical axis represents the corresponding number of transcripts.

cDNA libraries were constructed for RNA-seq to analyze relevant lncRNAs information in gastric mucosal tissue samples from five patients before and after treatment. In total, 478,783 lncRNAs were detected based on four algorithms, CPC, CNCI, PFAM analysis, with the results exported via Veen analysis (Figure 1A). The feature analysis of lncRNA was conducted based on length distribution and number of exons. According to their length distribution, most lncRNAs were shorter than 2000 nt (Figure 1B). Based on the genomic location of lncRNAs, 96,518 were classified as Sense (20.16%), 85,635 as Antisense (17.89%), 45,082 as Bidirectional (9.42%), 57,517 as Intronic (12.01%) and 19,4031 as Intergenic (10k) (40.53%) (Figures 1C, D). Intergenic lncRNAs accounted for the largest proportion, while Bidirectional lncRNAs represented the lowest proportion. In terms of the number of lncRNA exons, those with one exon were the most abundant, followed by those with two and three exons (Figure 1E).

### 3.2 miRNA sequencing data analysis

**FIGURE 2:**
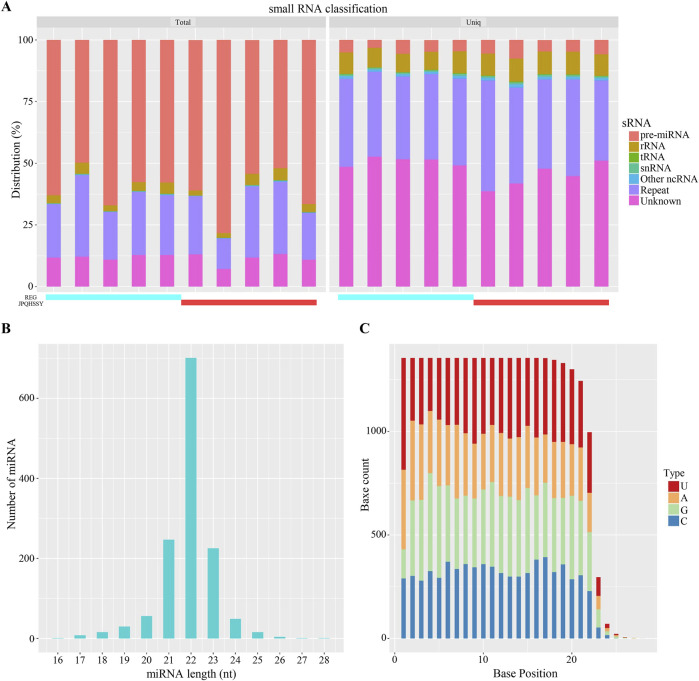
Analysis of miRNA Sequencing Data. **(A)** Distribution statistics of small RNAs. Different colors represent various types of small RNAs. The horizontal axis indicates the sample, while the vertical axis shows the percentage of each small RNA type. **(B)** Length distribution of miRNAs. The horizontal axis represents the length of miRNAs, and the vertical axis shows the number of miRNAs at each length. **(C)** Base distribution of miRNAs. The horizontal axis denotes the base position, and the vertical axis indicates the number of occurrences of each base at that position, with each color representing a different base.

The miRNA expression profile of gastric mucosal tissue samples from five patients before and after treatment was obtained using a high-throughput sequencing platform. Firstly, the Clean Data length distribution of each sample was statistically analyzed and classified (Figure 2A). By comparing with the database, rRNA, tRNA, snRNA, and other ncRNAs were filtered out. The subsequent Clean Data serve as candidate sequences for predicting miRNA. Among these, the known pre-miRNA of this species was compared as known miRNA reads. Most miRNA lengths were 22 nt, which aligns with the typical miRNA length (Figure 2B). Furthermore, the first nucleotide bias of miRNAs indicated that a large proportion of first nucleotide biases were guanine (G), consistent with the miRNA typical characteristic (Figure 2C).

### 3.3 lncRNAs, miRNAs and mRNAs expression profiles

**FIGURE 3:**
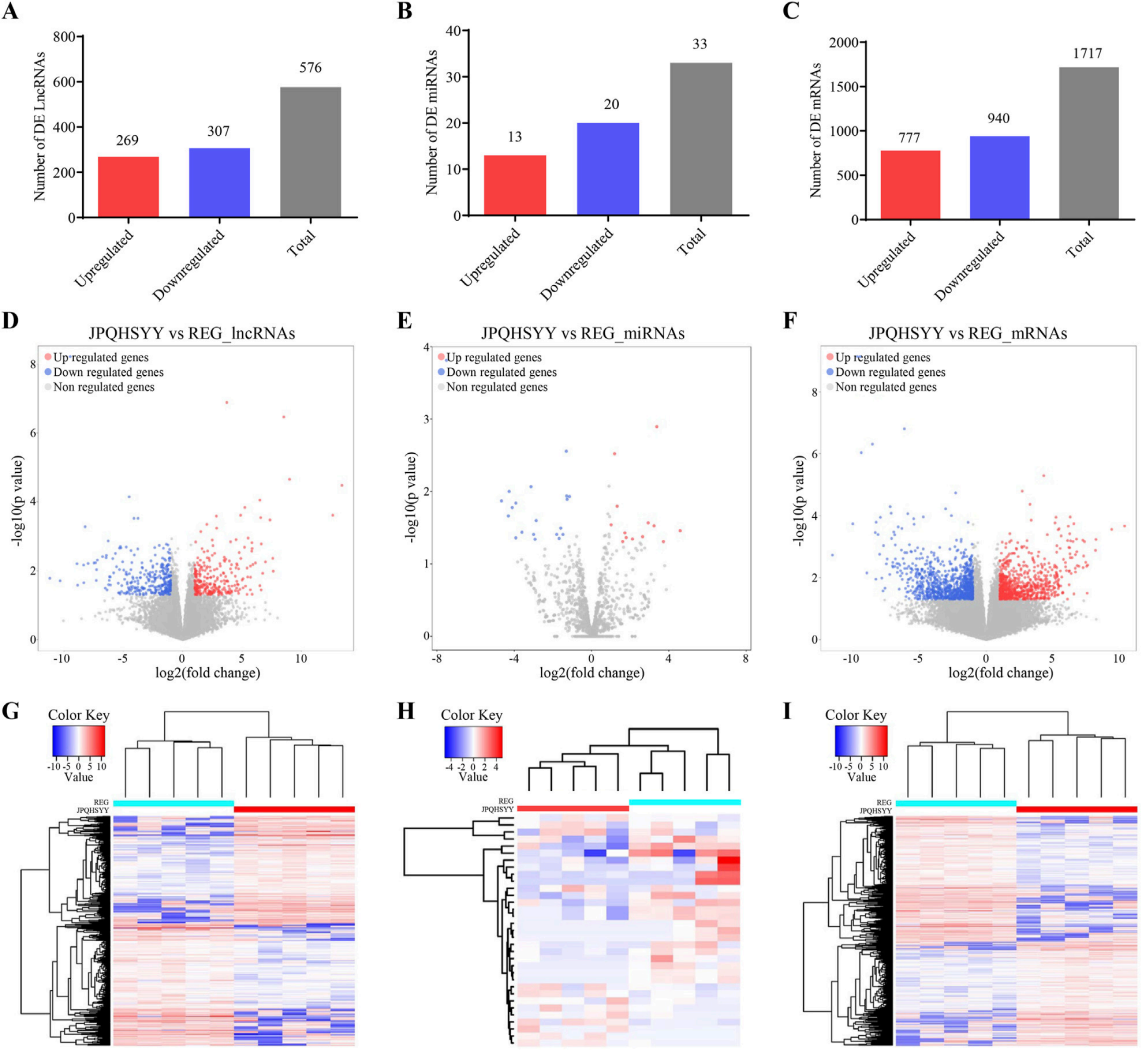
Expression Profiles of lncRNAs, miRNAs, and mRNAs. **(A–C)** Detection of differentially expressed **(A)** lncRNAs, **(B)** miRNAs, and **(C)** mRNAs. **(D–F)** Volcano plots of differentially expressed **(D)** lncRNAs, **(E)** miRNAs, and **(F)** mRNAs with criteria of P < 0.05 and |Fold change| > 2. Filtered transcripts were shown in gray, non-significant transcripts in blue, and significant transcripts in red. The horizontal axis represented the log2 fold change, while the vertical axis represents -log10 (P value). **(G–I)** Heat maps showing expression profiles of **(G)** lncRNAs, **(H)** miRNAs, and **(I)** mRNAs. These heat maps were constructed based on expression values detected by RNA-seq. Expression values range from −2 to 2, with the color scale indicating intensity from green (low expression) to red (high expression). Each column represents a sample, and each row represents a transcript, with three osteogenic induced groups and three control groups included.

Based on the sequencing data, 576 lncRNAs were differentially expressed, with 269 upregulated and 307 downregulated in the treatment group compared to the control group (Figure 3A). Of these, MERGE.52938.29 and MERGE.38189.17 were the most up- and downregulated differentially expressed lncRNA, respectively (Table 1). Additionally, 13 upregulated and 20 downregulated miRNAs, totaling 33 miRNAs, also showed differential expression (Figure 3B). Among these, miR-122-5p was significantly downregulated (Table 2). Meanwhile, 1717 differentially expressed mRNAs were identified, including 777 upregulated and 940 downregulated mRNAs (Figure 3C). Of these, SLC35A3 and RIOK3 were the most up- and downregulated differentially expressed mRNA, respectively (Table 3). The volcano plot illustrated differentially expressed lncRNAs, miRNAs and mRNAs with statistical significance (P ≤ 0.05) and |fold change|≥2 (Figures 3D–F). The heat map also displayed the differences in lncRNAs, miRNAs and mRNAs expression between treatment group and control group (Figures 3G–I). The apparent difference in transcript levels between these two groups highlighted the crucial roles of lncRNAs, miRNAs and mRNAs in REG therapy for JPQHSYY.

**TABLE 1:** Top 10 Up- and Downregulated DE lncRNAs.

ID	logFC	P value	Regulation
MERGE.52938.29	13.4031	3.31E-05	up
MERGE.52938.5	12.63617	0.000244	up
NONHSAT160559.1	8.998341	2.21E-05	up
MERGE.48800.48	8.513523	3.4E-07	up
ENST00000647354	7.622446	0.010295	up
MERGE.19079.65	7.582059	0.004364	up
NONHSAT141042.2	7.343746	0.000335	up
MERGE.39451.21	6.963193	0.005522	up
MERGE.48851.16	6.73258	0.011875	up
MERGE.45812.1	6.617533	0.001658	up
MERGE.38189.17	−9.47094907130619	6.24E-09	down
MERGE.29321.53	−8.90891869804102	0.006236	down
MERGE.34829.3	−8.86461189553109	0.026255	down
MERGE.15875.3	−8.35768824157672	0.006817	down
MERGE.32719.47	−8.33586331793849	0.022326	down
MERGE.12627.1	−8.22407098683439	0.000529	down
MERGE.35361.3	−7.98036237243967	0.006122	down
MERGE.35477.3	−7.48032300718199	0.005948	down
MERGE.38199.11	−7.34558497307861	0.005848	down
MERGE.12029.3	−7.28784463467919	0.019131	down

**TABLE 2:** The Up- and Downregulated DE miRNAs.

ID	logFC	P value	Regulation
hsa-miR-3691-3p	4.584687	0.034903	up
hsa-miR-412-3p	3.718811	0.049181	up
hsa-miR-205-5p	3.375894	0.001275	up
hsa-miR-10397-3p	3.234433	0.029847	up
hsa-miR-2682-5p	2.929453	0.027166	up
hsa-miR-4999-5p	2.639896	0.042049	up
hsa-miR-363-5p	2.115598	0.045291	up
hsa-let-7c-3p	1.792677	0.043317	up
hsa-miR-3664-3p	1.724415	0.037316	up
hsa-miR-873-5p	1.610748	0.049103	up
hsa-miR-148a-3p	1.325045	0.015994	up
hsa-miR-143-3p	1.191982	0.003015	up
hsa-miR-99a-5p	1.014643	0.028935	up
hsa-miR-302a-5p	−7.50392	0.000154	down
hsa-miR-302a-3p	−4.65981	0.013512	down
hsa-miR-302d-3p	−4.30313	0.021814	down
hsa-miR-302b-3p	−4.26222	0.010004	down
hsa-miR-3199	−4.10424	0.016707	down
hsa-miR-302c-3p	−3.91866	0.043657	down
hsa-miR-4775	−3.91578	0.014465	down
hsa-miR-6820-3p	−3.60142	0.036622	down
hsa-miR-552-3p	−3.13254	0.008609	down
hsa-miR-4497	−2.99772	0.039246	down
hsa-miR-552-5p	−2.92235	0.045615	down
hsa-miR-943	−2.86175	0.025242	down
hsa-miR-122-5p	−1.80247	0.039172	down
hsa-miR-1293	−1.67644	0.044498	down
hsa-miR-1249-3p	−1.59457	0.032127	down
hsa-miR-509-3-5p	−1.47752	0.03926	down
hsa-miR-7974	−1.30436	0.002781	down
hsa-miR-548p	−1.28787	0.011551	down
hsa-miR-548ah-3p	−1.26271	0.012837	down
hsa-miR-451a	−1.14103	0.011773	down

**TABLE 3:** Top 10 up- and downregulated DE mRNAs.

ID	Symbol	logFC	P value	Regulation
ENST00000640360	SLC35A3	10.34627	0.000216	up
ENST00000587994	ACAA2	9.385537	0.000276	up
ENST00000425278	KLHL8	8.207475	0.000582	up
ENST00000601091	LIG1	8.178844	0.001312	up
ENST00000517540	TRIQK	7.583954	0.003927	up
ENST00000645412	POLR1D	7.554465	0.031614	up
ENST00000613264	ST3GAL6	7.510992	0.004289	up
ENST00000563158	DNAJA2	7.44416	0.003193	up
ENST00000371544	ZCCHC11	7.351221	0.000685	up
ENST00000319826	RPL21	7.291173	0.002	up
ENST00000584130	RIOK3	−1.54427476175081	0.030296	down
ENST00000292614	POLR2J	−1.5073704174403	0.022348	down
ENST00000359273	BCS1L	−5.16723430695944	0.01377	down
ENST00000488392	C21orf33	−1.31996522028618	0.04787	down
ENST00000587821	SEC14L1	−1.29985228534068	0.03132	down
ENST00000511257	CCDC125	−2.49102279286635	0.048275	down
ENST00000452421	TMEM59	−3.49141991237638	0.00105	down
ENST00000496875	EIF3D	−1.08758385113789	0.049949	down
ENST00000398558	MFSD12	−3.98081253820234	0.006521	down
ENST00000256578	AMPD2	−5.65645331389444	0.010144	down

### 3.4 Functional Prediction of Interactive miRNAs

**FIGURE 4:**
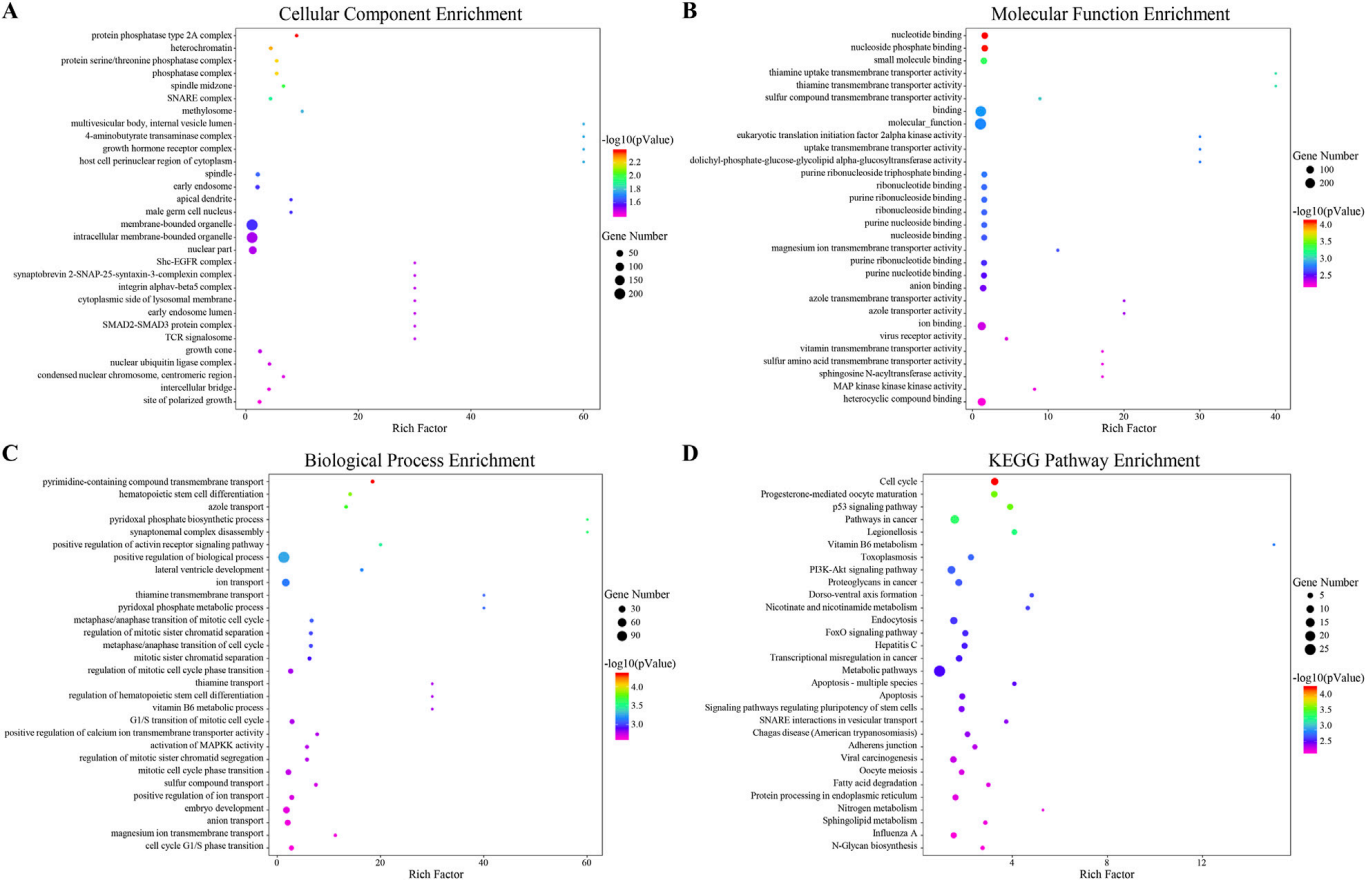
Functional Prediction of Interactive miRNAs. **(A–C)** The top 30 enriched Gene Ontology (GO) terms for each category: **(A)** biological process, **(B)** cellular component, and **(C)** molecular function. These terms are ranked by -log10 (P value) based on GO analysis.** (D)** The top 30 enriched pathways from the Kyoto Encyclopedia of Genes and Genomes (KEGG), ranked by enrichment score.

The differentially expressed 33 miRNAs were further selected for GO and KEGG enrichment analysis to evaluate their roles in biological processes, cellular components, molecular functions, and pathways. In total, 653 GO terms were analyzed, including 460 terms enriched in biological process, 43 terms enriched in cellular component, and 150 terms enriched in molecular function. The significant enrichment bubble plots showed the top 30 items for each category ranked by enrichment score. The most significantly enriched GO term in the cellular component was “protein phosphatase type 2A complex”, while “nucleotide binding” was the most significantly enriched GO term in molecular function (Figures 4A, B). Furthermore, biological processes such as “pyrimidine-containing compound transmembrane transport, hematopoietic stem cell differentiation, azole transport, synaptonemal complex disassembly, pyridoxal phosphate biosynthetic process, positive regulation of activin receptor signaling pathway, positive regulation of biological process, lateral ventricle development, ion transport, pyridoxal phosphate metabolic process, metaphase/anaphase transition of mitotic cell cycle, G1/S transition of mitotic cell cycle” were also enriched (Figure 4C).

By KEGG pathways analysis, 41 significant pathways were detected and ranked by enrichment score with the top 30 pathways listed (Figure 4D). Among these, cell cycle and apoptosis, as well as pathways including the p53 signaling pathway, PI3K-AKT signaling pathway, and FoxO signaling pathway were enriched. Both GO and KEGG enrichment analyses indicated that cell cycle, apoptosis, and PI3K-AKT signaling pathway might be the potential regulatory mechanisms of JPQHSYY treatment against REG.

### 3.5 miRNA-lncRNA network

**FIGURE 5:**
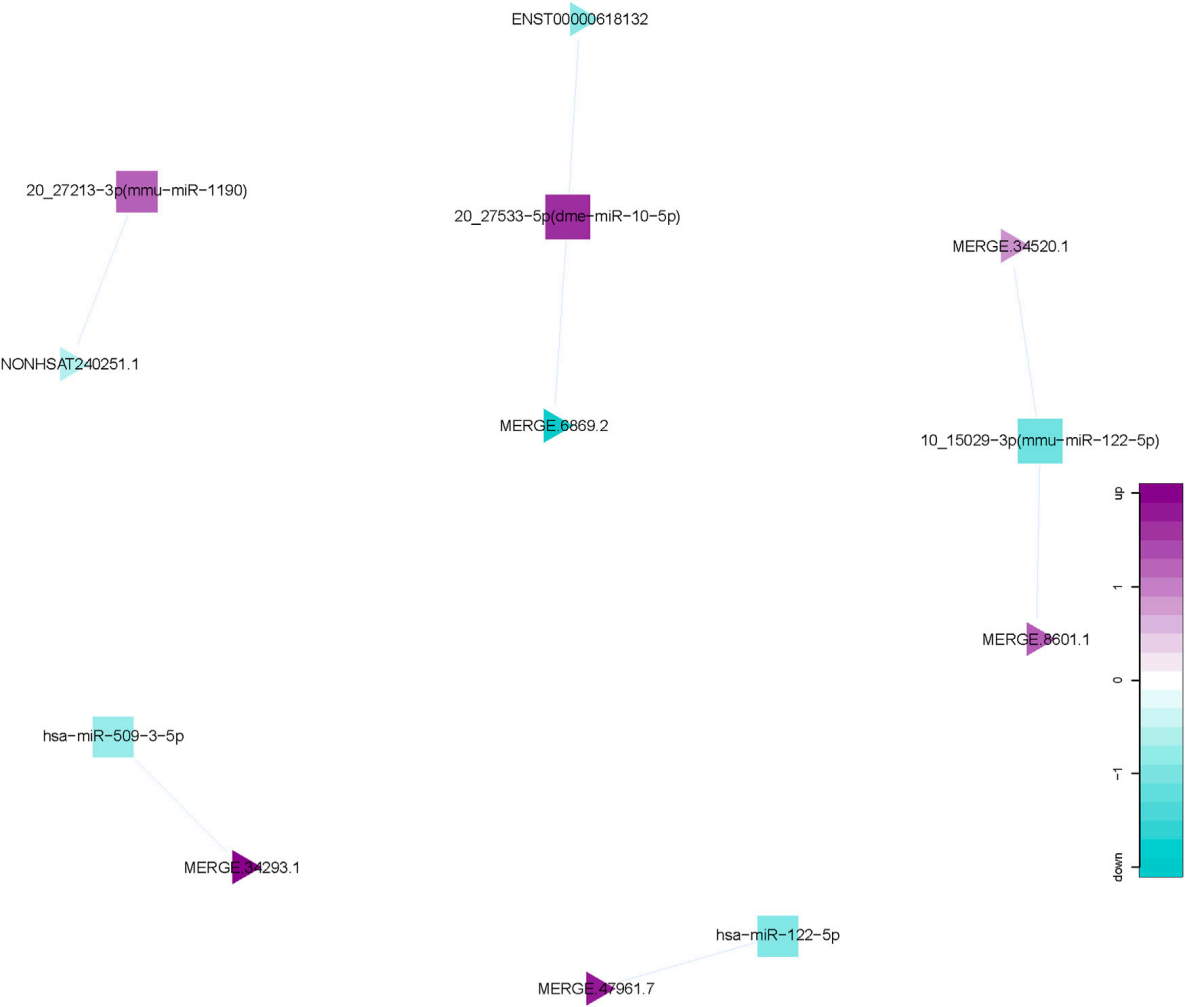
miRNA-lncRNA Network. Interaction network of competing endogenous RNA (ceRNA) involving miRNA and lncRNA. Triangular nodes represent lncRNAs, and square nodes represent miRNAs.

We calculated the expression correlation coefficient between differentially expressed miRNAs and differentially expressed lncRNAs, and screened for significantly negative correlation pairs. miRNA-target genes were predicted using the miRanda software and this information was applied to develop the miRNA-lncRNA network using the Cytoscape software. This network comprised 12 nodes (5 differentially expressed miRNAs; 7 differentially expressed lncRNAs), and 7 miRNA-lncRNA interactions (Figure 5). There is a negative correlation between lncRNA and miRNA based on the ceRNA hypothesis. Thus, the regulatory relationship between key miRNAs and key lncRNAs were further analyzed. Interestingly, miR-122-5p, one of the 5 key miRNAs mentioned above, were observed and functioned as the regulators of lncRNA expression.

### 3.6 Effect of JPQHSYY on the PI3K/AKT pathway in gastric mucosal tissues of REG patients

**FIGURE 6:**
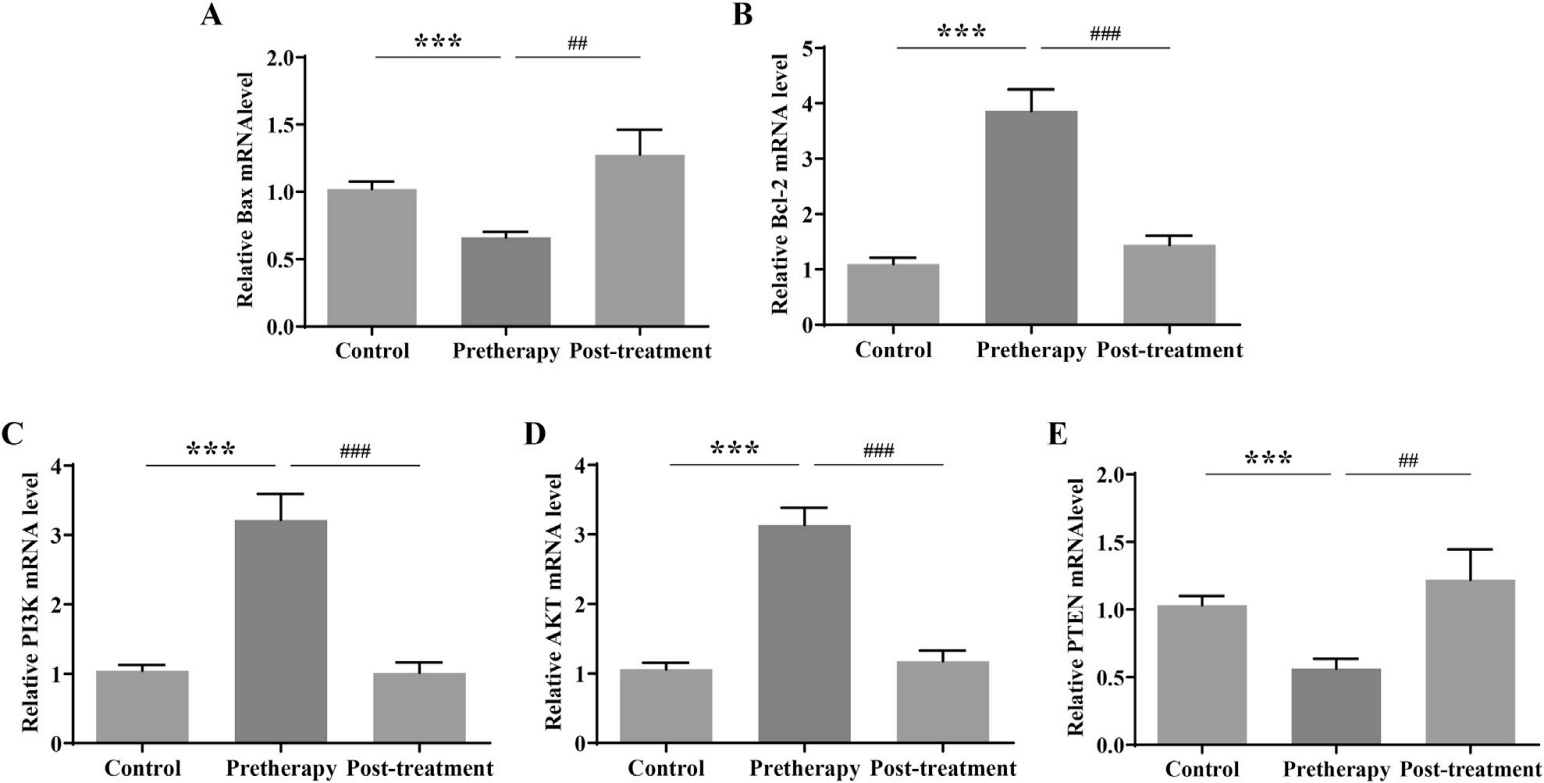
Effect of Jianpi Qinghua Sanyu Yin (JPQHSYY) on the PI3K/AKT Pathway in Gastric Mucosal Tissues of REG Patients. Quantitative real-time PCR (qRT-PCR) was used to measure the relative mRNA expression levels of **(A)** Bax, **(B)** Bcl-2, **(C)** p-PI3K, **(D)** p-AKT and **(E)** PTEN in gastric mucosal tissues of REG patients. *P < 0.05 **P < 0.01 **P < 0.001 compared to the Control group, ^#^P < 0.05 ^##^P < 0.01 ^###^P < 0.001 compared to the Pretherapy group. All values are mean ± standard deviation (SD).

Based on GO and KEGG enrichment and miRNA-lncRNA network analysis, Q-PCR were first used to detect the expression of genes related to the PI3K/AKT signaling pathway in gastric mucosal tissues of normal subjects and REG patients before and after treatment. The results of QPCR showed that compared with the normal group, the mRNA expression of Bcl2, PI3K and AKT in the gastric mucosa of REG patients before treatment was significantly increased, while the expression of Bax and PTEN was significantly decreased; After treatment with JPQHSYY, the mRNA expression of Bcl2, PI3K and AKT in the gastric mucosa was significantly decreased, and the mRNA expression of Bax and PTEN was significantly increased (Figure 6).

### 3.7 Effects of JPQHSYY on proliferation and apoptosis in GES-1 cells-transfected with miR-122-5p mimic

**FIGURE 7:**
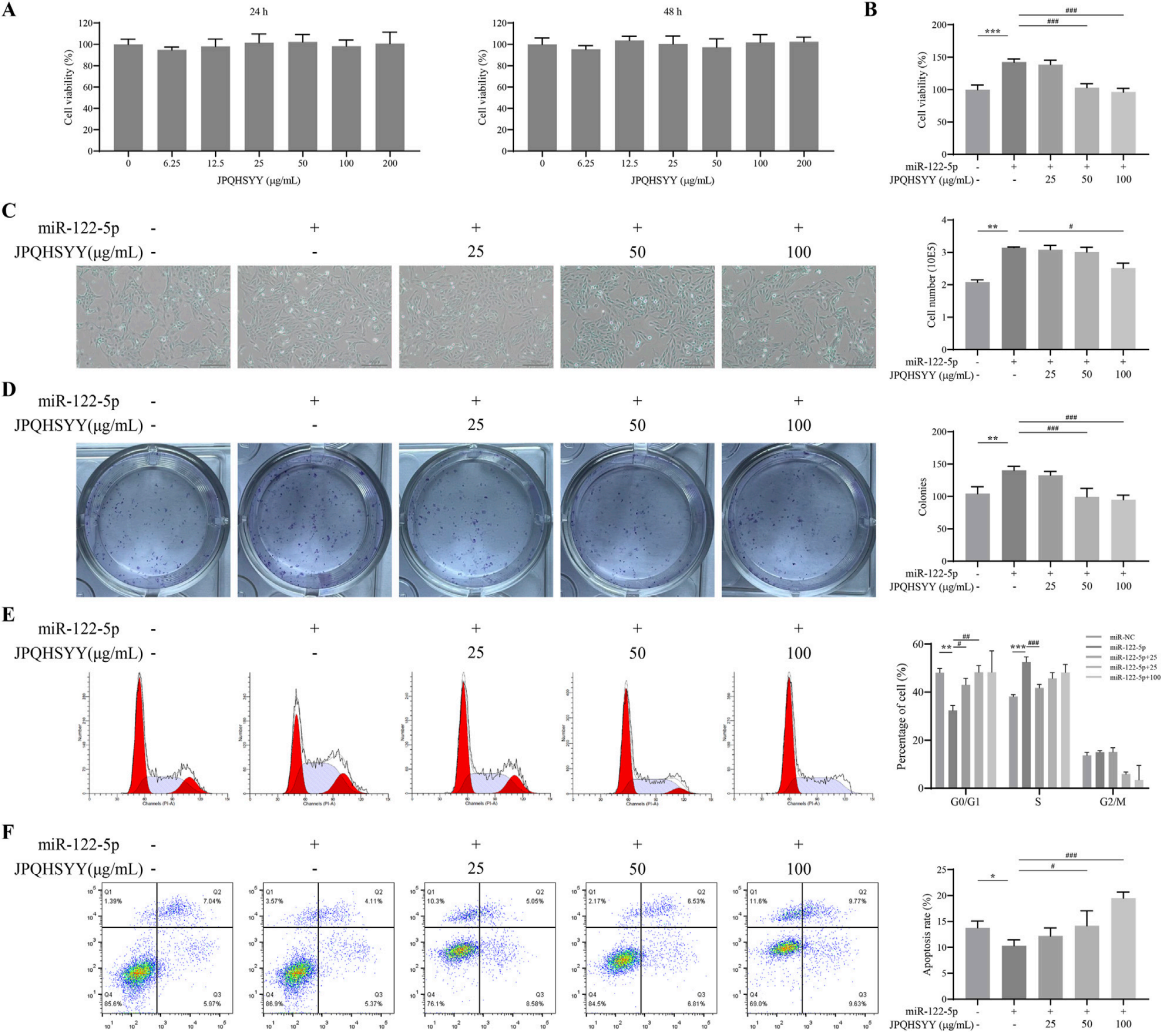
Effects of Jianpi Qinghua Sanyu Yin (JPQHSYY) on Proliferation and Apoptosis in GES-1 Cells transfected with miR-122-5p mimic. **(A)** Viability of GES-1 cells after treatment with JPQHSYY for 24 and 48 h. Viability of untreated GES-1 cells was set as 100%. **(B)** Viability of GES-1 cells induced by miR122-5p mimic transfection after treatment with JPQHSYY. Viability of GES-1 cells transfected with miR-NC was set as 100%. **(C)** Representative images and cell number counts of growth of GES-1 cells induced by miR-122-5p mimic transfection after treatment with JPQHSYY. **(D)** Representative images of cell colony formation and cell survival capacity of GES-1 cells induced by miR-122-5p mimic transfection after treatment with JPQHSYY. **(E)** Representative images of cell cycle analysis and statistics of GES-1 cells induced by miR-122-5p mimic transfection after treatment with JPQHSYY. **(F)** Representative images of cell apoptosis analysis and apoptosis rate statistics of GES-1 cells induced by miR-122-5p mimic transfection after treatment with JPQHSYY. *P < 0.05 **P < 0.01 **P < 0.001 compared to the miR-NC group, ^#^P < 0.05 ^##^P < 0.01 ^###^P < 0.001 compared to the miR-122-5p mimic group. All values are mean ± standard deviation (SD).

Since miR-122-5p was found to be downregulated from RNA-sequencing and is considered as a potential new biomarker for early detection of gastritis and gastric cancer, we used GES-1 cells transfected with miR-122-5p mimic to induce abnormal proliferation. Firstly, we evaluated the effect of JPQHSYY on the cell viability of GES-1 cells by CCK8 assay. As shown in Figure 7A, different concentrations of JPQHSYY (6.25–200 μg/mL) had no significant effect on cell viability. Therefore, concentrations of JPQHSYY at 25, 50, and 100 μg/mL were selected for subsequent experiments.

CCK8 assay results indicated that miR-122-5p mimic transfection markedly increased GES-1 cell viability, which was significantly suppressed by JPQHSYY treatment (Figure 7B). Microscopic observation of cell growth showed that miR-122-5p mimic transfection significantly increased GES-1 cell number, while JPQHSYY treatment significantly inhibited this increase (Figure 7C). Colony formation assay results showed that miR-122-5p mimic transfection significantly increased the number of colonies formed in GES-1 cells compared to miR-NC transfection, while JPQHSYY treatment significantly inhibited this increase (Figure 7D).

Flow cytometry analysis of the cell cycle revealed that miR-122-5p mimic transfection significantly reduced the proportion of GES-1 cells in the G0/G1 phase and increased the proportions in the S and G2/M phases compared to miR-NC transfection. JPQHSYY treatment significantly suppressed the reduction in the G0/G1 phase and the increase in the S and G2/M phases induced by miR-122-5p transfection, leading to cell cycle arrest in the G0/G1 phase and inhibition of further cell proliferation (Figure 7E). These results suggest that JPQHSYY can inhibit abnormal proliferation of GES-1 cells by suppressing miR-122-5p mimic transfection-induced increases in cell viability and colony formation, and cell cycle arrest in the G0/G1 phase.

Flow cytometry analysis was performed to further investigate the effect of JPQHSYY on cell apoptosis in GES-1 cells transfected with miR-122-5p mimic. Compared to miR-NC transfection, miR-122-5p mimic transfection significantly reduced the number of apoptotic GES-1 cells, while JPQHSYY treatment significantly inhibited the reduction of apoptotic cells-induced by miR-122-5p transfection (Figure 7F). These results indicate that JPQHSYY can counteract miR-122-5p-induced inhibition of cell apoptosis.

### 3.8 Effects of JPQHSYY on expression of proliferation and apoptosis-related proteins, and the PI3K/AKT pathway in GES-1 cells-transfected with miR-122-5p mimic

**FIGURE 8:**
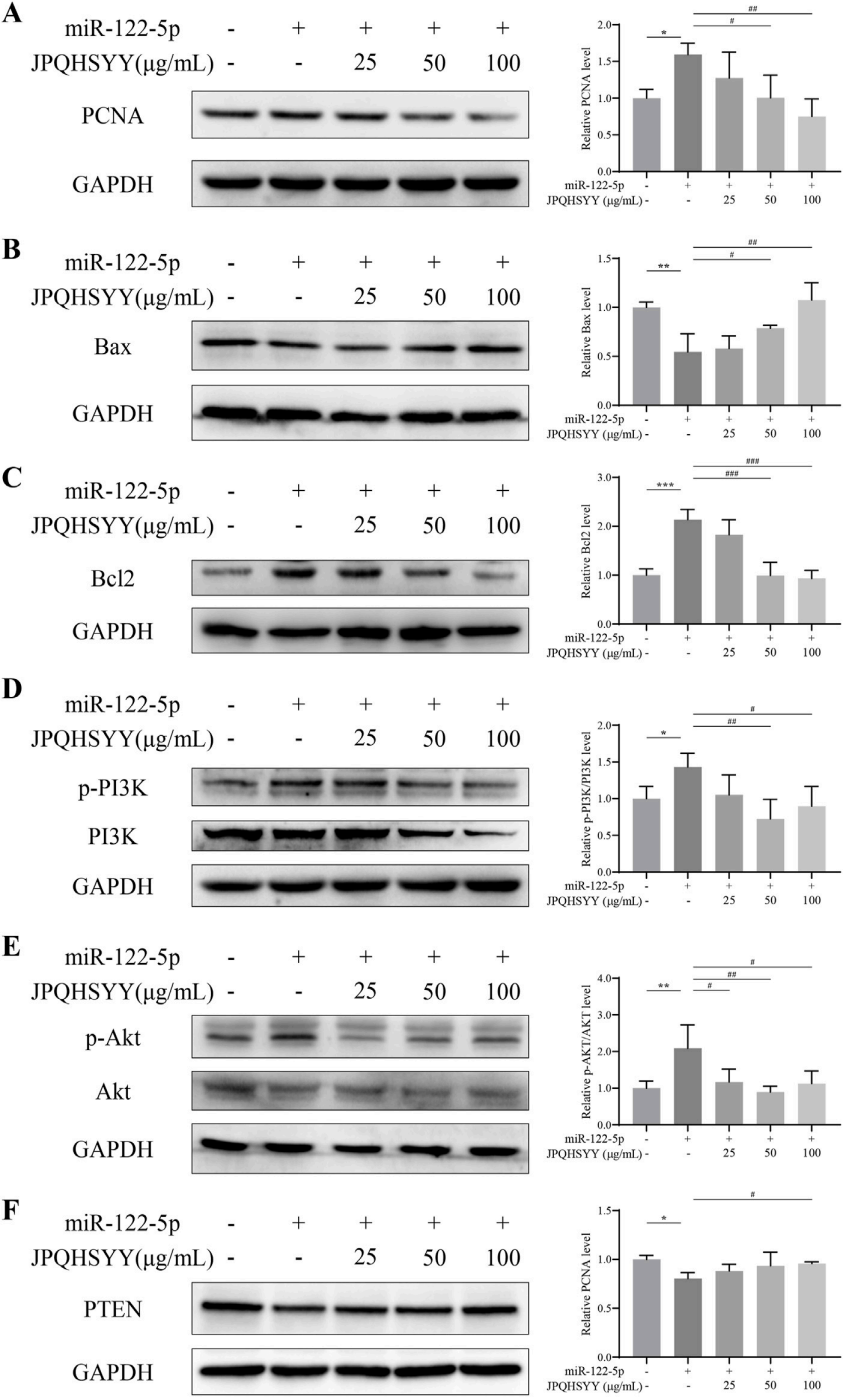
Effects of Jianpi Qinghua Sanyu Yin (JPQHSYY) on Proliferation and Apoptosis-Related Proteins, and the PI3K/AKT Pathway in GES-1 Cells transfected with miR-122-5p mimic. Western blot analysis was used to measure the relative protein expression levels of **(A)** PCNA, **(B)** Bax, **(C)** Bcl-2, **(D)** p-PI3K, PI3K, **(E)** p-AKT, AKT and **(F)** PTEN in GES-1 cells transfected with miR-122-5p mimic and treated with JPQHSYY. *P < 0.05 **P < 0.01 **P < 0.001 compared to the miR-NC group, ^#^P < 0.05 ^##^P < 0.01 ^###^P < 0.001 compared to the miR-122-5p mimic group. All values are mean ± standard deviation (SD).

Western blot analysis was performed to detect proliferation and apoptosis-related protein expression. It showed that miR-122-5p mimic transfection upregulated PCNA and Bcl-2 expression in GES-1 cells, and downregulated Bax expression compared to miR-NC transfection. JPQHSYY treatment significantly suppressed the upregulation of PCNA and Bcl-2 and the downregulation of Bax induced by miR-122-5p mimic transfection (Figures 8A–C). These results indicate that JPQHSYY inhibit GES-1 cell proliferation and promote apoptosis by downregulating proliferation-related proteins and anti-apoptotic protein Bcl-2, and upregulating pro-apoptotic protein Bax.

To further explore the molecular mechanisms by which JPQHSYY inhibits cell proliferation and promotes apoptosis, western blot analysis was conducted to detect PI3K/AKT pathway-related protein expression. Compared to miR-NC transfection, miR-122-5p mimic transfection upregulated p-PI3K and p-AKT and downregulated PTEN expression in GES-1 cells. JPQHSYY treatment significantly decreased the expression of p-PI3K and p-AKT induced by miR-122-5p mimic transfection and increased the expression of PTEN (Figures 8D–F). These results suggest that JPQHSYY inhibits miR-122-5p-induced abnormal proliferation and promotes apoptosis in GES-1 cells by inhibiting the activation of the PI3K/AKT pathway.

## 4 Discussion

REG is a special type of gastritis characterized by local cell proliferation and has a high propensity for malignant transformation (Xiao et al., 2022; Hirano et al., 2022). Under physiological conditions, there is a dynamic balance between cell proliferation and apoptosis in normal gastric mucosal tissue. Disruption of this balance leads to pathological changes, including epithelial and gastric pit hyperplasia, gland neck elongation, and fibrous tissue proliferation. These features, along with excessive proliferation of the muscularis mucosae lea, contribute to the characteristic raised lesions observed in REG. Compared to chronic atrophic gastritis, REG exhibits a higher likelihood of malignant transformation, potentially advancing to gastric cancer if untreated (Vieth and Stolte, 2006). Multiple signaling pathways are involved in the regulation of cell proliferation and apoptosis, emphasizing the need for targeted therapeutic strategies.

This study provides a comprehensive molecular analysis of the therapeutic effect of JPQHSYY on REG. Using RNA-seq, we systematically profiled mRNA, lncRNA, and miRNA expression in REG patients before and after JPQHSYYY treatment. A total of 576 lncRNAs, 33 miRNAs, and 1717 mRNAs were identified as differentially expressed, highlighting the crucial roles of these transcripts in mediating JPQHSYY’s therapeutic effects.

Functional enrichment analysis indicated that differentially expressed genes were enriched in progresses related to cell cycle regulation, apoptosis, and signaling pathways such as p53 pathway, PI3K-AKT pathway. Both lncRNAs and protein-coding mRNAs were found to function as ceRNAs, acting as molecular sponges to regulate miRNA expression. To explore these interactions, we applied bioinformatic tools to predict miRNA–mRNA and miRNA–lncRNA interactions and consructed triple regulatory networks among lncRNA–miRNA–mRNA in REG patients receiving JPQHSYY therapy. Within ceRNA network, the PI3K/AKT signaling pathway emerged as a key axis, further emphasizing its central role in mediating the therapeutic effects of JPQHSYY.

The PI3K/AKT pathway is a classic anti-apoptotic signaling pathway involved in regulating cell proliferation, survival, and migration (Khezri et al., 2022). Dysregulation of this pathway has been implicated in the pathogenesis of REG, as evidenced by elevated expression of basic fibroblast growth factor (bFGF) and its receptor, anti-apoptotic factor Bcl-2, proliferating cell nuclear antigen (PCNA), and epidermal growth factor (EGF), alongside reduced expression of the pro-apoptotic factor Bax (Fang et al., 2017; Mao et al., 2019). Therefore, finding drugs that inhibit abnormal activation of the PI3K/AKT pathway is the key to preventing and treating REG. As expected, JPQHSYY treatment significantly inhibited the PI3K/AKT pathway, reduced cell proliferation, and promoted apoptosis, as confirmed by qPCR and western blot analysis. This highlights the potential of JPQHSYY as a therapeutic agent targeting aberrant REG signaling.

Numerous studies have shown that multiple miRNAs are involved in cell proliferation mediated by abnormal activation of the PI3K/AKT pathway (Wang et al., 2018; Akbarzadeh et al., 2021). miR-19a can inhibit PTEN expression, thereby promoting cell proliferation through the PI3K/AKT signaling pathway (Zhao et al., 2019). miR-21 inhibitors can suppress cell proliferation, migration, and invasion by upregulating PTEN expression and inhibiting the PI3K/AKT pathway, suggesting that miR-21 may regulate cell proliferation through the PI3K/AKT pathway (Shi et al., 2017). Based on RNA sequencing, a total of 33 differentially expressed miRNAs were identified, with 20 upregulated and 13 downregulated. Among these miRNAs, miR-122-5p, a key miRNA identified in this study, was found to be highly expressed in the gastric tissues of REG patients before treatment and significantly downregulated following JPQHSYY treatment. It has been reported that miR-122-5p promotes the proliferation, invasion, and migration of cancer cells through the PI3K/AKT signaling pathway (Li et al., 2020). Additionally, it is considered a potential new biomarker for the early detection of gastritis and gastric cancer (You et al., 2021; Liu et al., 2021; Zhu et al., 2019). These imply that miR-122-5p might be involved in the development of REG via the regulation of PI3K/AKT pathway and could serve as a potential therapeutic target.

Given that miR-122-5p can lead to abnormal proliferation and inhibited apoptosis of GES-1 cells, this study first used miR-122-5p mimic transfection in GES-1 cells to verify the effects of JPQHSYY on miR-122-5p-induced proliferation and apoptosis *in vitro*. The results confirmed that JPQHSYY treatment could reduce cell viability and colony formation, arrest the cell cycle at the G0/G1 phase, and promote apoptosis in miR-122-5p transfection-induced GES-1 cells. Further western blot analysis also found that JPQHSYY could reverse the upregulation the expression of PCNA and Bcl-2 and the downregulation the expression of Bax induced by miR-122-5p transfection. These results indicate that JPQHSYY treatment can reverse miR-122-5p-induced abnormal proliferation and apoptosis inhibition by regulating proliferation and apoptosis-related proteins. KEGG enrichment analysis of RNA sequencing results revealed that the PI3K/AKT pathway was highly enriched, suggesting that miRNAs might be involved in the development of REG and could be a crucial pathway for REG treatment. Therefore, we further examined the effect of JPQHSYY on the PI3K/AKT pathway in GES-1 cells after miR-122-5p transfection. The results confirmed that JPQHSYY could inhibit the activation of the PI3K/AKT pathway. These findings suggest that JPQHSYY can inhibit the abnormal proliferation of GES-1 cells by suppressing the activation of the miRNAs/PI3K/AKT signaling pathway and regulating the expression of downstream related factors.

Although our findings highlighted the ceRNA network and PI3K/AKT pathway as central components of the molecular mechanisms underlying JPQHSYY’s therapeutic effects, this study has several limitations. First, the sample size for RNA-seq analysis was relatively small, limiting, the generalizability of our results. Future studies will expand sample size to validate these findings. Additionally, the direct mechanistic interplay between the compound and these pathways was not fully elaborated. The complexity of JPQHSYY, a multi-herb formulation, presents challenges in delineating the contribution of individual components. Future studies will incorporate network pharmacology and experimental validation to identify and confirm the roles of key active compounds. Moreover, targeted experimental approaches, such as pathway-specific inhibitors and gene knockdown animal models, will be employed to further elucidate the direct mecnistic interplay between JPQHSYY and identified molecular targets.

In conclusion, this study preliminarily demonstrated that JPQHSYY exerts a therapeutic effect on REG by regulating the miRNAs/PI3K/AKT signaling pathway and restoring cellular homeostasis. The findings not only enrich the understanding of JPQHSYY’s molecular mechanisms in treating REG but also provide further theoretical and experimental evidence supporting its clinical application. Future studies will focus on validating these findings in larger cohorts and further characterizing the ceRNA network and molecular pathways involved in the therapeutic effects of JPQHSYY, thereby advancing its potential as a treatment for REG.

## Data Availability

The RNA sequencing data presented in the study are deposited at NCBI Gene Expression Omnibus (GEO), accession number GSE289593 and GSE289594.
