# Supplementary material for: Mechanistic insights into Jianpi Qinghua Sanyu Yin treatment of raised erosive gastritis: ceRNA-mediated PI3K/AKT signaling pathways
Source: Front Pharmacol. 2025 Mar 3;16:1495020. doi: 10.3389/fphar.2025.1495020 (PMC11911336; doi:10.3389/fphar.2025.1495020)
Supplement: Supplementary file 2 [file DataSheet4.zip › E/Cell cycle.docx]

File analyzed: 20230719 LGS_Tube_001.fcs

Date analyzed: 6-Aug-2023

Model: 1nn0n_DSD

Analysis type: Manual analysis

Ploidy Mode: First cycle is diploid

Diploid: 100.00 %

Dip G1: 49.19 % at 52.86

Dip G2: 12.58 % at 105.71

Dip S: 38.23 % G2/G1: 2.00

%CV: 7.62

Total S-Phase: 38.23 %

Total B.A.D.: 0.00 % no debris no aggs

Debris: %

Aggregates: 0.00 %

Modeled events: 7432

All cycle events: 7432

Cycle events per channel: 138

RCS: 0.821

File analyzed: 20230719 LGS_Tube_002.fcs

Date analyzed: 6-Aug-2023

Model: 1nn0n_DSD

Analysis type: Manual analysis

Ploidy Mode: First cycle is diploid

Diploid: 100.00 %

Dip G1: 49.02 % at 54.18

Dip G2: 13.68 % at 108.36

Dip S: 37.31 % G2/G1: 2.00

%CV: 7.59

Total S-Phase: 37.31 %

Total B.A.D.: 0.00 % no debris no aggs

Debris: %

Aggregates: 0.00 %

Modeled events: 7091

All cycle events: 7091

Cycle events per channel: 129

RCS: 1.124

File analyzed: 20230719 LGS_Tube_003.fcs

Date analyzed: 6-Aug-2023

Model: 1nn0n_DSD

Analysis type: Manual analysis

Ploidy Mode: First cycle is diploid

Diploid: 100.00 %

Dip G1: 46.09 % at 55.04

Dip G2: 14.98 % at 110.07

Dip S: 38.93 % G2/G1: 2.00

%CV: 7.31

Total S-Phase: 38.93 %

Total B.A.D.: 0.00 % no debris no aggs

Debris: %

Aggregates: 0.00 %

Modeled events: 6308

All cycle events: 6308

Cycle events per channel: 113

RCS: 1.316

File analyzed: 20230719 LGS_Tube_004.fcs

Date analyzed: 6-Aug-2023

Model: 1nn0n_DSD

Analysis type: Manual analysis

Ploidy Mode: First cycle is diploid

Diploid: 100.00 %

Dip G1: 32.89 % at 48.31

Dip G2: 14.29 % at 96.62

Dip S: 52.82 % G2/G1: 2.00

%CV: 8.39

Total S-Phase: 52.82 %

Total B.A.D.: 0.00 % no debris no aggs

Debris: %

Aggregates: 0.00 %

Modeled events: 7260

All cycle events: 7260

Cycle events per channel: 147

RCS: 2.041

File analyzed: 20230719 LGS_Tube_005.fcs

Date analyzed: 6-Aug-2023

Model: 1nn0n_DSD

Analysis type: Manual analysis

Ploidy Mode: First cycle is diploid

Diploid: 100.00 %

Dip G1: 30.09 % at 50.38

Dip G2: 15.36 % at 100.75

Dip S: 54.55 % G2/G1: 2.00

%CV: 8.50

Total S-Phase: 54.55 %

Total B.A.D.: 0.00 % no debris no aggs

Debris: %

Aggregates: 0.00 %

Modeled events: 7063

All cycle events: 7063

Cycle events per channel: 137

RCS: 1.220

File analyzed: 20230719 LGS_Tube_006.fcs

Date analyzed: 6-Aug-2023

Model: 1nn0n_DSD

Analysis type: Manual analysis

Ploidy Mode: First cycle is diploid

Diploid: 100.00 %

Dip G1: 34.12 % at 51.20

Dip G2: 15.48 % at 102.41

Dip S: 50.39 % G2/G1: 2.00

%CV: 8.68

Total S-Phase: 50.39 %

Total B.A.D.: 0.00 % no debris no aggs

Debris: %

Aggregates: 0.00 %

Modeled events: 5855

All cycle events: 5855

Cycle events per channel: 112

RCS: 0.831

File analyzed: 20230719 LGS_Tube_007.fcs

Date analyzed: 6-Aug-2023

Model: 1nn0n_DSD

Analysis type: Manual analysis

Ploidy Mode: First cycle is diploid

Diploid: 100.00 %

Dip G1: 43.44 % at 55.53

Dip G2: 13.99 % at 111.07

Dip S: 42.56 % G2/G1: 2.00

%CV: 7.16

Total S-Phase: 42.56 %

Total B.A.D.: 0.00 % no debris no aggs

Debris: %

Aggregates: 0.00 %

Modeled events: 6355

All cycle events: 6355

Cycle events per channel: 112

RCS: 1.766

File analyzed: 20230719 LGS_Tube_008.fcs

Date analyzed: 6-Aug-2023

Model: 1nn0n_DSD

Analysis type: Manual analysis

Ploidy Mode: First cycle is diploid

Diploid: 100.00 %

Dip G1: 40.25 % at 56.22

Dip G2: 17.13 % at 112.44

Dip S: 42.62 % G2/G1: 2.00

%CV: 6.52

Total S-Phase: 42.62 %

Total B.A.D.: 0.00 % no debris no aggs

Debris: %

Aggregates: 0.00 %

Modeled events: 5970

All cycle events: 5970

Cycle events per channel: 104

RCS: 2.142

File analyzed: 20230719 LGS_Tube_009.fcs

Date analyzed: 6-Aug-2023

Model: 1nn0n_DSD

Analysis type: Manual analysis

Ploidy Mode: First cycle is diploid

Diploid: 100.00 %

Dip G1: 45.50 % at 56.35

Dip G2: 14.31 % at 112.70

Dip S: 40.19 % G2/G1: 2.00

%CV: 6.69

Total S-Phase: 40.19 %

Total B.A.D.: 0.00 % no debris no aggs

Debris: %

Aggregates: 0.00 %

Modeled events: 6281

All cycle events: 6281

Cycle events per channel: 110

RCS: 2.212

File analyzed: 20230719 LGS_Tube_010.fcs

Date analyzed: 6-Aug-2023

Model: 1nn0n_DSD

Analysis type: Manual analysis

Ploidy Mode: First cycle is diploid

Diploid: 100.00 %

Dip G1: 51.42 % at 57.48

Dip G2: 5.57 % at 114.95

Dip S: 43.01 % G2/G1: 2.00

%CV: 6.59

Total S-Phase: 43.01 %

Total B.A.D.: 0.00 % no debris no aggs

Debris: %

Aggregates: 0.00 %

Modeled events: 7785

All cycle events: 7785

Cycle events per channel: 133

RCS: 3.436

File analyzed: 20230719 LGS_Tube_011.fcs

Date analyzed: 6-Aug-2023

Model: 1nn0n_DSD

Analysis type: Manual analysis

Ploidy Mode: First cycle is diploid

Diploid: 100.00 %

Dip G1: 47.50 % at 58.15

Dip G2: 5.28 % at 116.30

Dip S: 47.22 % G2/G1: 2.00

%CV: 6.32

Total S-Phase: 47.22 %

Total B.A.D.: 0.00 % no debris no aggs

Debris: %

Aggregates: 0.00 %

Modeled events: 6521

All cycle events: 6521

Cycle events per channel: 110

RCS: 4.006

File analyzed: 20230719 LGS_Tube_012.fcs

Date analyzed: 6-Aug-2023

Model: 1nn0n_DSD

Analysis type: Manual analysis

Ploidy Mode: First cycle is diploid

Diploid: 100.00 %

Dip G1: 46.12 % at 57.85

Dip G2: 6.91 % at 115.70

Dip S: 46.98 % G2/G1: 2.00

%CV: 6.03

Total S-Phase: 46.98 %

Total B.A.D.: 0.00 % no debris no aggs

Debris: %

Aggregates: 0.00 %

Modeled events: 6470

All cycle events: 6470

Cycle events per channel: 110

RCS: 4.021

File analyzed: 20230719 LGS_Tube_013.fcs

Date analyzed: 6-Aug-2023

Model: 1nn0n_DSD

Analysis type: Manual analysis

Ploidy Mode: First cycle is diploid

Diploid: 100.00 %

Dip G1: 55.28 % at 59.29

Dip G2: 0.00 % at 118.59

Dip S: 44.72 % G2/G1: 2.00

%CV: 6.69

Total S-Phase: 44.72 %

Total B.A.D.: 0.00 % no debris no aggs

Debris: %

Aggregates: 0.00 %

Modeled events: 7846

All cycle events: 7846

Cycle events per channel: 130

RCS: 4.356

File analyzed: 20230719 LGS_Tube_014.fcs

Date analyzed: 6-Aug-2023

Model: 1nn0n_DSD

Analysis type: Manual analysis

Ploidy Mode: First cycle is diploid

Diploid: 100.00 %

Dip G1: 38.34 % at 58.64

Dip G2: 10.39 % at 117.28

Dip S: 51.27 % G2/G1: 2.00

%CV: 5.24

Total S-Phase: 51.27 %

Total B.A.D.: 0.00 % no debris no aggs

Debris: %

Aggregates: 0.00 %

Modeled events: 5408

All cycle events: 5408

Cycle events per channel: 91

RCS: 5.043

File analyzed: 20230719 LGS_Tube_015.fcs

Date analyzed: 6-Aug-2023

Model: 1nn0n_DSD

Analysis type: Manual analysis

Ploidy Mode: First cycle is diploid

Diploid: 100.00 %

Dip G1: 51.30 % at 59.82

Dip G2: 0.00 % at 119.64

Dip S: 48.70 % G2/G1: 2.00

%CV: 6.43

Total S-Phase: 48.70 %

Total B.A.D.: 0.00 % no debris no aggs

Debris: %

Aggregates: 0.00 %

Modeled events: 6301

All cycle events: 6301

Cycle events per channel: 104

RCS: 4.814
