# Supplementary figures and images for: Mechanistic insights into Jianpi Qinghua Sanyu Yin treatment of raised erosive gastritis: ceRNA-mediated PI3K/AKT signaling pathways
Source: Front Pharmacol. 2025 Mar 3;16:1495020. doi: 10.3389/fphar.2025.1495020 (PMC11911336; doi:10.3389/fphar.2025.1495020)

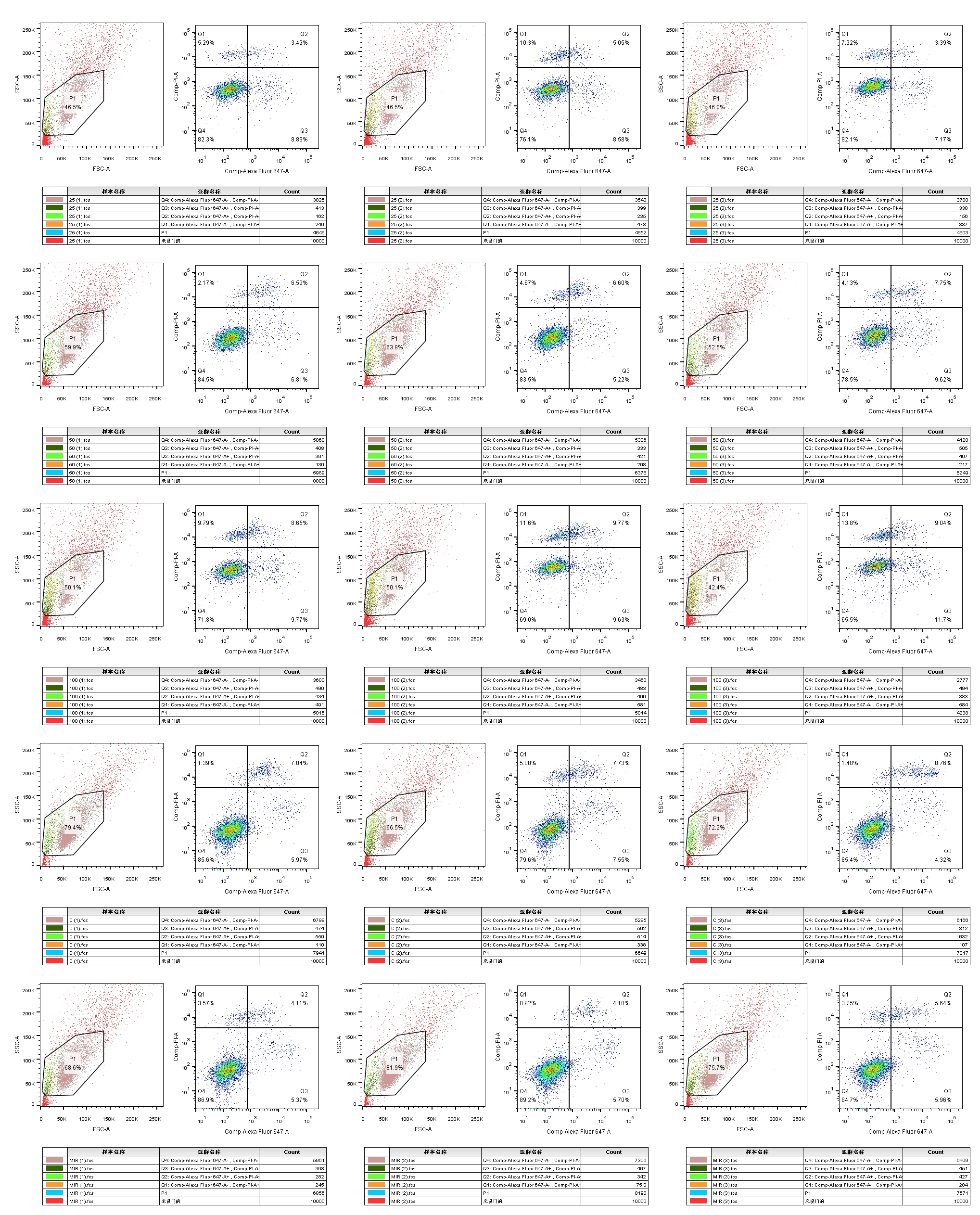

Supplement: Supplementary file 2 [file DataSheet4.zip › F/GES1.jpg]

## Slide 1
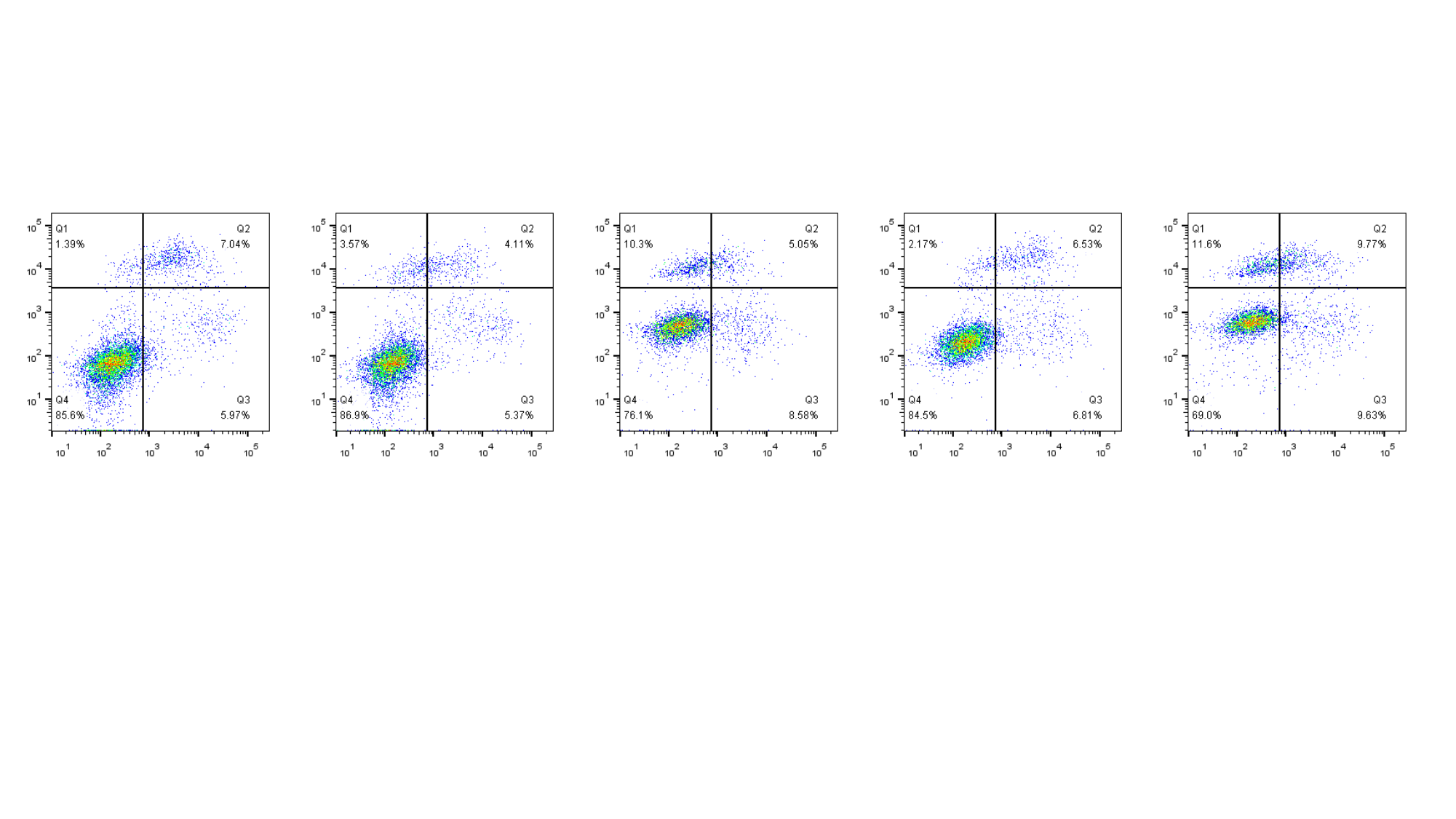

Supplement: Supplementary file 2 [file DataSheet4.zip › F/图片.pptx]

## Slide 1
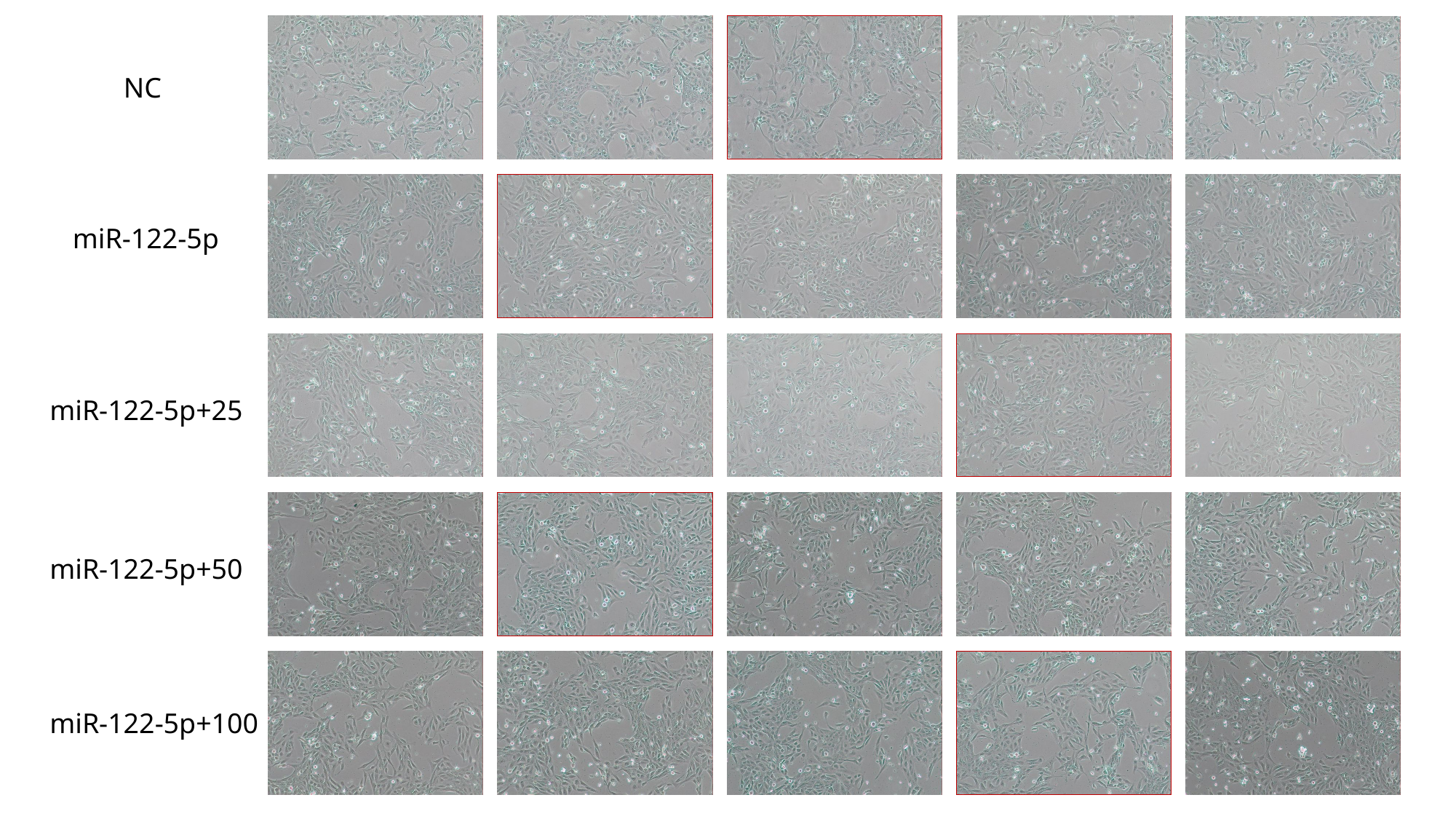

NC
miR-122-5p
miR-122-5p+25
miR-122-5p+50
miR-122-5p+100

Supplement: Supplementary file 4 [file DataSheet2.zip › C/Figure.pptx]

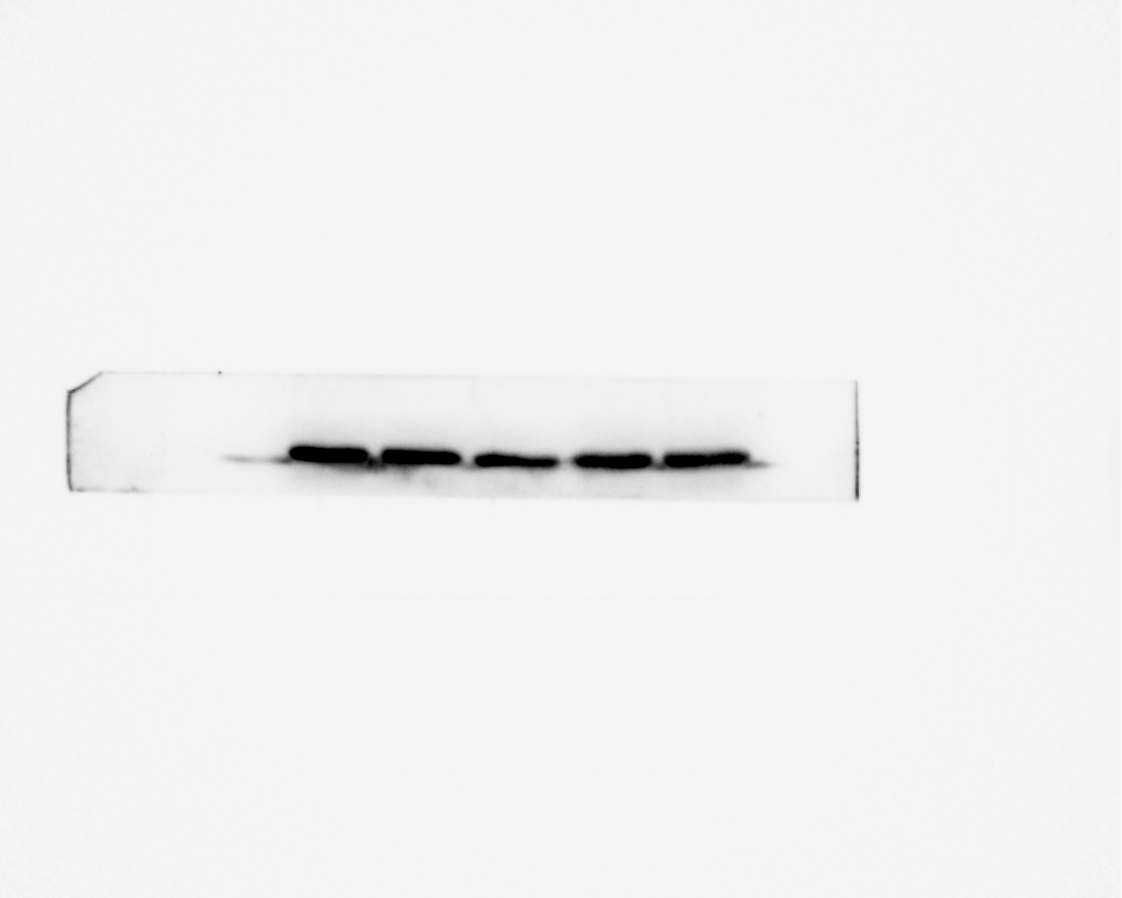

Supplement: Supplementary file 5 [file DataSheet5.zip › Figure 8-WB figure/BAX/6-14 GAPDH 1.tif]

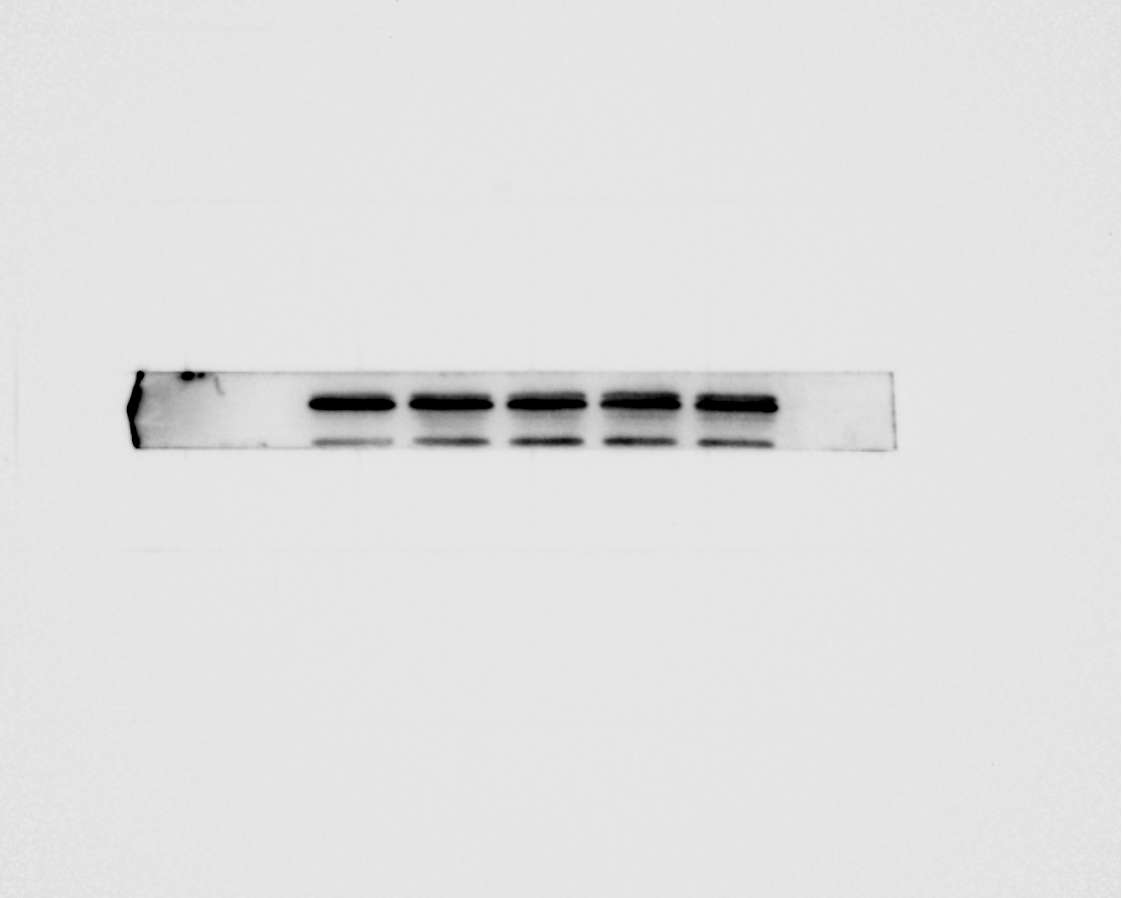

Supplement: Supplementary file 5 [file DataSheet5.zip › Figure 8-WB figure/BAX/6-14 GAPDH 2.tif]

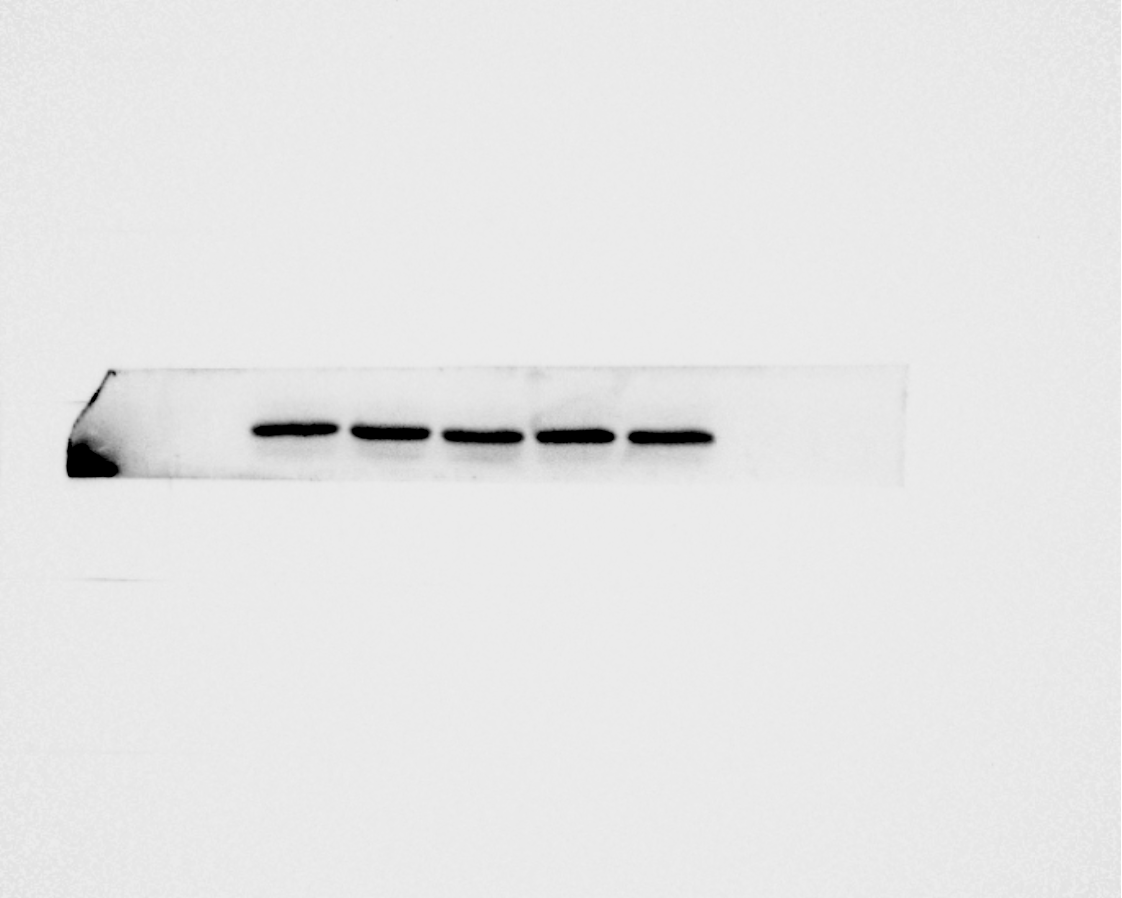

Supplement: Supplementary file 5 [file DataSheet5.zip › Figure 8-WB figure/BAX/6-14 GAPDH 3.tif]

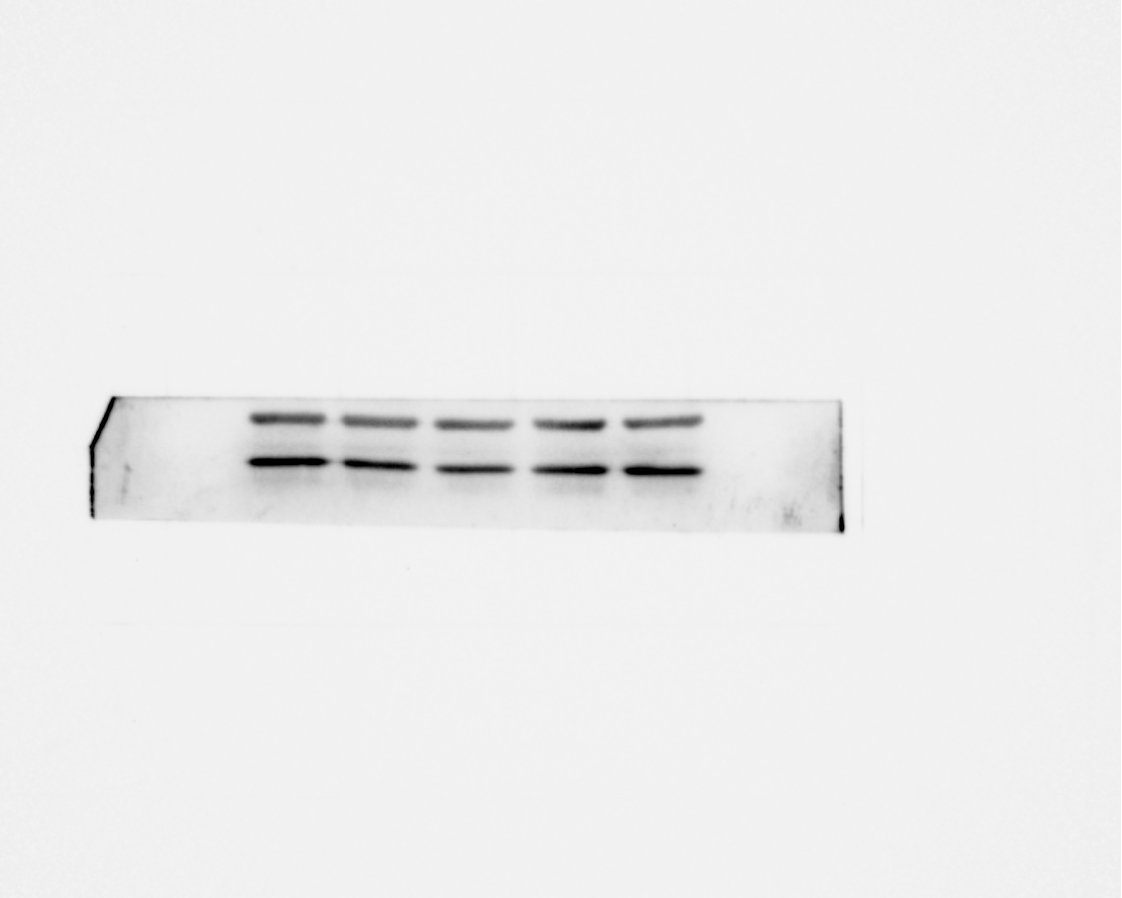

Supplement: Supplementary file 5 [file DataSheet5.zip › Figure 8-WB figure/BAX/6-14 bax 1.tif]

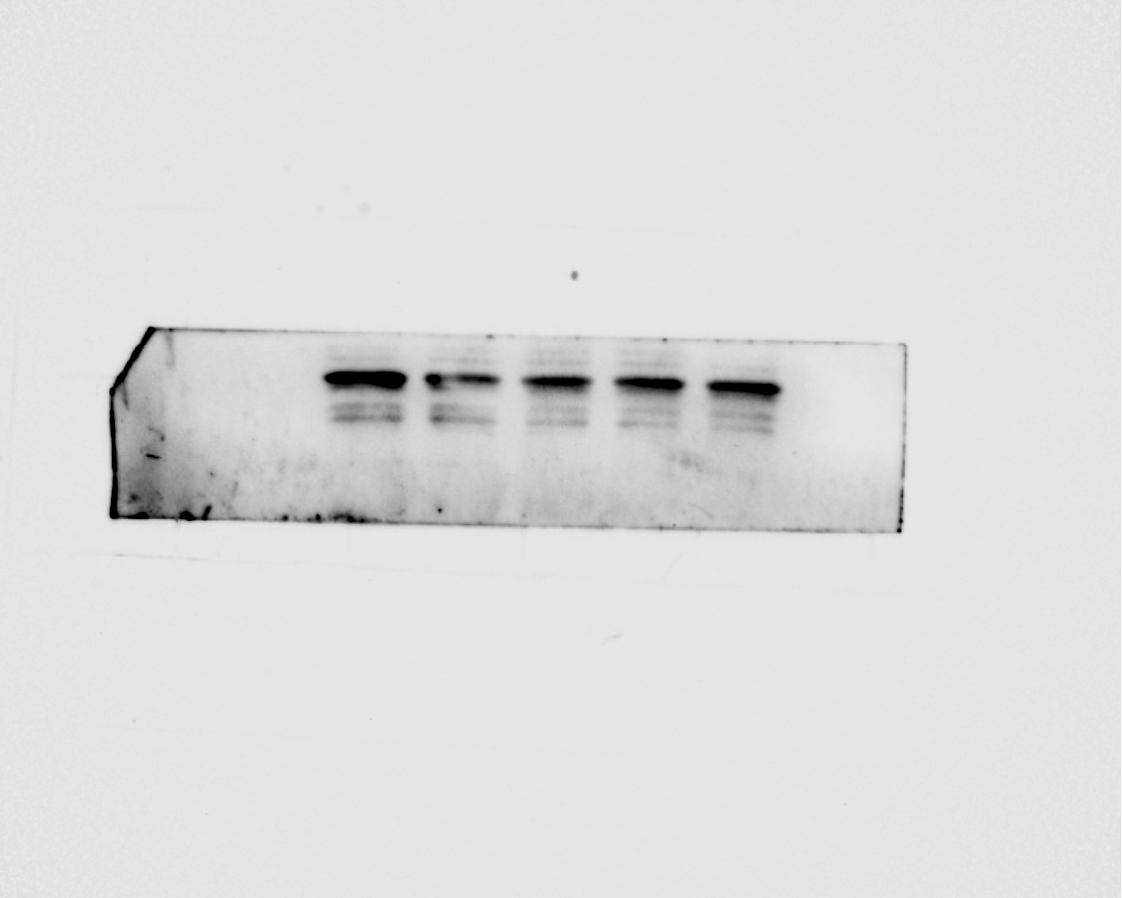

Supplement: Supplementary file 5 [file DataSheet5.zip › Figure 8-WB figure/BAX/6-14 bax 2.tif]

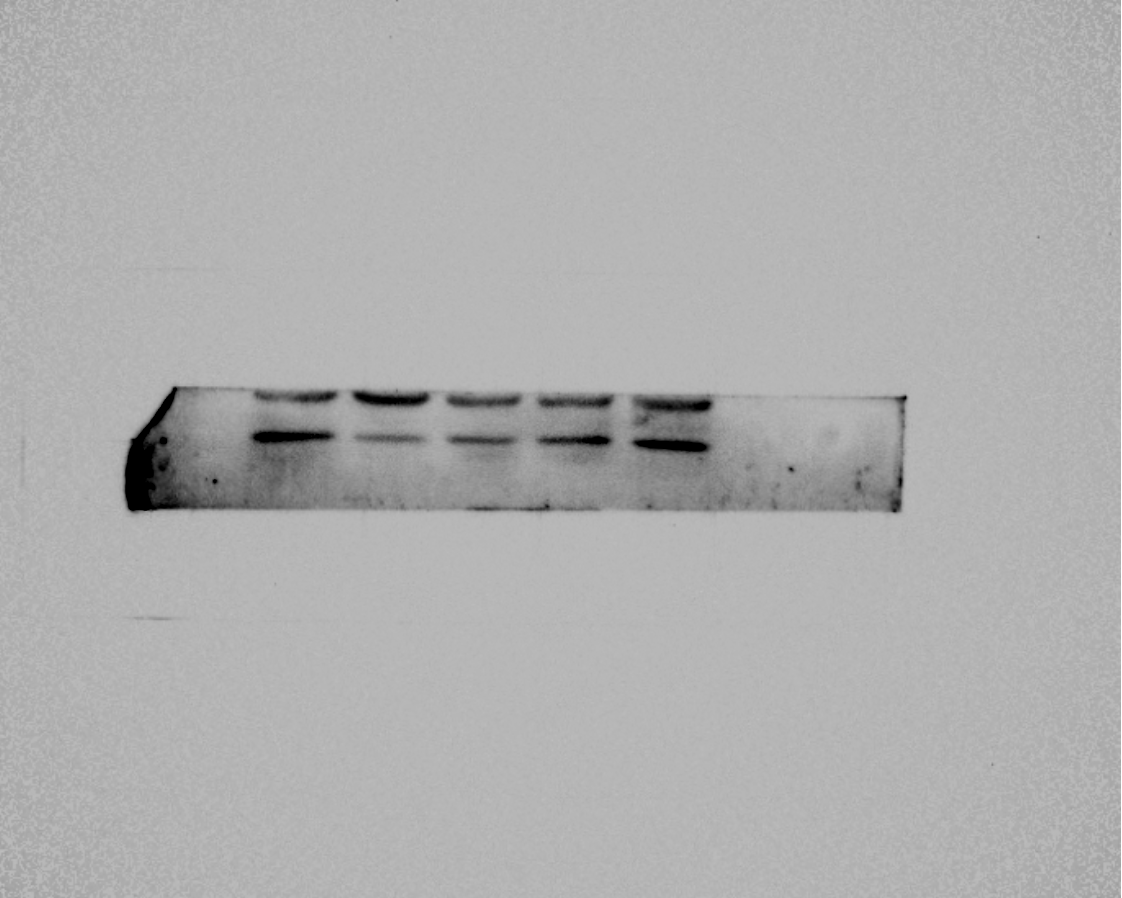

Supplement: Supplementary file 5 [file DataSheet5.zip › Figure 8-WB figure/BAX/6-14 bax 3.tif]

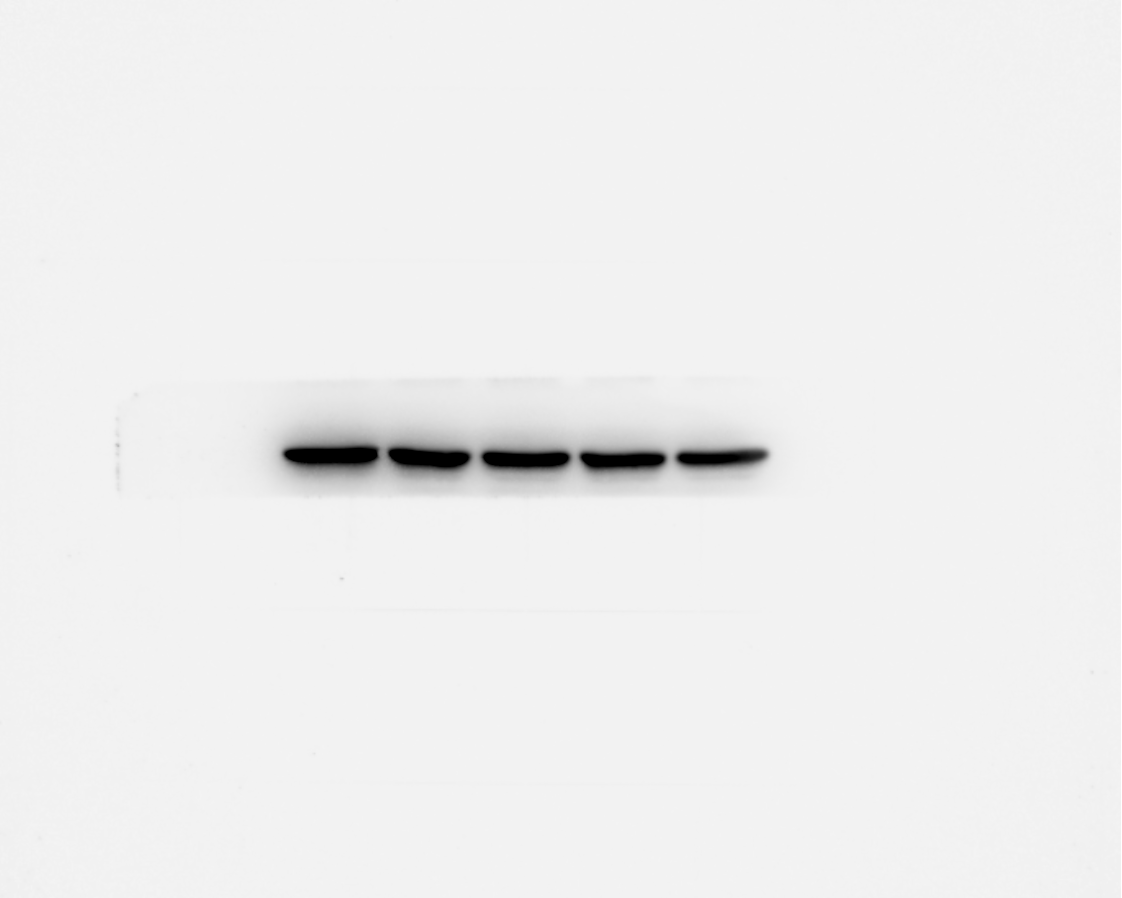

Supplement: Supplementary file 5 [file DataSheet5.zip › Figure 8-WB figure/BCL2/GAPDH 1.tif]

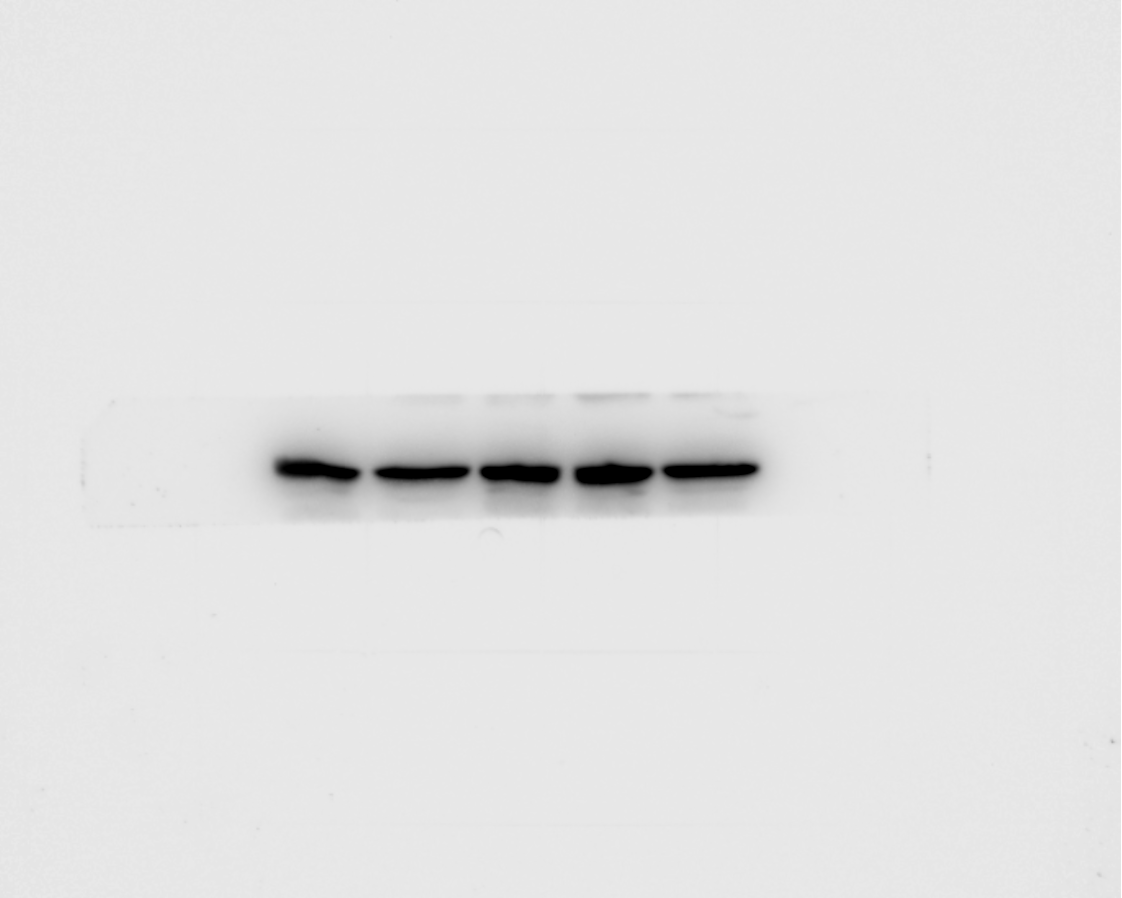

Supplement: Supplementary file 5 [file DataSheet5.zip › Figure 8-WB figure/BCL2/GAPDH 2.tif]

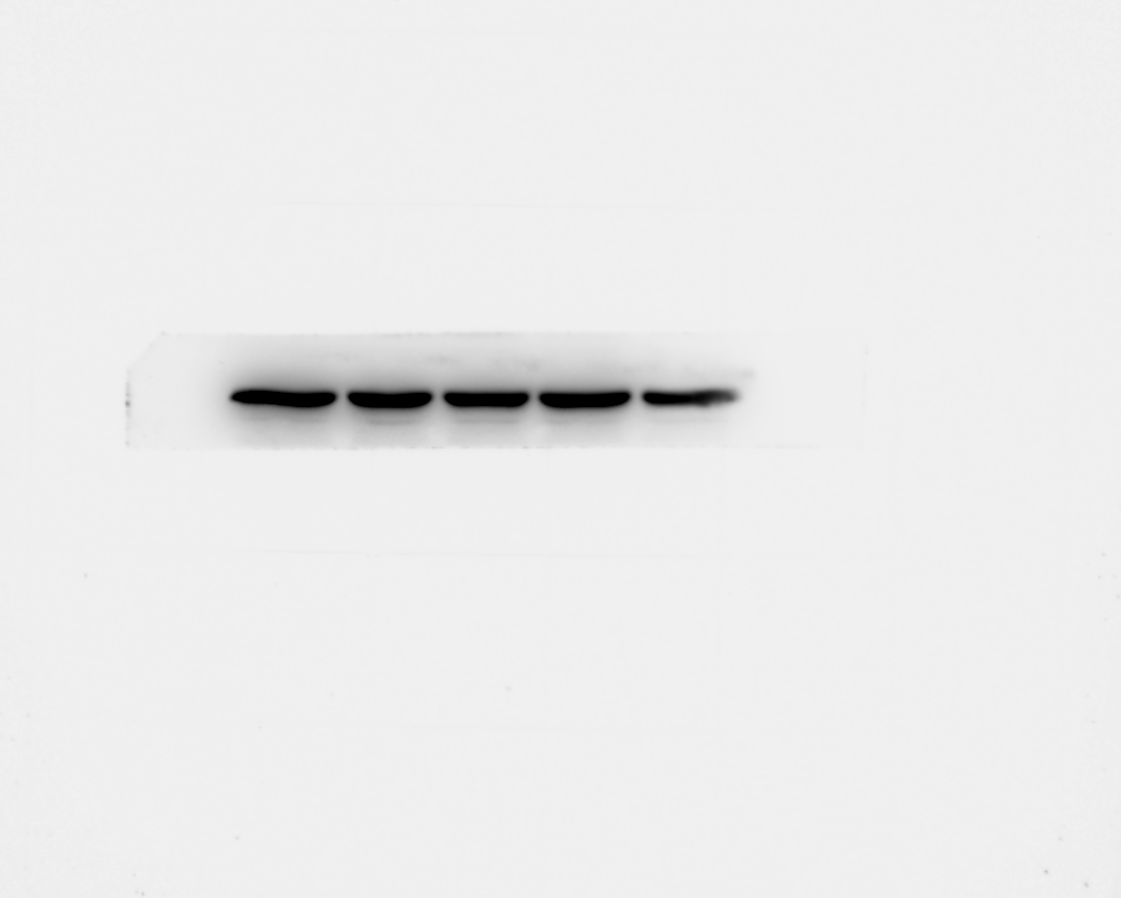

Supplement: Supplementary file 5 [file DataSheet5.zip › Figure 8-WB figure/BCL2/GAPDH 3.tif]

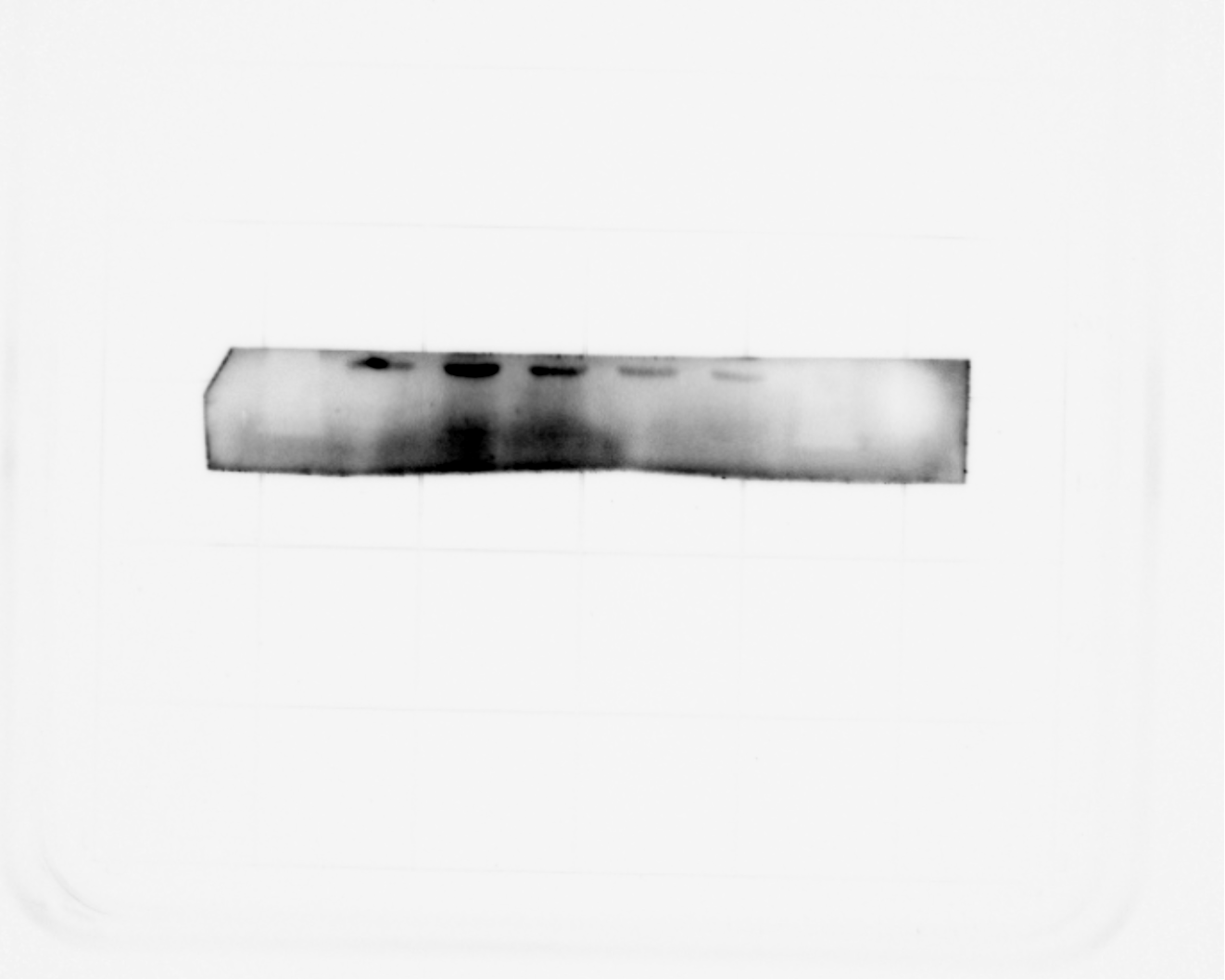

Supplement: Supplementary file 5 [file DataSheet5.zip › Figure 8-WB figure/BCL2/bcl2 1.tif]

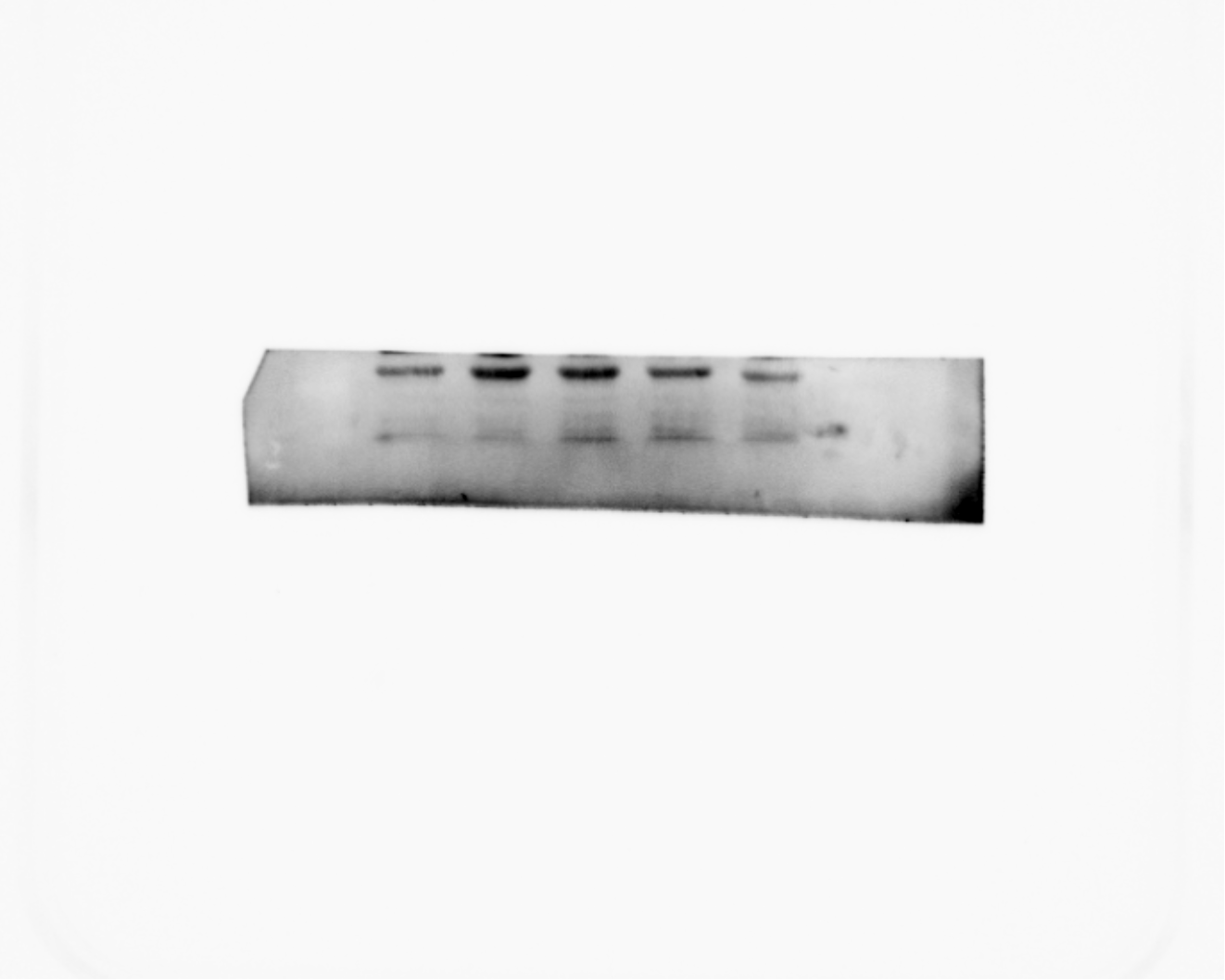

Supplement: Supplementary file 5 [file DataSheet5.zip › Figure 8-WB figure/BCL2/bcl2 2.tif]

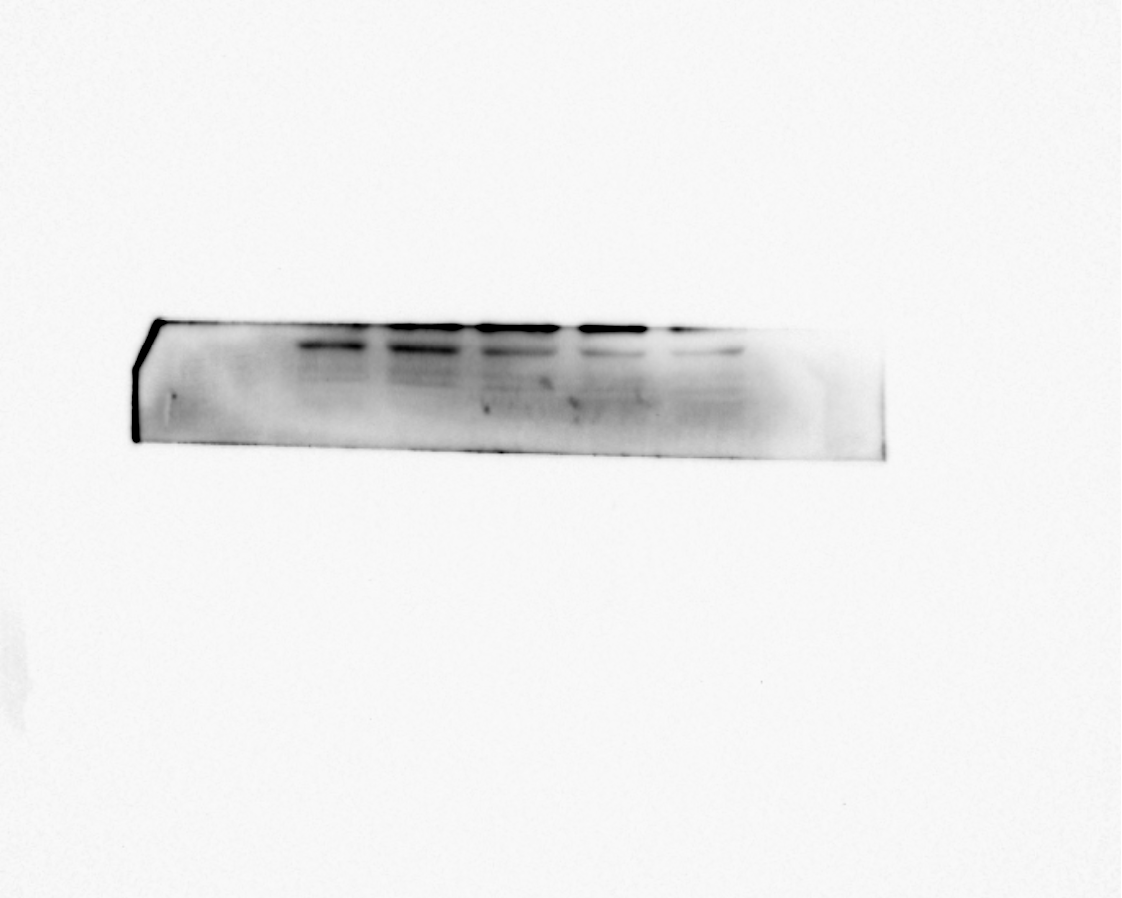

Supplement: Supplementary file 5 [file DataSheet5.zip › Figure 8-WB figure/BCL2/bcl2 3.tif]

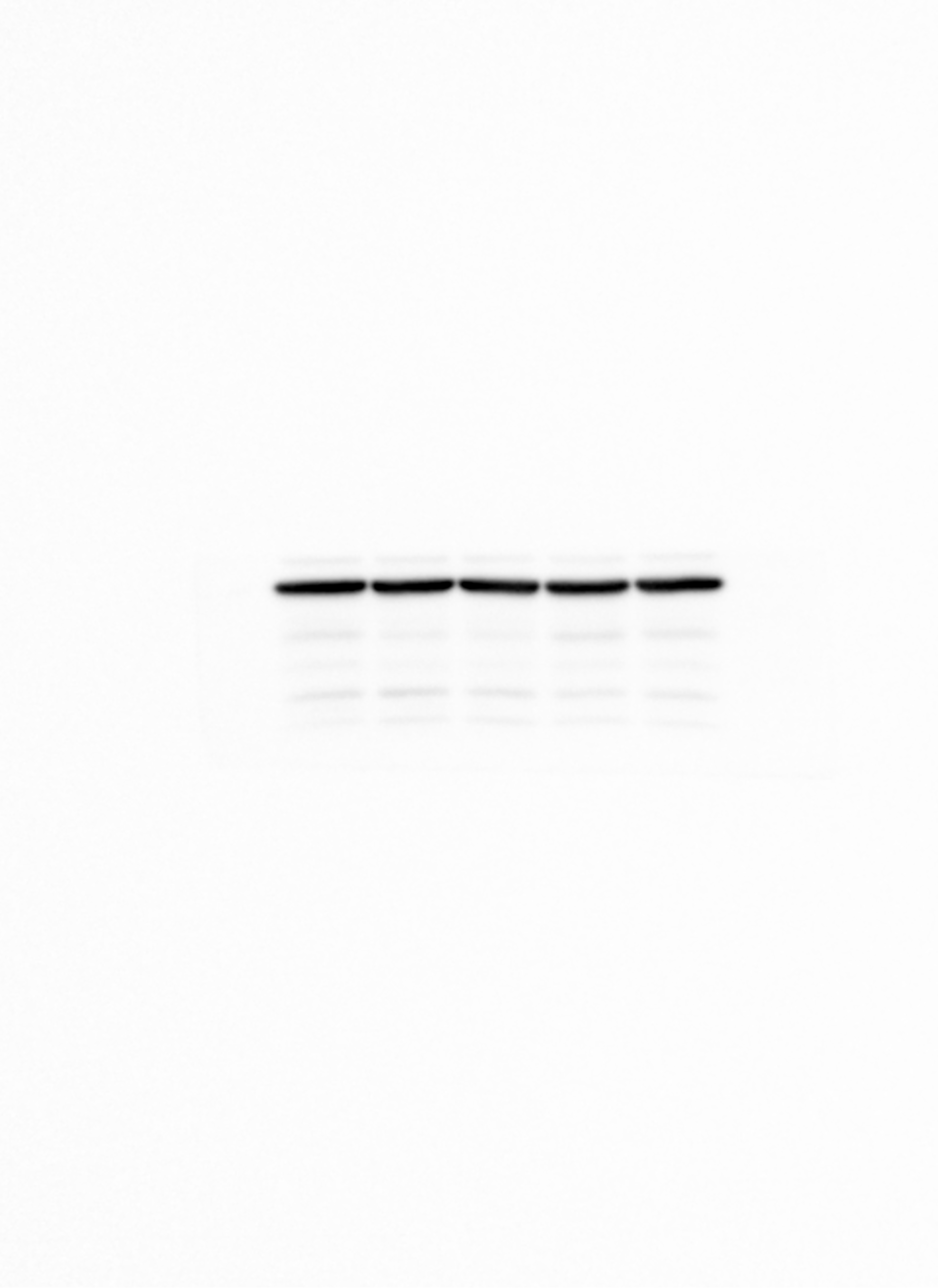

Supplement: Supplementary file 5 [file DataSheet5.zip › Figure 8-WB figure/PTEN/gap1 20231228_143346_Ch_Chemi.tif]

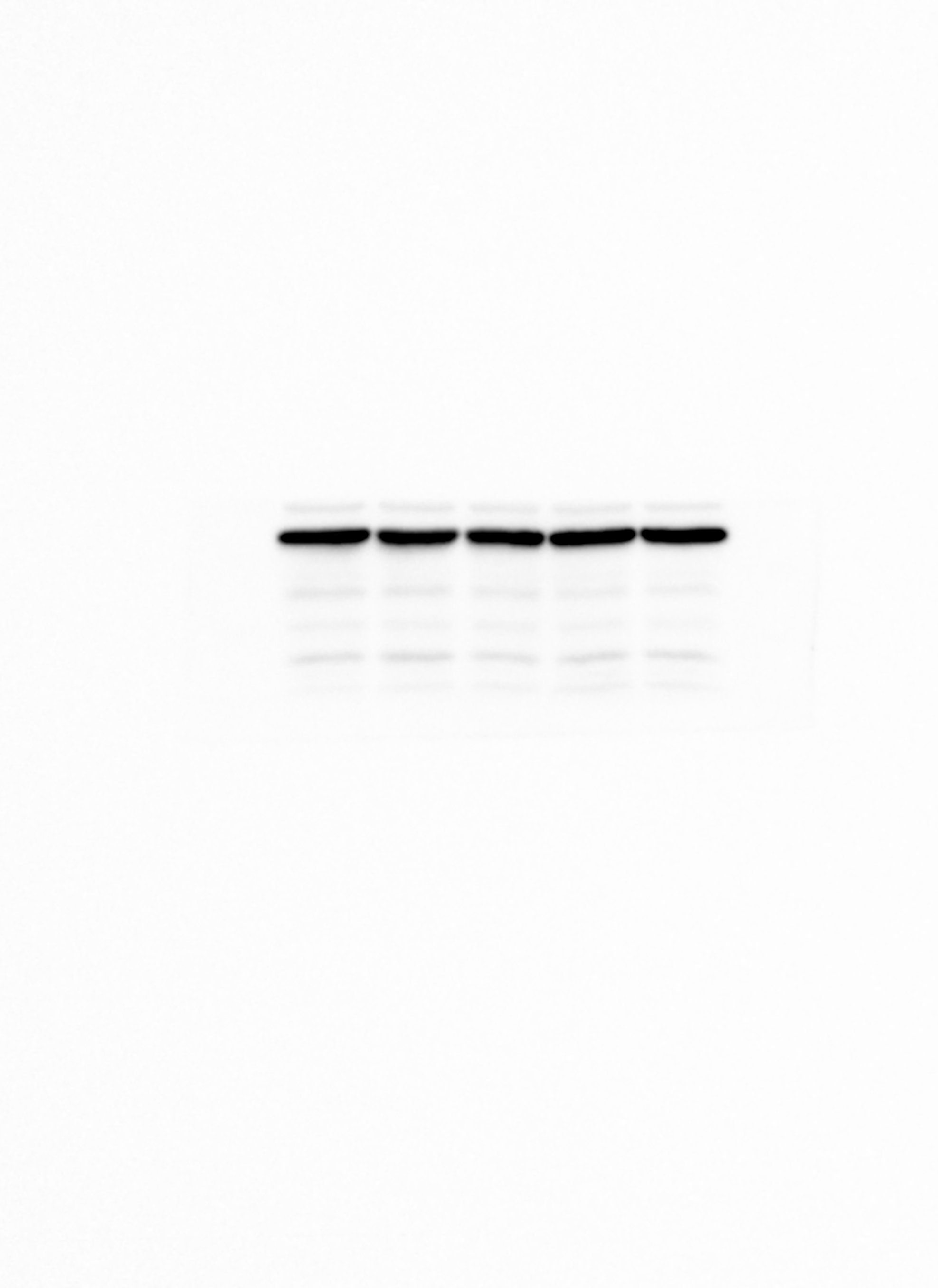

Supplement: Supplementary file 5 [file DataSheet5.zip › Figure 8-WB figure/PTEN/gap2 20231228_143027_Ch_Chemi.tif]

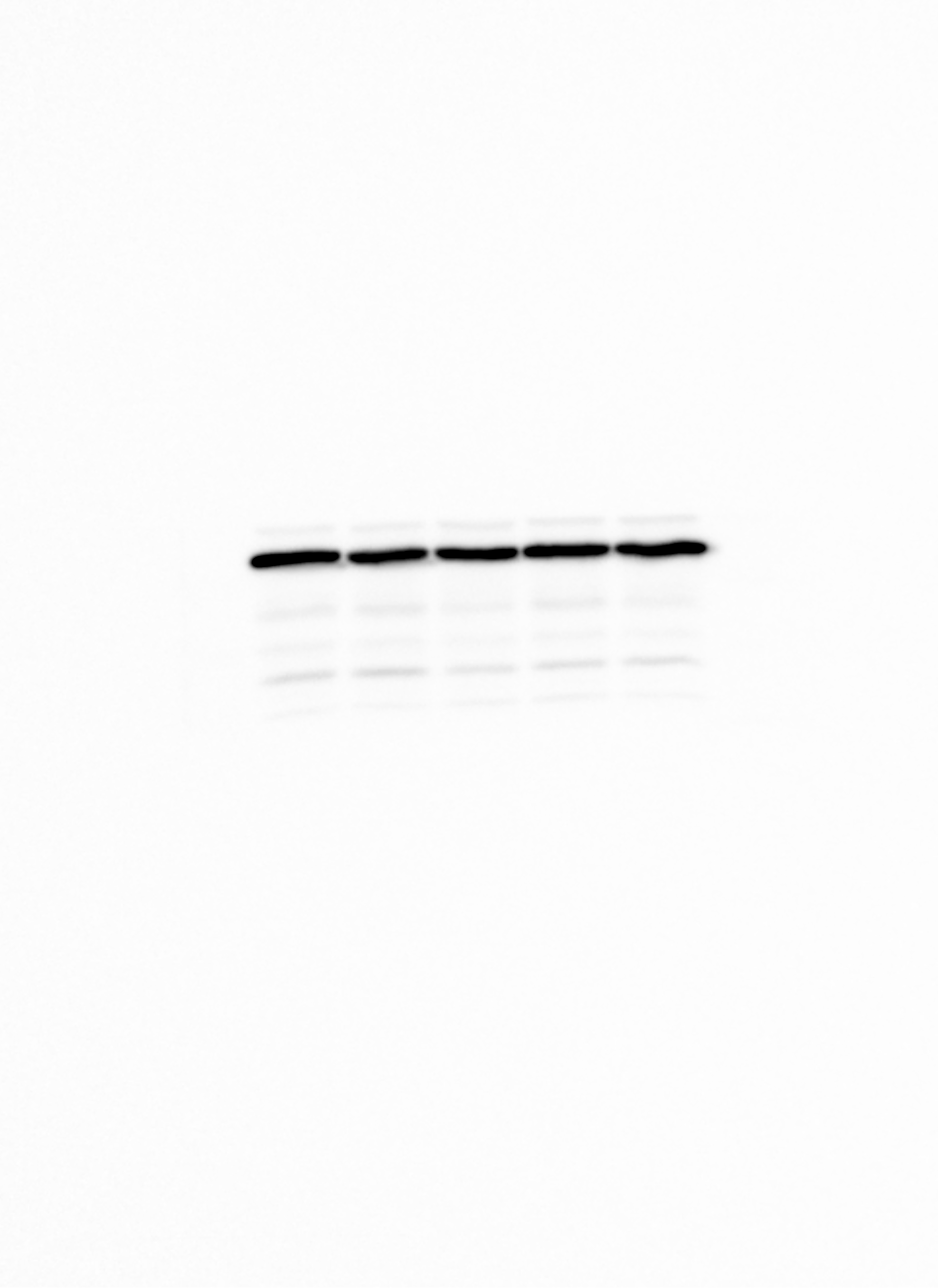

Supplement: Supplementary file 5 [file DataSheet5.zip › Figure 8-WB figure/PTEN/gap3 20231228_142657_Ch_Chemi.tif]

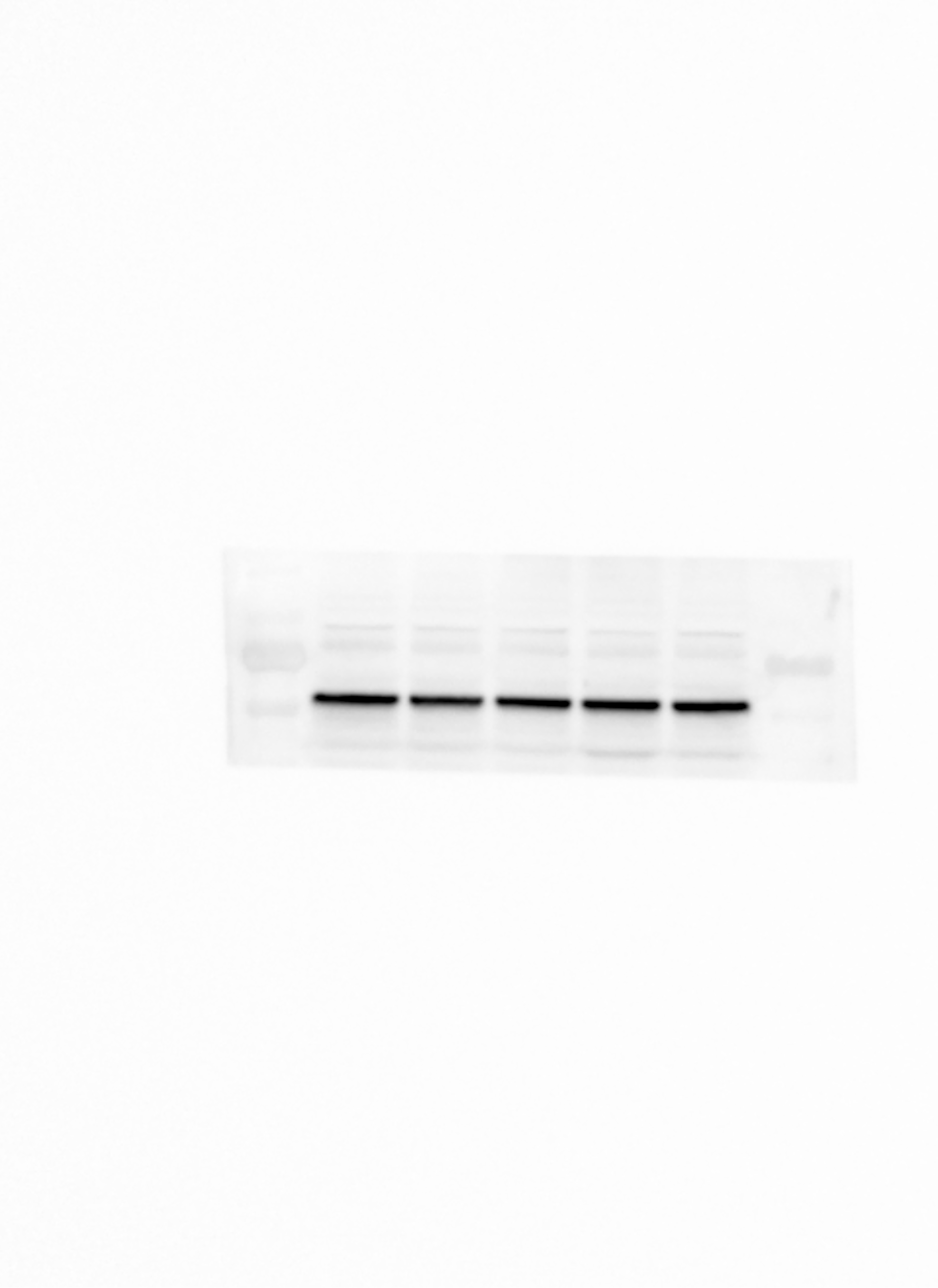

Supplement: Supplementary file 5 [file DataSheet5.zip › Figure 8-WB figure/PTEN/pten1 20231228_144904_Ch_Chemi.tif]

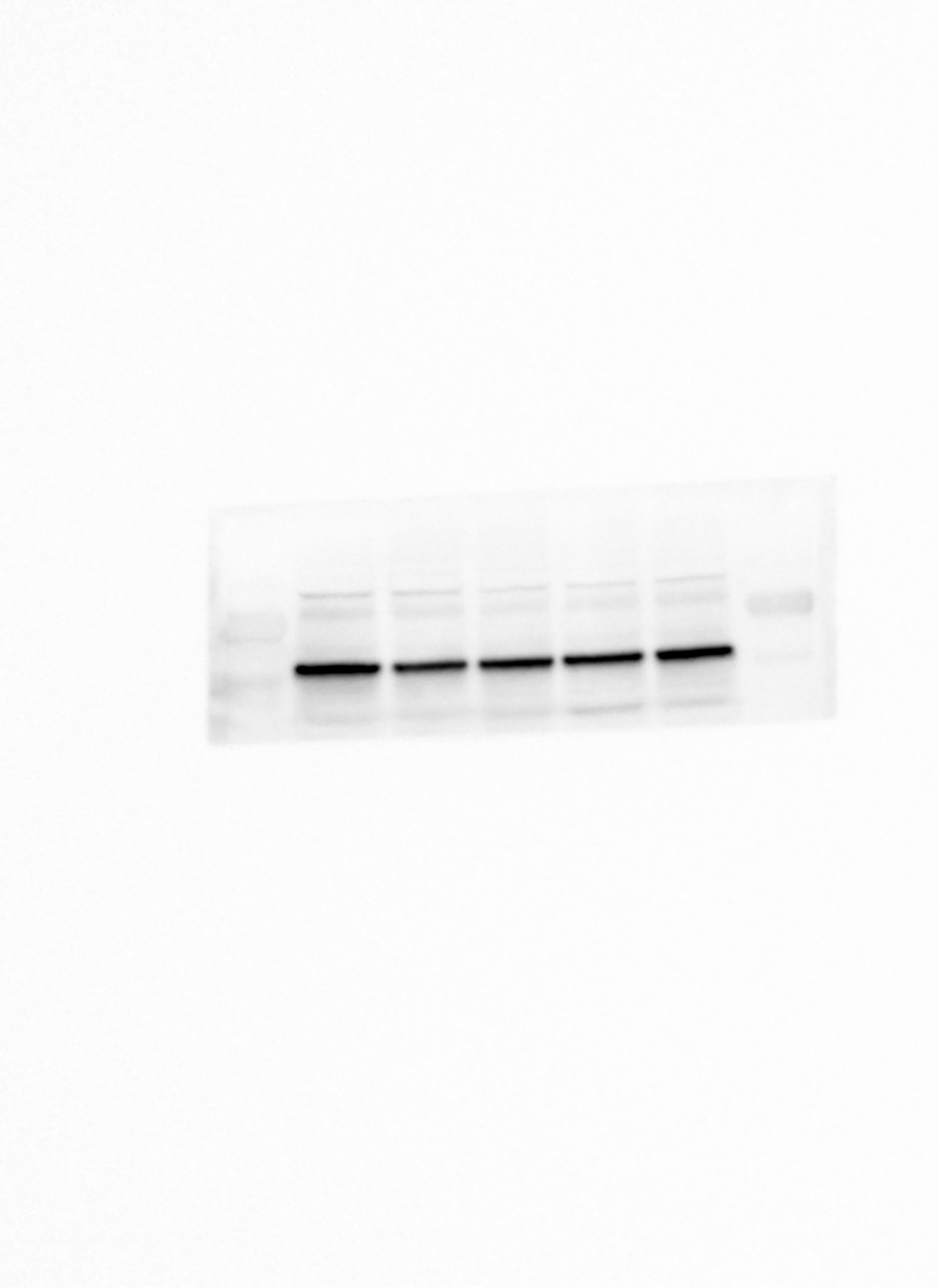

Supplement: Supplementary file 5 [file DataSheet5.zip › Figure 8-WB figure/PTEN/pten2 20231228_143817_Ch_Chemi.tif]

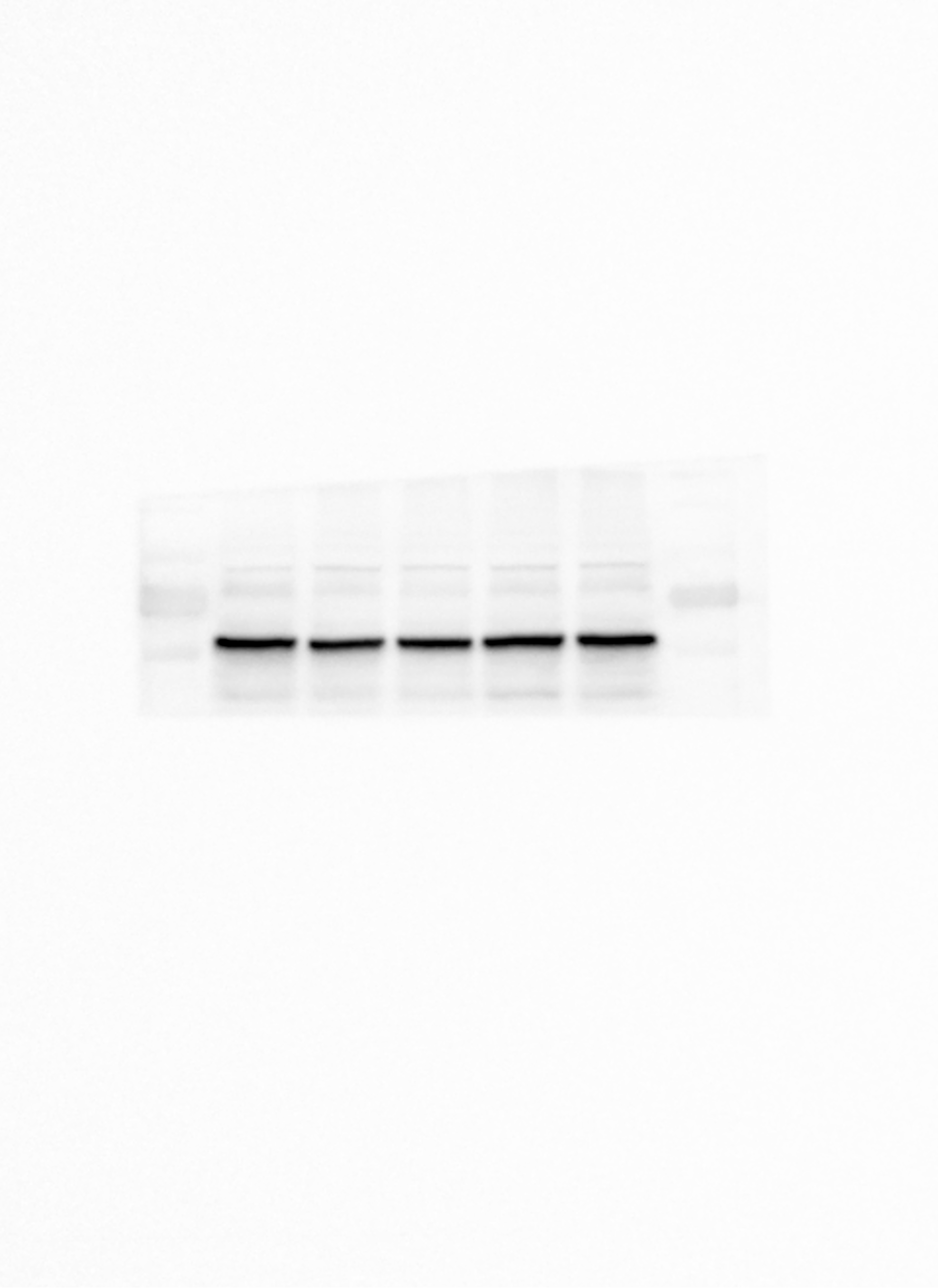

Supplement: Supplementary file 5 [file DataSheet5.zip › Figure 8-WB figure/PTEN/pten3 20231228_144204_Ch_Chemi.tif]

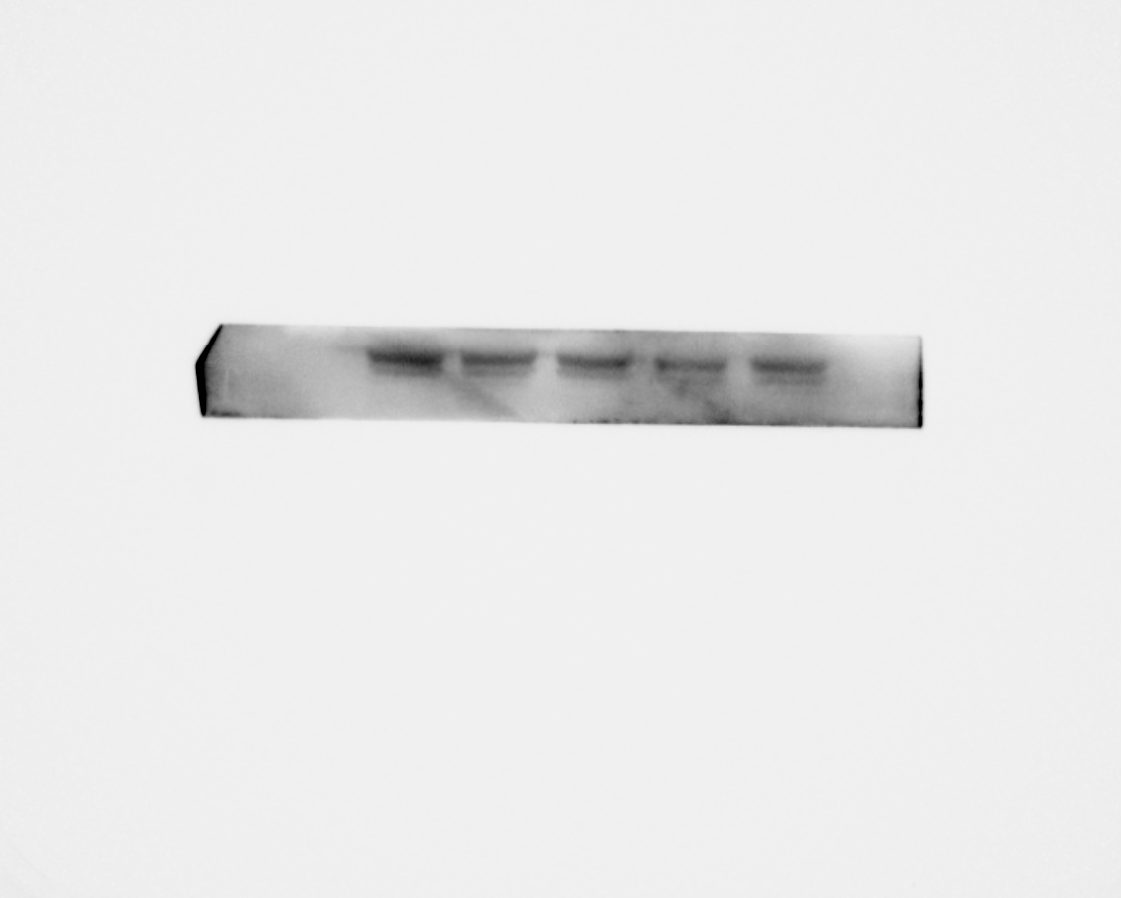

Supplement: Supplementary file 5 [file DataSheet5.zip › Figure 8-WB figure/p-AKT、AKT/AKT 1.tif]

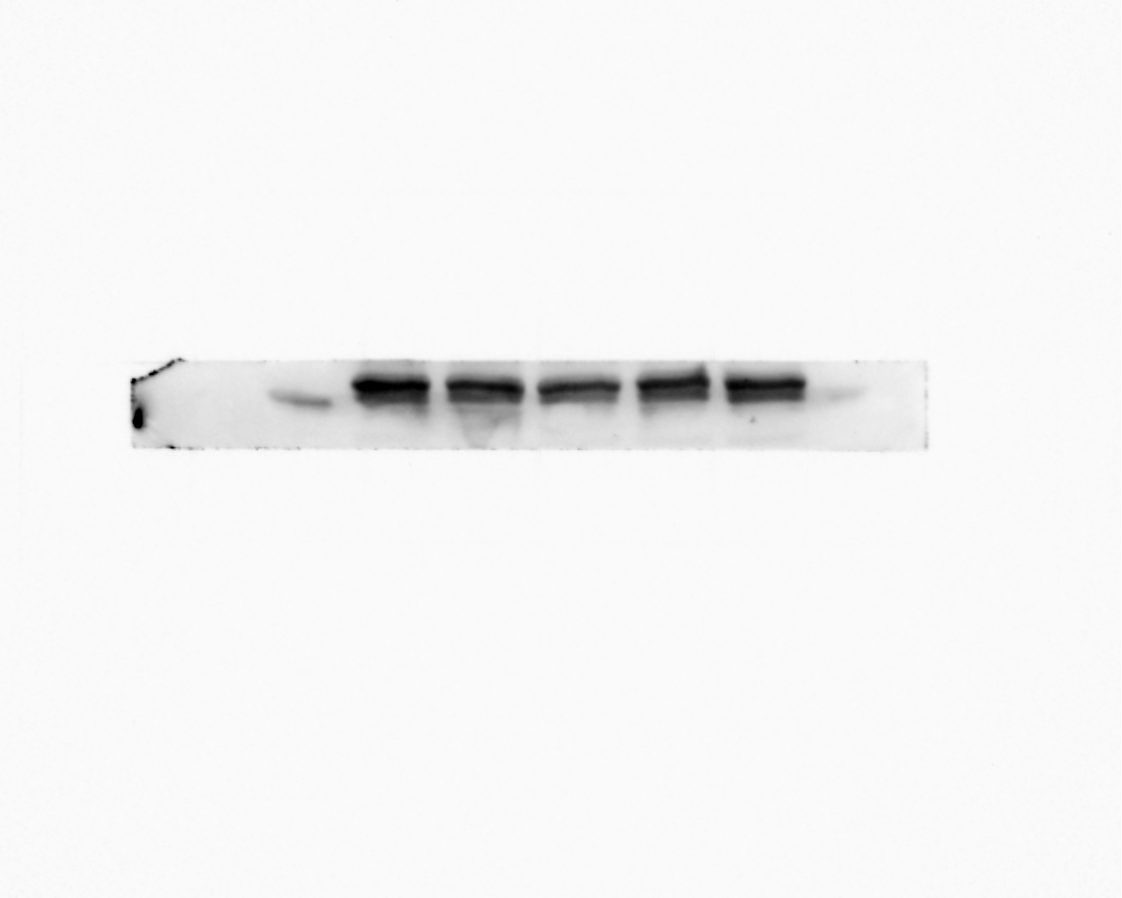

Supplement: Supplementary file 5 [file DataSheet5.zip › Figure 8-WB figure/p-AKT、AKT/AKT 2.tif]

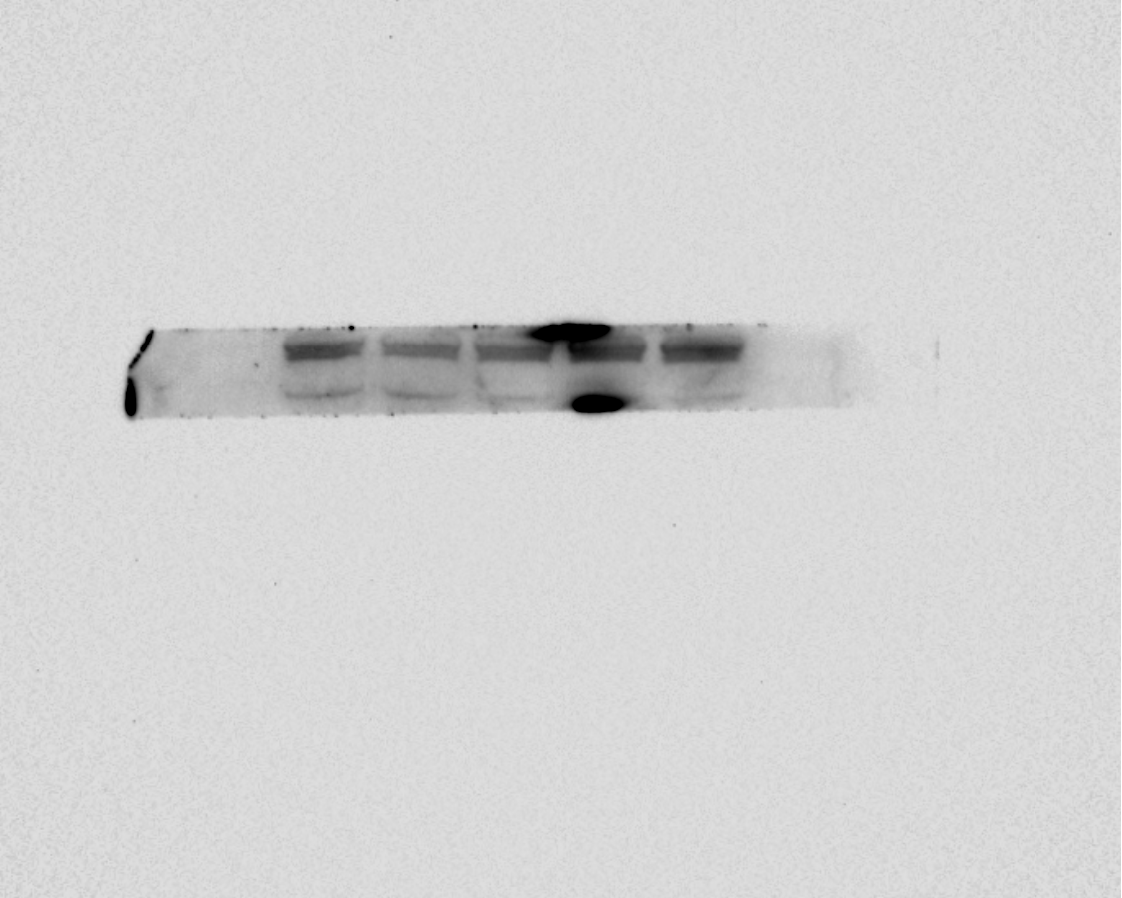

Supplement: Supplementary file 5 [file DataSheet5.zip › Figure 8-WB figure/p-AKT、AKT/AKT 3.tif]

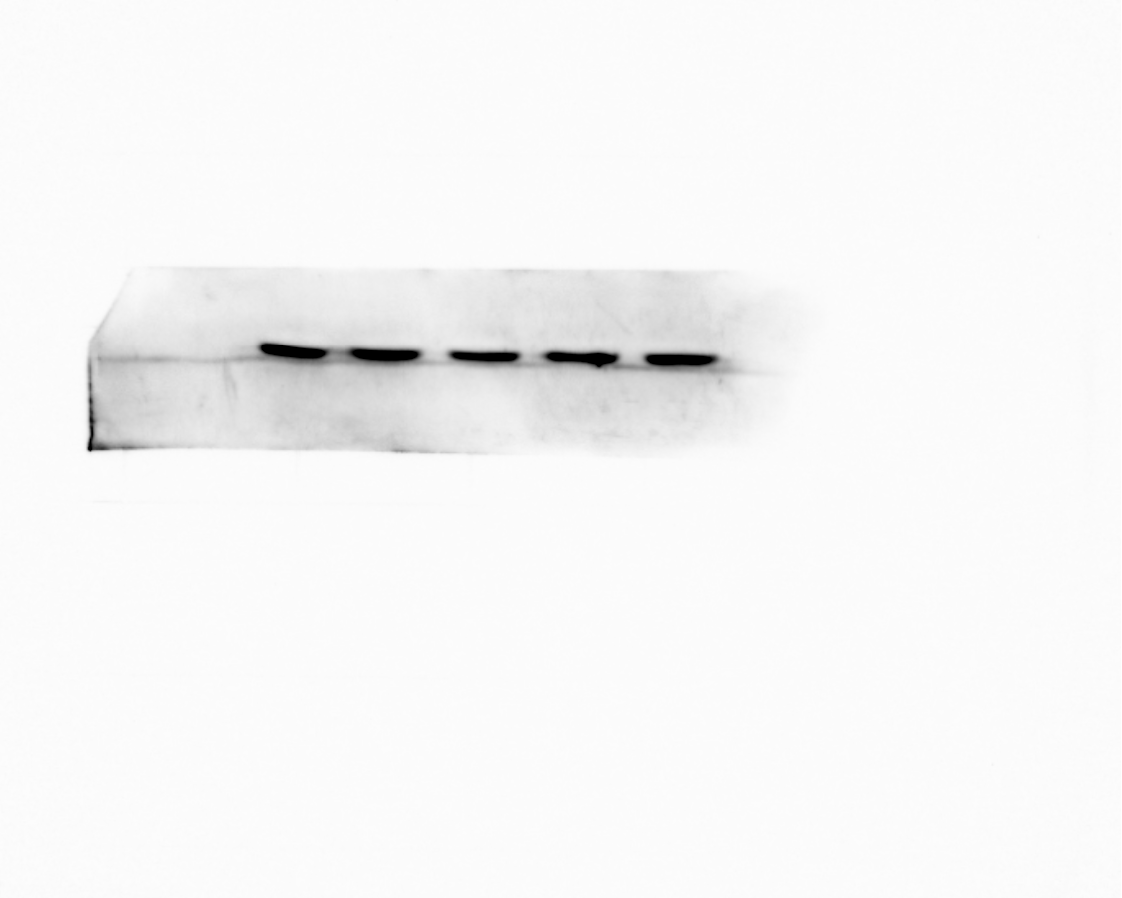

Supplement: Supplementary file 5 [file DataSheet5.zip › Figure 8-WB figure/p-AKT、AKT/GAPDH 1.tif]

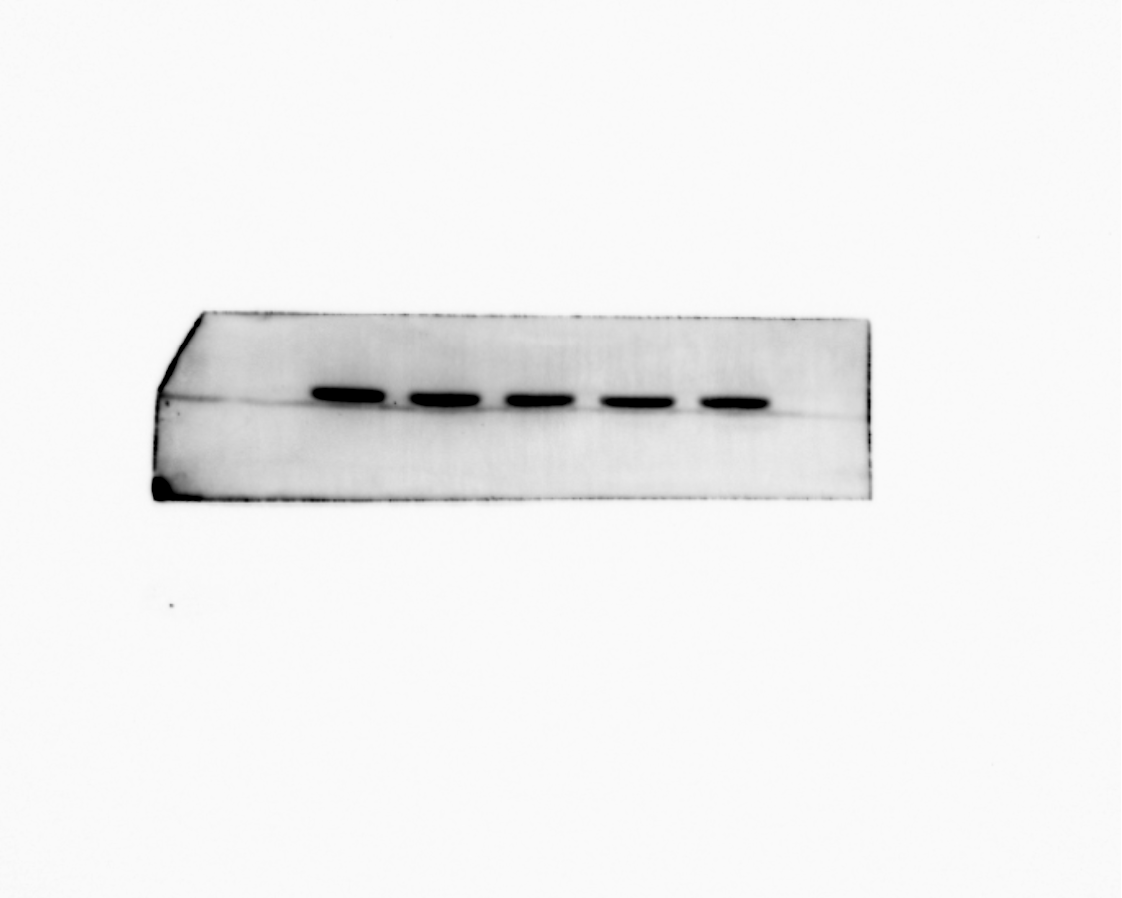

Supplement: Supplementary file 5 [file DataSheet5.zip › Figure 8-WB figure/p-AKT、AKT/GAPDH 2.tif]

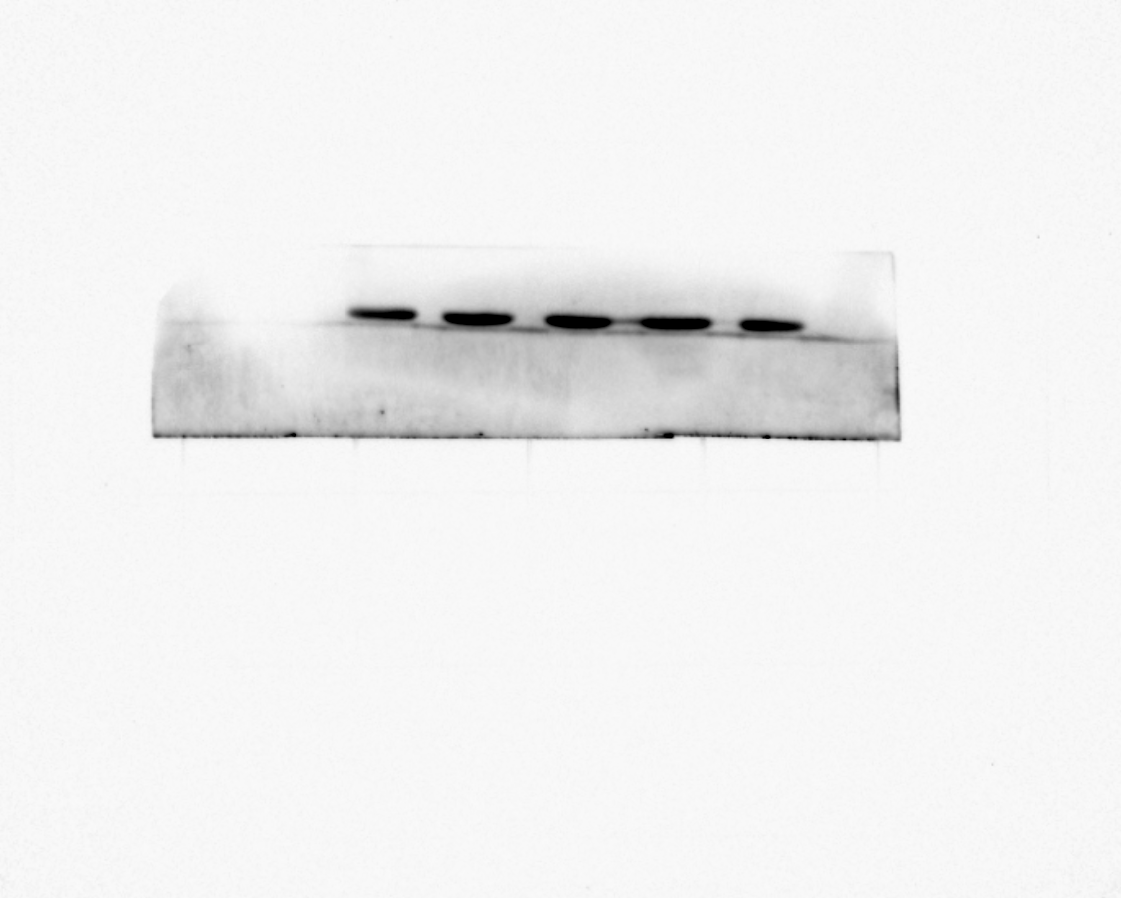

Supplement: Supplementary file 5 [file DataSheet5.zip › Figure 8-WB figure/p-AKT、AKT/GAPDH 3.tif]

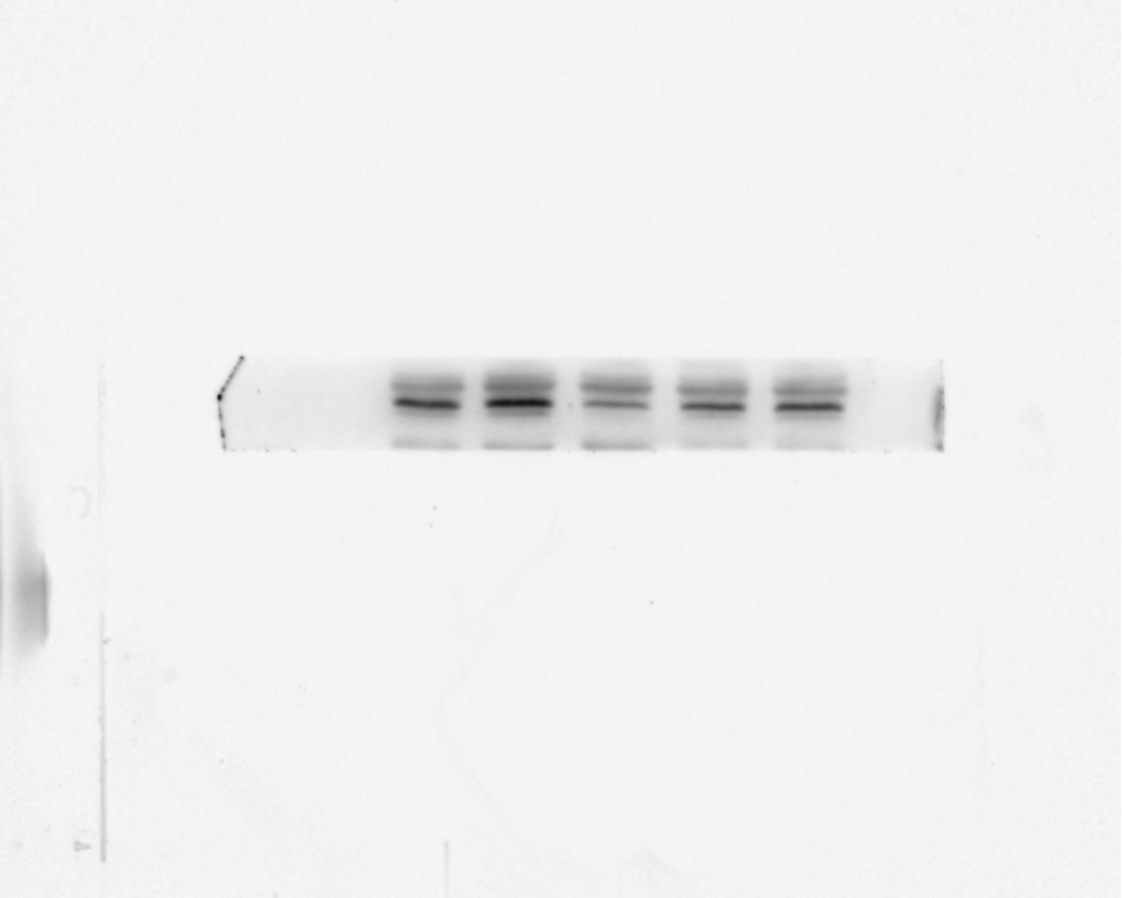

Supplement: Supplementary file 5 [file DataSheet5.zip › Figure 8-WB figure/p-AKT、AKT/p-AKT 1.tif]

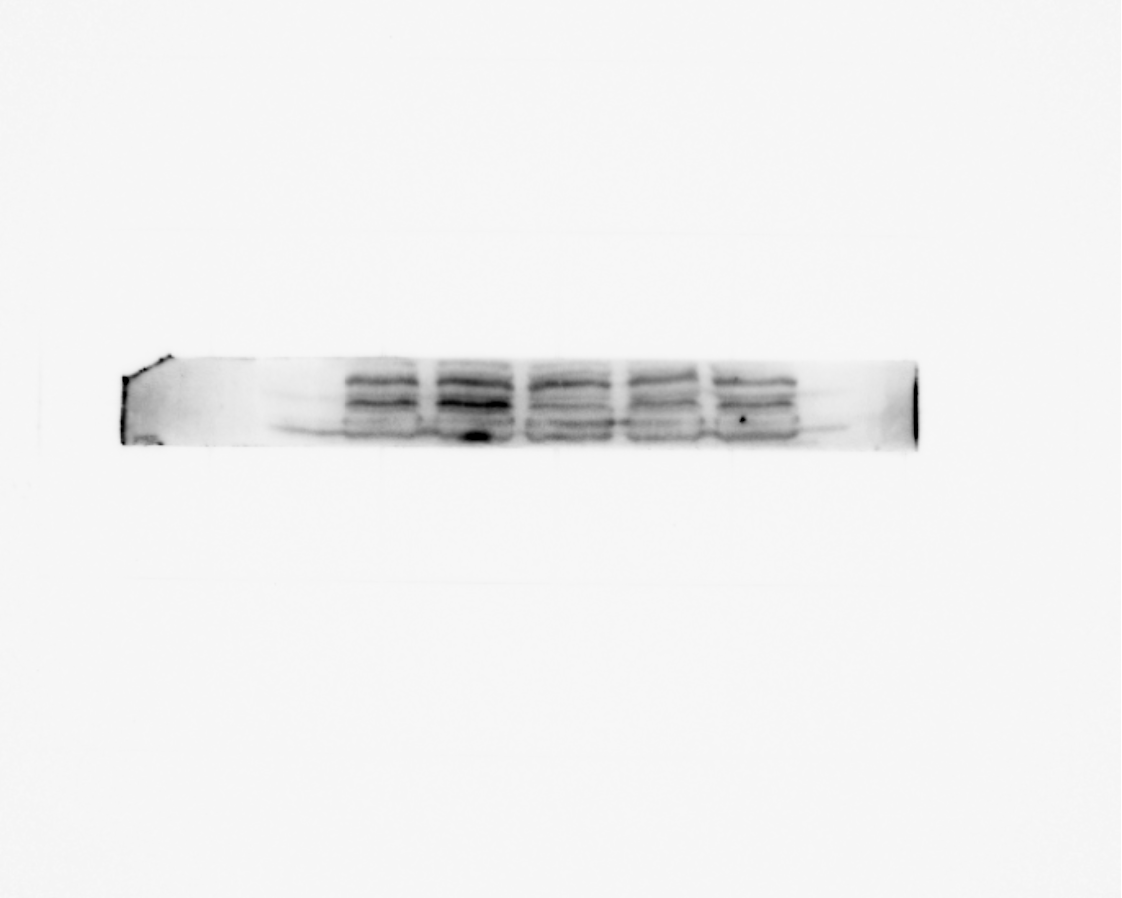

Supplement: Supplementary file 5 [file DataSheet5.zip › Figure 8-WB figure/p-AKT、AKT/p-AKT 2.tif]

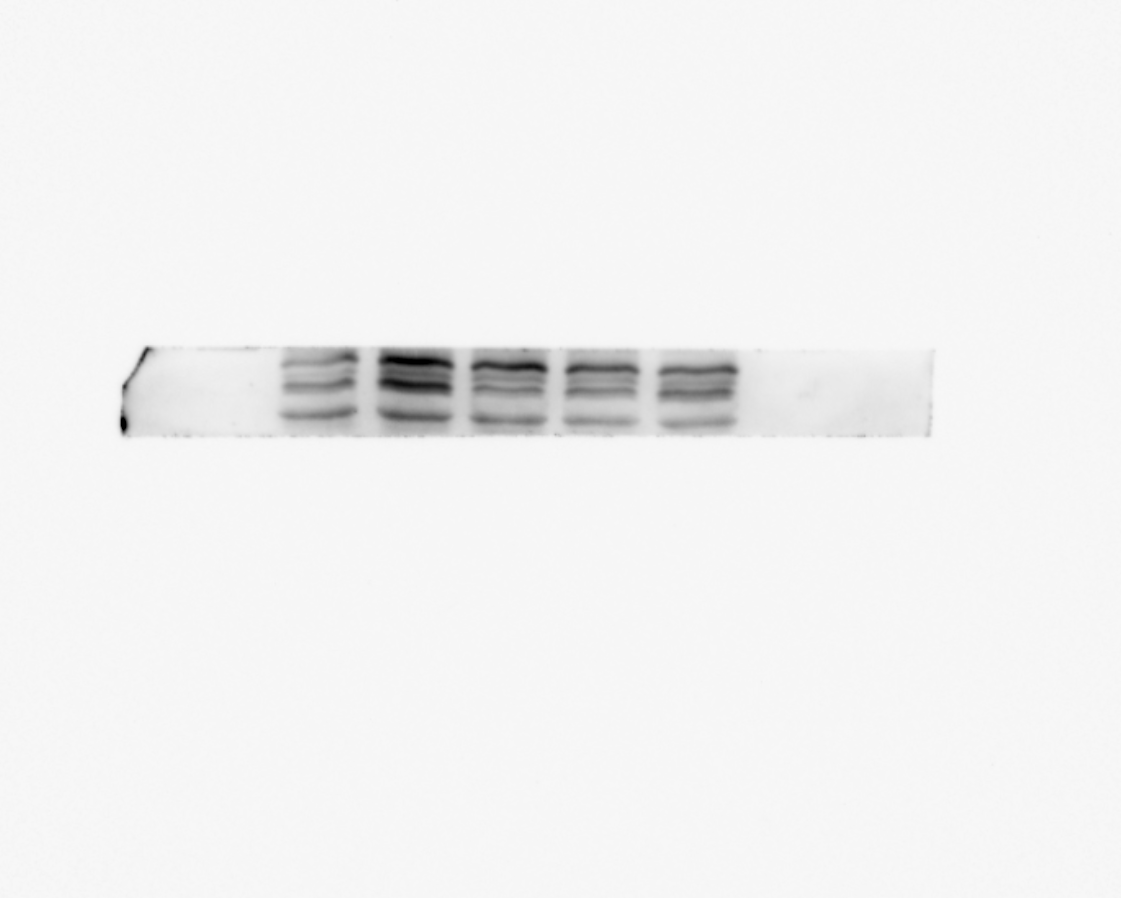

Supplement: Supplementary file 5 [file DataSheet5.zip › Figure 8-WB figure/p-AKT、AKT/p-AKT 3.tif]

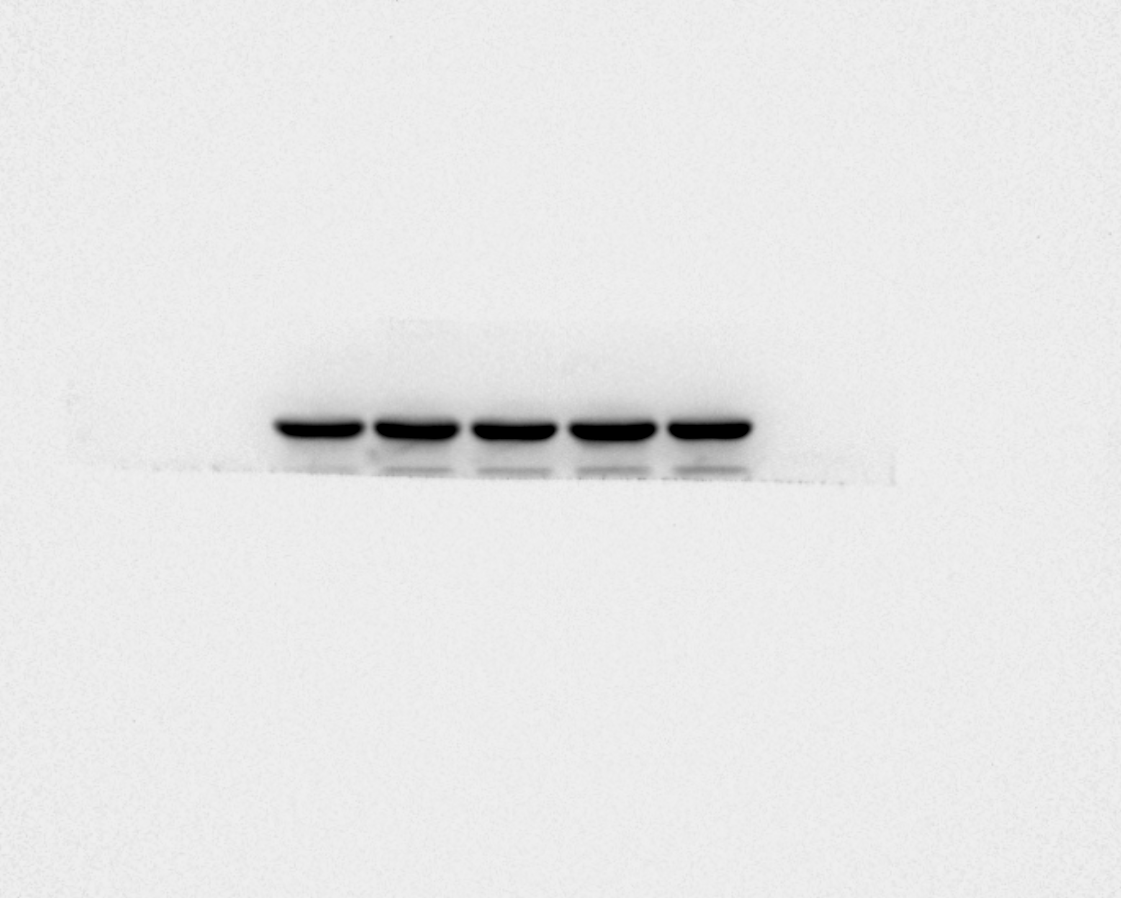

Supplement: Supplementary file 5 [file DataSheet5.zip › Figure 8-WB figure/p-PI3K、PI3K/GAPDH1.tif]

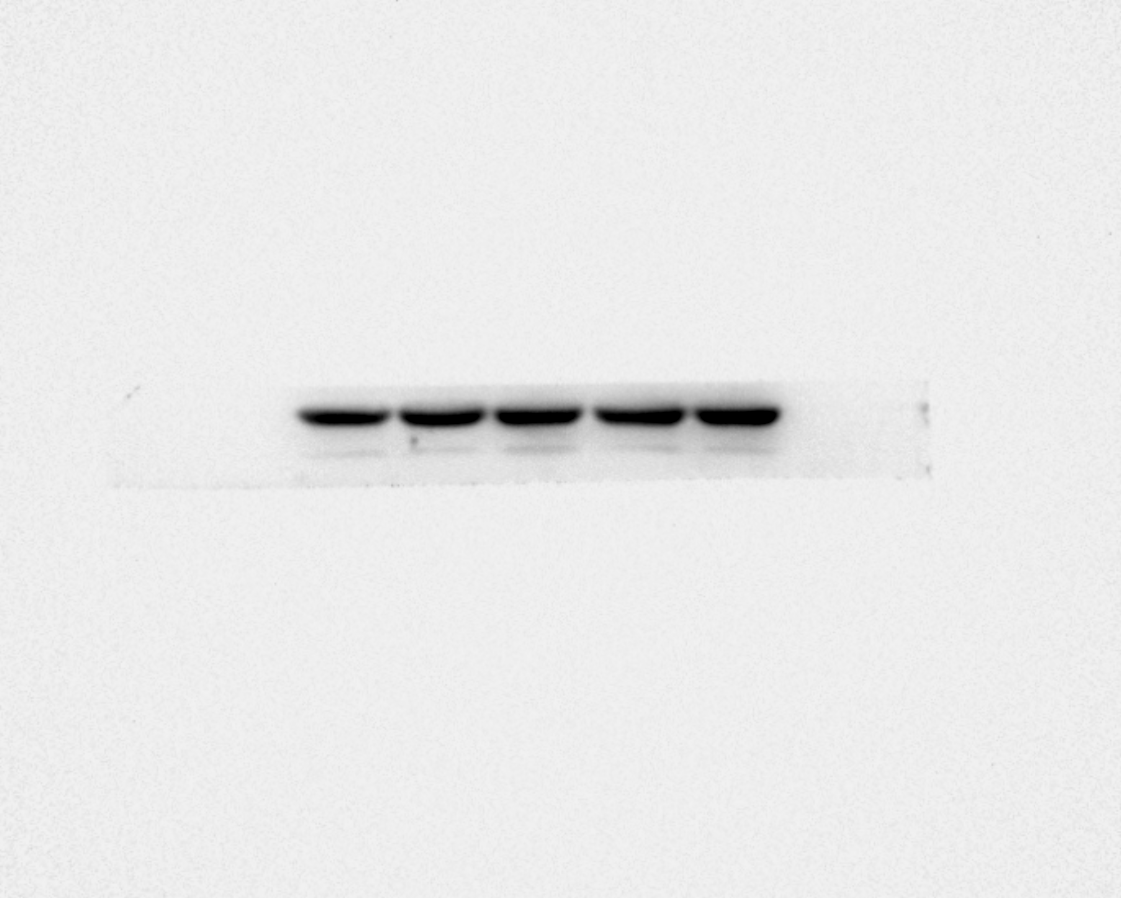

Supplement: Supplementary file 5 [file DataSheet5.zip › Figure 8-WB figure/p-PI3K、PI3K/GAPDH2.tif]

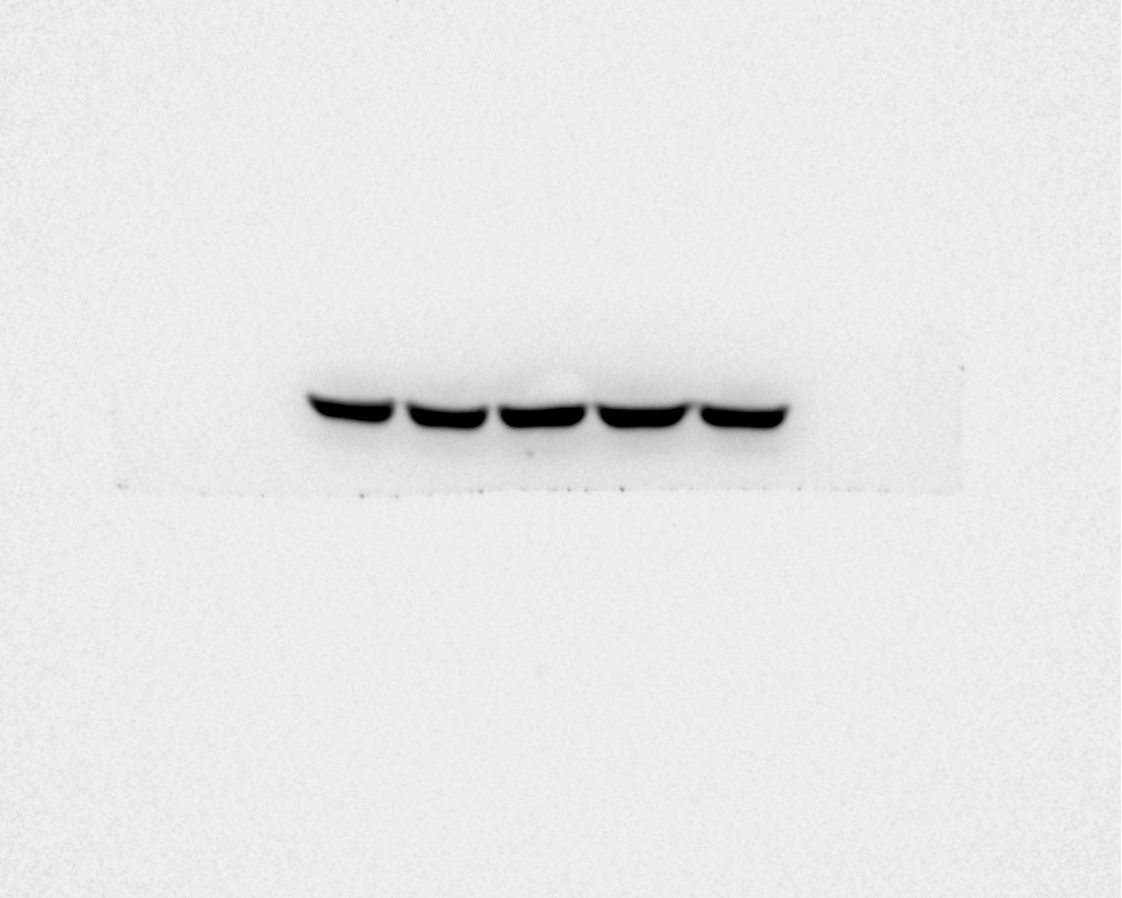

Supplement: Supplementary file 5 [file DataSheet5.zip › Figure 8-WB figure/p-PI3K、PI3K/GAPDH3.tif]

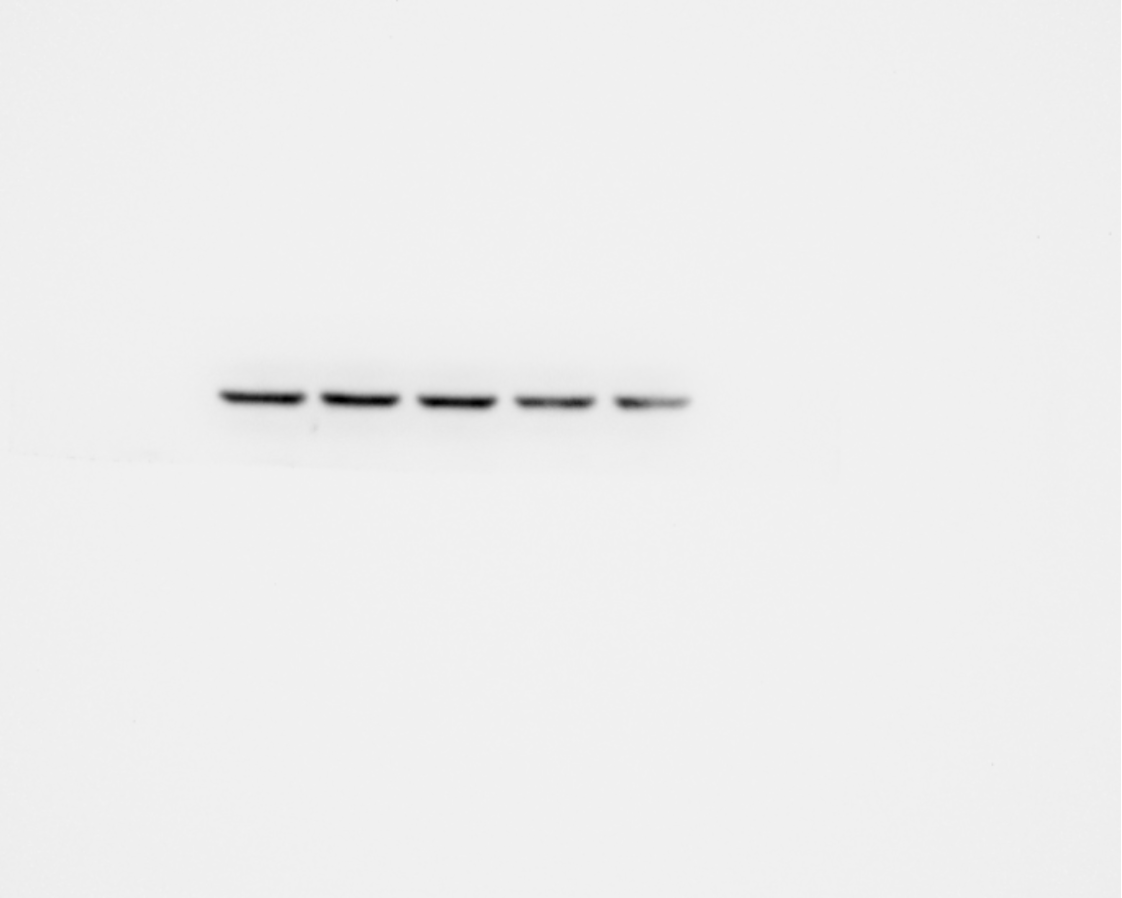

Supplement: Supplementary file 5 [file DataSheet5.zip › Figure 8-WB figure/p-PI3K、PI3K/PCNA 1.tif]

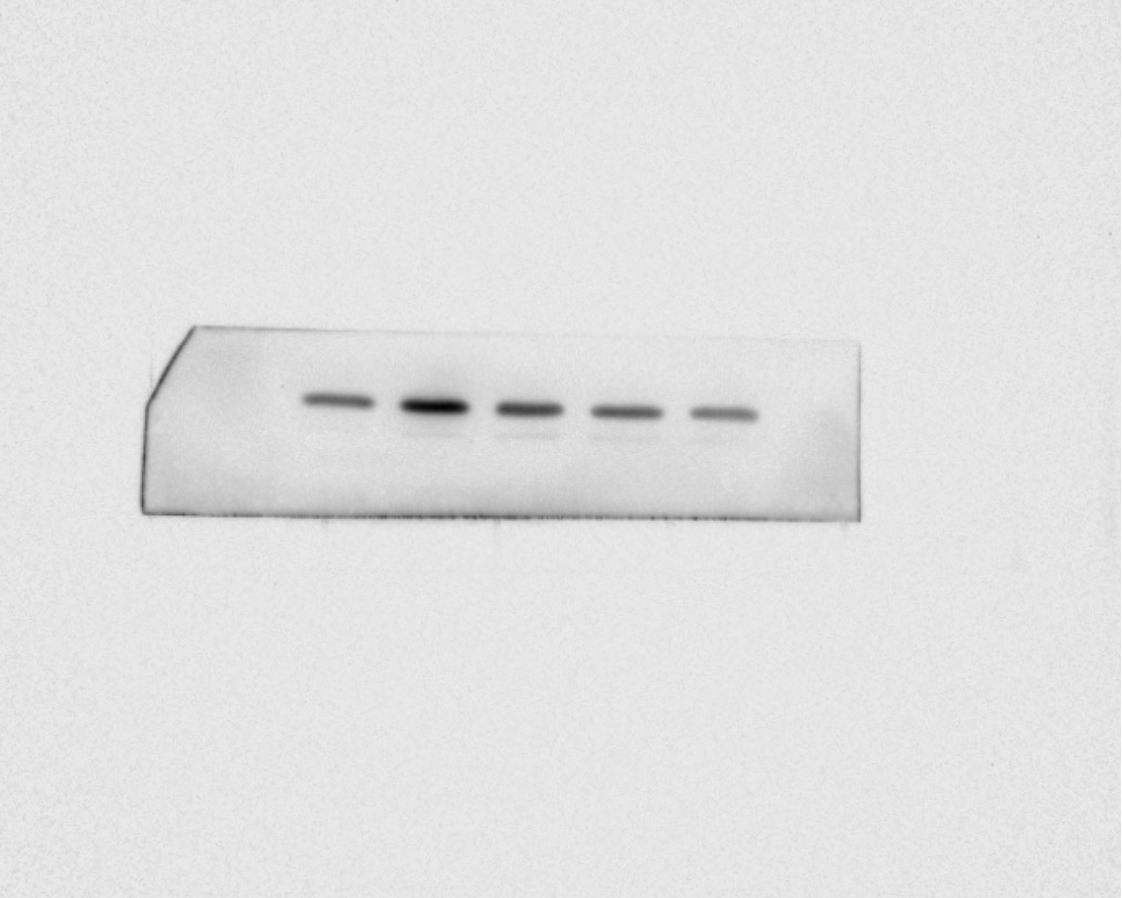

Supplement: Supplementary file 5 [file DataSheet5.zip › Figure 8-WB figure/p-PI3K、PI3K/PCNA 2.tif]

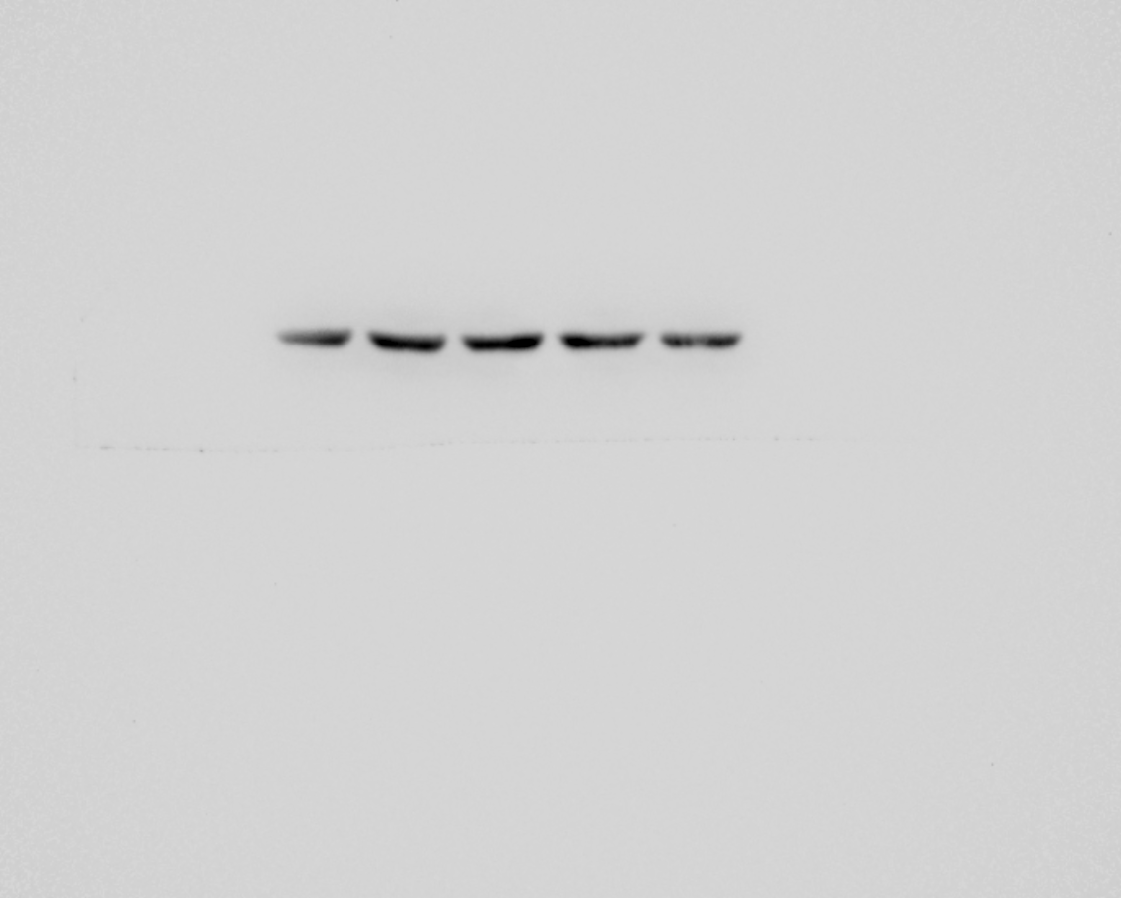

Supplement: Supplementary file 5 [file DataSheet5.zip › Figure 8-WB figure/p-PI3K、PI3K/PCNA 3.tif]

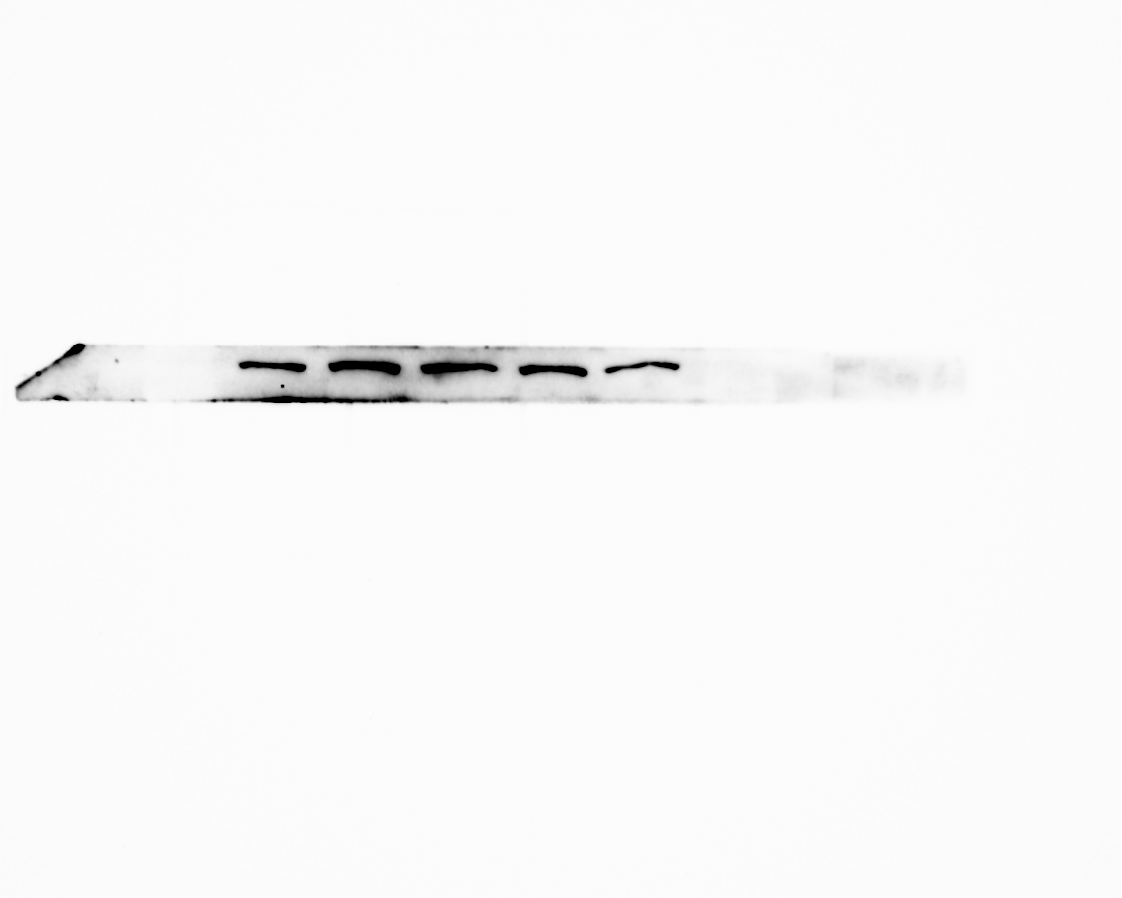

Supplement: Supplementary file 5 [file DataSheet5.zip › Figure 8-WB figure/p-PI3K、PI3K/PI3K 1.tif]

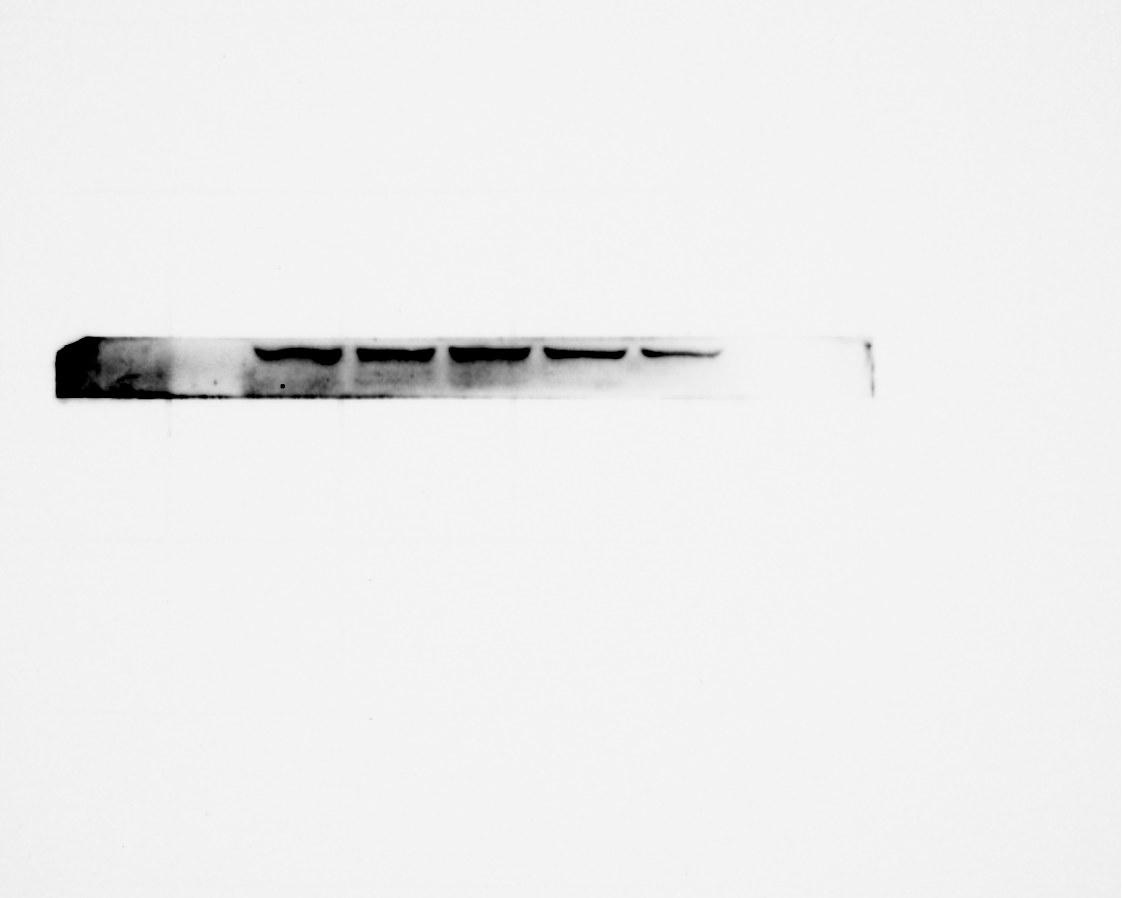

Supplement: Supplementary file 5 [file DataSheet5.zip › Figure 8-WB figure/p-PI3K、PI3K/PI3K 2.tif]

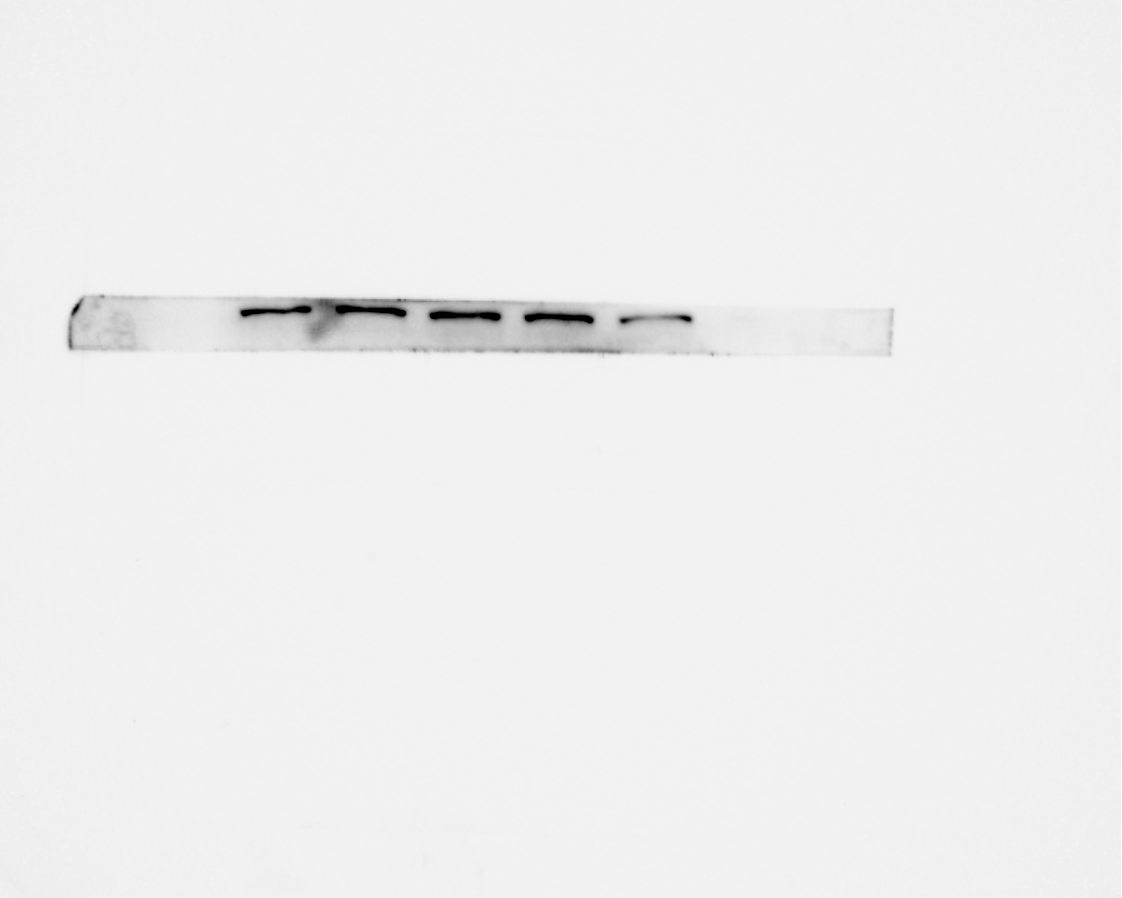

Supplement: Supplementary file 5 [file DataSheet5.zip › Figure 8-WB figure/p-PI3K、PI3K/PI3K 3.tif]

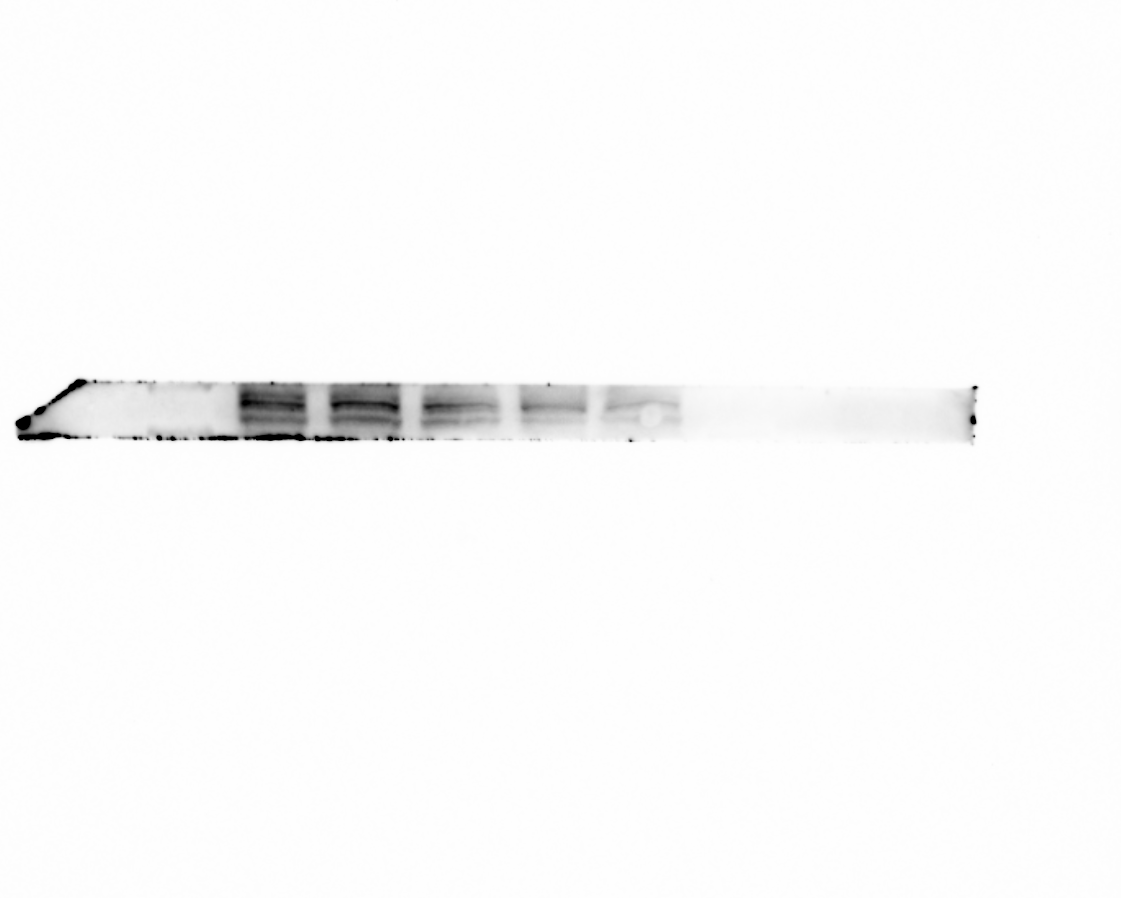

Supplement: Supplementary file 5 [file DataSheet5.zip › Figure 8-WB figure/p-PI3K、PI3K/p-PI3K1.tif]

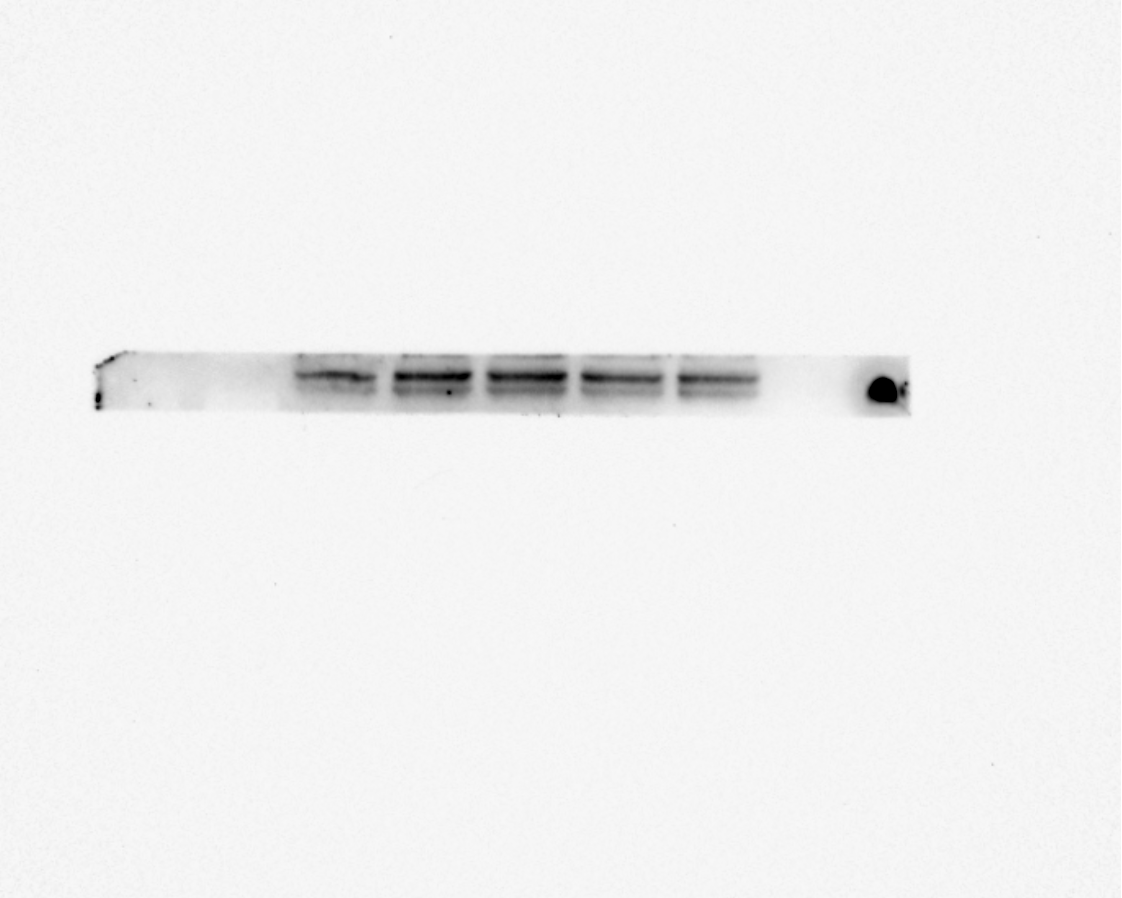

Supplement: Supplementary file 5 [file DataSheet5.zip › Figure 8-WB figure/p-PI3K、PI3K/p-PI3K2.tif]

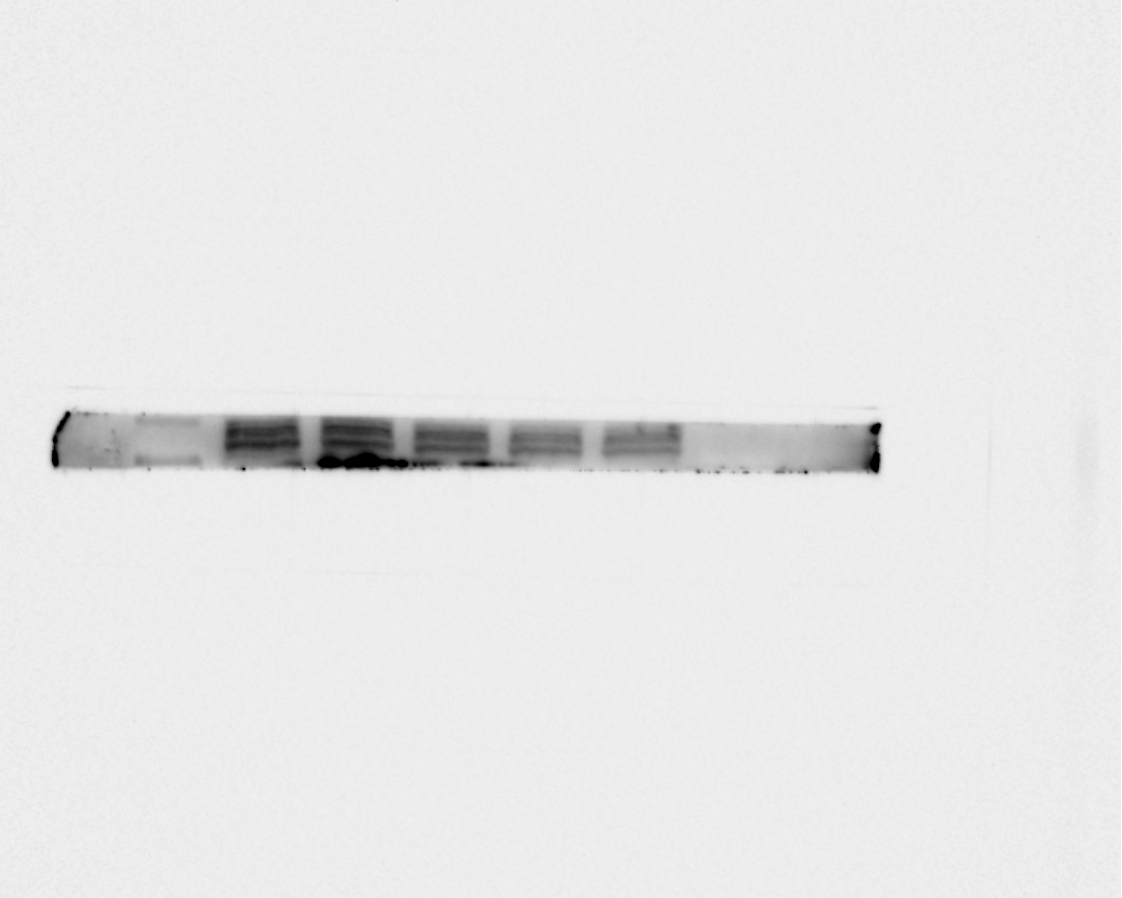

Supplement: Supplementary file 5 [file DataSheet5.zip › Figure 8-WB figure/p-PI3K、PI3K/p-PI3K3.tif]
